# Diet and habitat as determinants of intestine length in fishes

**DOI:** 10.1007/s11160-024-09853-3

**Published:** 2024-04-12

**Authors:** Maria J. Duque-Correa, Kendall D. Clements, Carlo Meloro, Fabrizia Ronco, Anna Boila, Adrian Indermaur, Walter Salzburger, Marcus Clauss

**Affiliations:** 1 Clinic for Zoo Animals, Exotic Pets and Wildlife, Vetsuisse Faculty, University of Zurich, Winterthurerstrasse, 260, 8057 Zurich, Switzerland; 2 School of Biological Sciences, University of Auckland, Private Bag, 92019 Auckland, New Zealand; 3 Research Center in Evolutionary Anthropology and Palaeoecology, Liverpool John Moores University, Byrom Street, Liverpool, L3 3AF UK; 4 Department of Environmental Sciences, Zoological Institute, University of Basel, 4051 Basel, Switzerland; 5 Natural History Museum Oslo, 0562 Oslo, Norway

**Keywords:** Anatomy, Digestion, Dilution, Ecomorphology, Phylogeny

## Abstract

Fish biologists have long assumed a link between intestinal length and diet, and relative gut length or Zihler’s index are often used to classify species into trophic groups. This has been done for specific fish taxa or specific ecosystems, but not for a global fish dataset. Here, we assess these relationships across a dataset of 468 fish species (254 marine, 191 freshwater, and 23 that occupy both habitats) in relation to body mass and fish length. Herbivores had significantly relatively stouter bodies and longer intestines than omni- and faunivores. Among faunivores, corallivores had longer intestines than invertivores, with piscivores having the shortest. There were no detectable differences between herbivore groups, possibly due to insufficient understanding of herbivorous fish diets. We propose that reasons for long intestines in fish include (i) difficult-to-digest items that require a symbiotic microbiome, and (ii) the dilution of easily digestible compounds with indigestible material (e.g., sand, wood, exoskeleton). Intestinal indices differed significantly between dietary groups, but there was substantial group overlap. Counter-intuitively, in the largest dataset, marine species had significantly shorter intestines than freshwater fish. These results put fish together with mammals as vertebrate taxa with clear convergence in intestine length in association with trophic level, in contrast to reptiles and birds, even if the peculiar feeding ecology of herbivorous fish is probably more varied than that of mammalian herbivores.

**Supplementary Information:**

The online version contains supplementary material available at 10.1007/s11160-024-09853-3.

## Introduction

Functional convergence is a key theme in the field of digestive anatomy (Stevens and Hume 1998; Karasov et al. 2011). This is partly due to the intention to derive straight-forward morphological measurements that can be used as proxies for the ecological categorization of species into trophic niches, which may otherwise be more difficult to determine empirically or to observe directly (Douglas and Matthews 1992; Karachle and Stergiou 2010b). Without being able to judge the relative prevalence of this approach across different vertebrate clades, we believe that this approach has been predominantly used in the most species-rich vertebrate clade: the fishes (Barrington 1957; Nikolsky and Birkett 1963; Kapoor et al. 1976; Fänge and Grove 1979; Horn 1989; Kramer and Bryant 1995; Wootton 1998; Clements and Raubenheimer 2006).

In fishes, various morphological traits have been associated with diet specializations and, hence, with their trophic level. These include body shape (e.g. Reis‐Júnior et al. 2023), the volume of the abdominal cavity (e.g. Burns 2021), mouth width and the position of the mouth (e.g. Keppeler et al. 2020), jaw length (e.g. Kopf et al. 2021), tooth shape (e.g. Keppeler et al. 2020), the shape of the oral jaws (e.g. Burress et al. 2020; Ronco et al. 2021), gill raker arrangement and morphology (e.g. Kahilainen et al. 2011), the shape of the lower pharyngeal jaw bone (e.g. Burress 2016; Ronco et al. 2021), the dentition on the pharyngeal jaws (e.g. Hulsey et al. 2020), the presence of a gizzard (Arnette et al. 2023), and in particular the length or the surface area of the intestinal tract (e.g. Wagner et al. 2009; Keppeler et al. 2020; Ghilardi et al. 2021).

In distinguishing faunivores and herbivores, the diet of faunivores is generally considered more easily digestible and hence requiring only a short intestine, whereas plant-based diets are considered more refractory to digestion and hence require a longer intestine (Zihler 1982; Choat et al. 2004; German et al. 2010). The relationship between intestine length and diet has been demonstrated in many different fish groups, for example, in cichlids (Wagner et al. 2009), Characiformes (Burns 2021), Terapontidae (Davis et al. 2013), cyprinids (German et al. 2010), freshwater and estuarine fish (Keppeler et al. 2020), fish from a specific geographical habitat (Kramer and Bryant 1995), and coral reef fish in general (Ghilardi et al. 2021). However, no overarching assessment across a larger taxonomic framework that includes marine and freshwater species is available to date.

Differences in intestinal lengths among trophic groups are so widely accepted that this measurement—often captured as a ratio to the body length (Al-Hussaini 1949)—is commonly used to classify fish species into one of the three conventional trophic levels: herbi-, omni-, or faunivore (Al-Hussaini 1949). The most widespread ratio used in this context is the relative intestine length (RIL), calculated as total intestine length divided by the total body length (tip of the snout to the end of the longer lobe of the caudal fin) or the standard length (excluding the length of the caudal fin) (Jamaluddin et al. 2015). It is generally accepted that the lowest RILs are found in faunivorous species, mid-range values are typical for omnivorous ones, and the highest values are characteristic of herbivores. However, the respective cut-off thresholds differ with respect to the taxonomic groups in consideration, and there are overlaps between trophic groups. To our knowledge, all studies assessing RIL performed so far have focused either on relatively closely related species or species occupying the same environment. One issue with using RIL in fish is the related to the great variability in body shapes, which makes comparisons based on (total or standard) length subject to bias. An alternative measure is Zihler’s index (ZI), which relates gut length to body mass rather than to total or standard length (Zihler 1982), possibly making it a better indicator to evaluate the relationship between body size and intestinal length. Nevertheless, some overlap between trophic groups is also reported for the ZI (e.g. Kramer and Bryant 1995; Karachle and Stergiou 2010a).

In fish, the traditional categorization of faunivores and herbivores, with ‘omnivores’ in between, bears more implications than, for example, among terrestrial mammals. This is because of a comparably larger taxonomic diversity in their prey spectrum (see below). Faunivorous fish, for instance, may consume other vertebrates—mainly other fish—or invertebrates such as mollusks, crustaceans, sponges, or corals, some of which are more difficult to digest than others. For example, although the digestibility of coral matter is unknown, it is assumed to be low due to the high levels of carbonate. In line with the presumed link between diet digestibility and intestine length, it has been shown that, among faunivorous fish, corallivores have comparatively long intestinal tracts (Elliott and Bellwood 2003; Ghilardi et al. 2021). Similarly, arthropods with chitinous exoskeletons may be less easily digestible than fish (German et al. 2010).

Fish herbivory is particularly difficult to categorize (Clements and Raubenheimer 2006). For example, Choat et al. (2002) showed that reef fish species traditionally considered as herbivorous actually consumed quite a range of diets, including detritus. Thus, fish ‘herbivory’ may be directed at ‘photoautotrophs’, which include seagrasses and macroscopic algae, but also comparatively small organisms like microalgae, cyanobacteria, dinoflagellates and diatoms that are often included in the detrital category (Clements et al. 2017). The ingestion of microscopic food sources too small to be recognized and ingested individually has been termed ‘microphagy’ (Arnette et al. 2023). These microscopic food sources may occur on plants (‘epiphytic’), on inorganic surfaces including carbonate reef or rocks (‘epilithic’), within inorganic matrix (‘endolithic’), or associated with sediment (e.g. cyanobacterial mats on sediment) (Cissell et al. 2019). In addition, different terms have been used to describe different or similar aspects of the consortium of microscopic (and partially macroscopic) food, including ‘aufwuchs’, ‘epilithic algal matrix’ (‘EAM’), ‘turf algae’, or microphytobenthos. As a possibly particularly vague term, ‘detritus’ may be used to indicate highly digestible material (Wilson et al. 2003) that is ingested along with indigestible organic components such as dead wood (German 2009) but also inorganic material such as sand (Zgliczynski et al. 2019). In other words, the presumably strict definition of ‘detritus’ as dead organic matter (and microbes) (Bowen 1983) is often not strictly followed when applying the label of ‘detritivore’, even though clearer concepts exist, for example the category of ‘sediment-ingesting detritivore’ (Smoot and Findlay 2010). The mix of microscopic food a fish may ingest during foraging makes it challenging to define specialists, e.g., microalgae versus diatom specialists. A typical differentiation classifies fish targeting food into those that catch or filter food in the water column, those that clip or browse without touching the surface on which their diet grows, those that graze or scrape the surface (epiphytic or epilithic) versus those that scrape deeply (‘excavate’) and therefore ingest both, the epilithic as well as the endolithic material (Vadeboncoeur and Power 2017; Nicholson and Clements 2021).

In the present study, we aimed to collate available data on fish intestine length linked to body mass (and, if available, to body length) to examine its relationships to diet categories and aquatic habitat type (marine versus freshwater). We expected scaling relationships to follow geometry, where a length scales to another length in a linear fashion, and to body mass at a scaling exponent of 0.33 (Calder 1996). We expected to find the classic relationship of faunivores having shortest, and herbivores longest intestines, and hoped to be able to assess diet at a more detailed level. Due to presumed differences in body shape, we expected that body mass would better predict intestinal length than either of the body length proxies. We did not have expectations in relation to marine or freshwater habitat. Additionally, we aimed to compare indices of relative gut length.

## Materials and methods

**Fig. 1:**
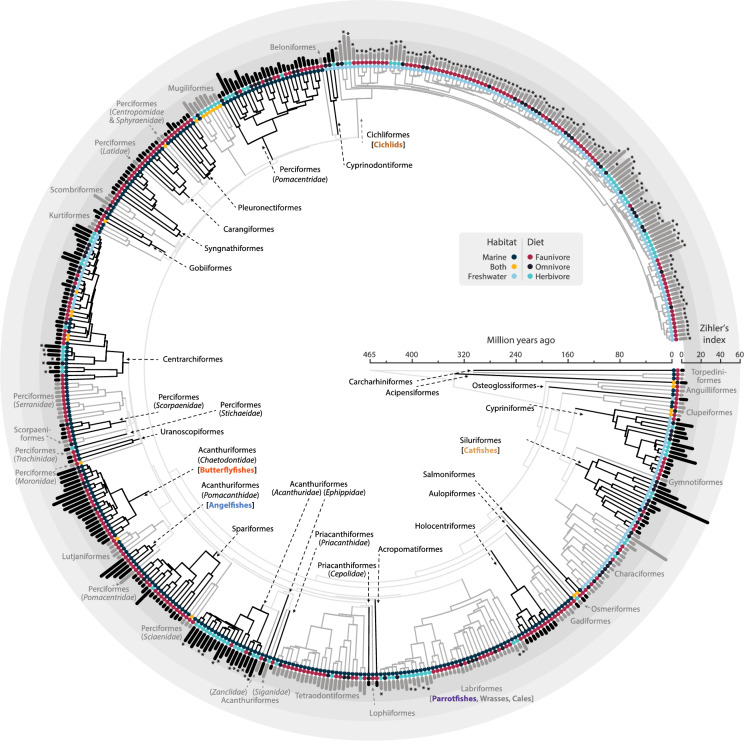
Time calibrated phylogenetic tree with the 468 species of fishes, inner color tip represents habitat, external colored tip represents diet, and the outer bar shows the intestine length expressed as Zihler’s index $ZI= \frac{IL ({\text{mm}})}{10.\sqrt[3]{BM ({\text{g}})}}$ (Zihler 1982)), with grey and black hues to make taxon distinction easier. Species for which original data is reported are marked with an asterisk (*)

The data set for this study consisted of 468 species across 96 families and was based on a combination of available published data (data sets and literature research) and newly sampled specimens (Fig. 1, Table S1).

Data on the intestinal length of cichlid fishes were collected in the framework of several expeditions to Lake Tanganyika between 2010 and 2017 under study permits SP000627 and SP005937 (to F. R.), SP004268 (to A. B.), SP000719 and SP005943 (to A. I.), and SP001994 and SP004273 (to W. S.), issued by the Department of Immigration, Republic of Zambia (see supplementary data for details on samples). Specimens were either collected during scuba diving or snorkeling with gill nets or obtained from local fishermen. Freshly caught specimens were measured (total and standard length [to the nearest mm]), weighed, photographed, and dissected in the field and the intestine length was determined to the nearest mm. In total, we obtained data for 1178 specimens in 117 species of cichlid fishes from Lake Tanganyika, belonging to 16 tribes.

Newly collected data on reef fishes were obtained from dissections performed by KDC’s group under University of Auckland Animal Ethics Committee approvals R717 and AEC2879 and James Cook University of North Queensland Ethics permit A504. Some data pertains to fish collected in New Zealand prior to legal requirements for animal ethics approval for the collection of fish in New Zealand (Clements 1985). In addition, we performed a thorough literature research. To this end, publications on fish intestinal length were searched using Google Scholar. Search terms included “anatomy”, “morphometry”, “digestive tract”, “intestine”, “gut”, and “length”, as well as taxon names. Additionally, the reference lists of identified sources and the references citing identified sources were scrutinized. Data were only included if the species, body mass, and intestinal length were reported.

For stomachless fishes, intestinal length corresponds to the distance from the esophagus to the anus. Additionally, we collected (much less frequent) data on intestine diameter from the same sources for an analysis required in the discussion section. The final dataset included three proxies for body size: body mass (BM, kg), total length (TL, cm), standard length (SL, cm), as well as a corresponding intestinal length (cm) and diameter (cm). We then calculated intestinal indexes, relative intestinal length (RIL for SL and TL), and Zihler’s index (ZI). Finally, means (corrected for sample size) were calculated for each species. The species’ scientific name was taken as per the publication and, when necessary, updated to current nomenclature according to NCBI’s Taxonomy browser (https://www.ncbi.nlm.nih.gov/taxonomy).

Diet information was retrieved from various sources, mainly from the original literature (supplemental information). Notably, trophic categorizations based on intestine length indices were not accepted. All species were categorized into faunivores, herbivores, or omnivores. If the source included quantitative data, a 90% cutoff was used to classify species as faunivore or herbivore; if neither photoautotroph nor animal material was consumed above the cutoff, the species was designated as an omnivore. If the source did not include quantitative information, the category was taken as per the source. Faunivores were further classified into corallivore, piscivore, invertivore, pisci-invertivore, or “other” if there was insufficient information. Similarly, herbivores were further categorized into detritivores, algivores, generalists if they consumed both, or “other” if there was insufficient information to classify them into any of the three categories. Additionally, we collected information on habitat type (marine, freshwater, both) from the original publication and, when necessary, confirmed with FishBase (Froese and Pauly 2023).

A backbone phylogenetic tree containing Actinopterygii was downloaded from Fishtreeoflife.org (Rabosky et al. 2018), and an additional tree including Chondrichthyan fishes was obtained from Vertlife.org (Stein et al. 2018). Both trees were merged into a final ultrametric tree. The time difference between Actinopterygii and Chondrichthyes was set following http://www.timetree.org/ at 464 MYA—CI: (442.7—515.5 MYA); for additional information, see the Supplement. A pruned version of the tree from Ronco et al. (2021) and Matschiner et al. (2020) was used for statistical analyses on Lake Tanganyika cichlids. Trees are provided in the supplementary material.

All statistical analyses were performed in R Studio, version 4.2.1 (R Core Team 2022) using generalized least squares (GLS) and phylogenetic generalized least squares (PGLS), recording the 95% confidence interval for parameter estimates, using the R packages ‘caper’ (Orme et al. 2018) and ‘nlme’ (Pinheiro et al. 2023, Pinheiro and Bates 2000). In all PGLS models, the parameter lambda (λ) was estimated by maximum likelihood. Phylogenetic signals Blomberg’s K and Pagel’s λ were calculated using the R package ‘phytools’ (Revell 2012). Analyses were performed on (i) all available data, (ii) species for which all body size proxies were available (consistent data), (iii) only Lake Tanganyika cichlids, (iv) only faunivores for which all body size proxies were available, (v) only herbivores for which all body size proxies were available, (vi) only marine species for which all body size proxies were available, and (vii) only freshwater species for which all body size proxies were available. The significance level was set to 0.05. To compare models run for the same data subset, we used the small-sample-corrected Akaike information criterion (AICc), considering a difference between models when values are greater than 2 (ΔAICc > 2).

ANOVA and Tukey’s ‘Honest Significant Difference’ method was used to identify differences between the three dietary types within intestinal indexes for data sets with (i) all available data, (ii) species for which all body size proxies were available (consistent data), (iii) only Lake Tanganyika cichlids, (iv) species for which all body size proxies were available but excluding Lake Tanganyika cichlids.

The graphs were made in R Studio (RStudio Team 2020) using the packages ‘ggplot2’ (Wickham 2016) and its extension ‘ggtext’ (Wilke 2020). The package ‘colorspace’ (Zeileis et al. 2020) was used for raincloud plots and ‘ggtree’ (Yu et al. 2017) and ‘phytools’ (Revell 2012) to visualize the phylogenetic tree with annotated data.

## Results

The final database comprised total intestine length and body mass data for 468 species (Fig. 1, Table S1). Total length data were available for 375 species; standard length data for 371 species, and the three size proxies—BM, TL, and SL were available for 293 species. The data set with only Lake Tanganyika cichlids included 117 species. Whereas all species were categorized into the three major trophic categories, a sub-categorization of faunivores was only possible for 265 out of 273 species and for 88 out of 94 herbivore species. The complete dataset is provided in the supplementary materials.

There was a strong phylogenetic signal, as indicated by Pagel’s λ > 0.95, for all body size proxies and for intestine length (Table S1); in terms of Blomberg’s K, the phylogenetic signal was strong for Lake Tanganyika cichlids (K > 0.75), but not for all species (K < 0.60), or all species without the cichlids (Table S1). As expected, the three trophic categories were more heterogeneously distributed across the phylogenetic tree than habitats (freshwater versus marine) (Fig. S1ab).

## Body shape

**Fig. 2:**
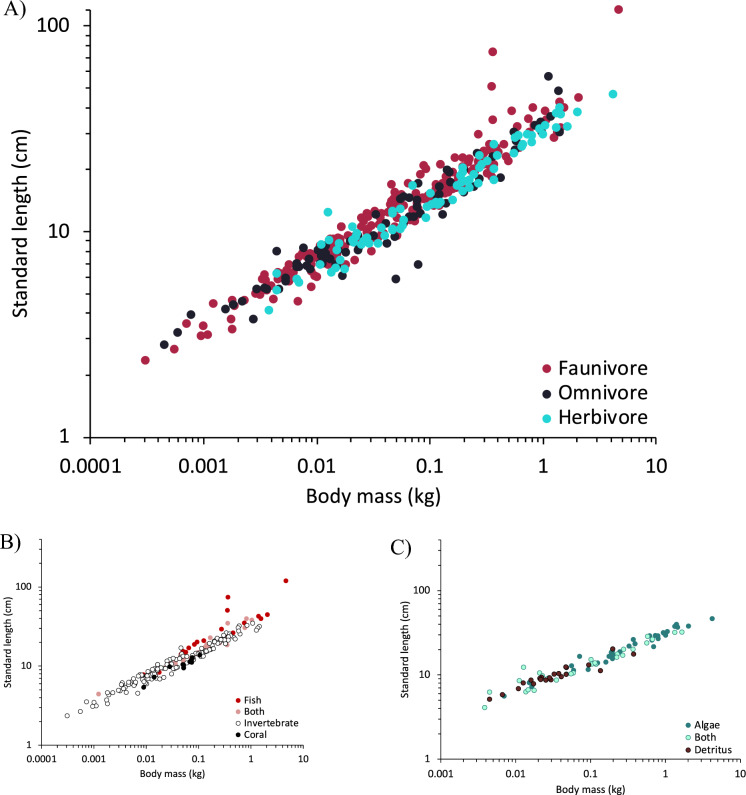
Standard length and body mass relationship according to diet. **A** for all available data using the three dietary categories, **B** for faunivores for which faunivory type could be determined, and **C** for all herbivores for which herbivory type could be determined. Note that the three outliers among the faunivores are the giant moray (*Gymnothorax javanicus*), Chinese trumpetfish (*Aulostomus chinensis*), and Bluespotted cornetfish (*Fistularia commersoni*); the outliers among the omnivores are *Orthospinus franciscensis*, and *Roeboides xenodon*

SL strongly correlated linearly with TL (Fig. S2) and was 11% shorter in GLS and 6% shorter in PGLS than TL (Table S2). Body length measures scaled to body mass at an exponent of 0.33 (Table S2). For the relationship between body length and mass, models that included diet were always among the best-supported (Table S2). Compared to faunivores, herbivorous fish are shorter for their body mass, or, in other words, at the same body length, herbivorous fish are heavier (Fig. 2a). The habitat (marine versus freshwater) generally had no effect on this relationship (Table S2). This pattern did not change if only the consistent dataset was assessed (Table S2). However, for Lake Tanganyika cichlids the diet effect was only significant in GLS but not in PGLS (Table S2).

When assessing faunivorous species only, adding faunivore type (but not habitat) to the models increased model fit (Table S3, Fig. 2b). Compared to piscivores, invertivores and corallivores are shorter for their body mass, or heavier for their length. When assessing herbivorous species only, neither herbivory type nor habitat increased model fit (Table S4, Fig. 2c).

## Intestine length

### Allometric relationships to body mass and length

Intestine length scaled at the expected geometric 0.33 exponent with body mass in GLS for the whole and the consistent datasets, but not for Lake Tanganyika cichlids (which had a higher scaling exponent of 0.53; Table S5). By contrast, in PGLS, the scaling exponent was above 0.33 in all datasets. For the dataset that included information on intestine length and intestine diameter (n = 166 species), length and diameter scaling included 0.33 in the 95%CI in PGLS (Table S6).

Intestine length scaled to body length at the expected geometric exponent of 1.0 in the whole and the consistent datasets in PGLS, whereas the exponents in GLS were mostly lower than 1 (Table S5). By contrast, intestine length scaled with body length at exponents higher than 1.0 in both GLS and PGLS of Lake Tanganyika cichlids (Table S5). In all datasets, body mass as a body size proxy yielded a better data fit than total or standard length (Table S5).

### Trophic level

Body mass was always the body size proxy that yielded the best data fit (Table S7, consistent data).

**Fig. 3:**
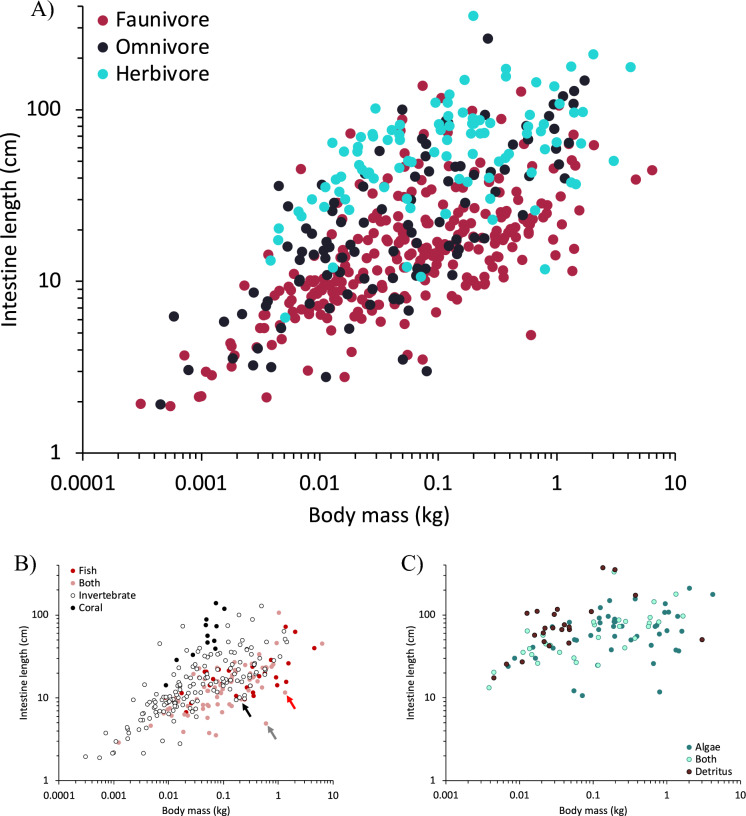
Intestine length and body mass relationship according to diet. **A** for all available data using the three dietary categories, **B** for all faunivores, and **C** for all herbivores. The arrows mark the three species of elasmobranchs *Torpedo torpedo* (purple), *Torpedo marmorata* (grey), and *Scyliorhinus canicula* (black)

Adding trophic level significantly increased model fit in both GLS and PGLS for all data sets and body size proxies. In all data sets and relative to all body size proxies, faunivores had significantly shorter intestines than herbivores, with omnivores in between (Table S7; Fig. 3a).

Among faunivorous fishes, adding faunivore type improved data fit compared to models with body size alone (Table S8). Compared to piscivorous species, invertivores had significantly longer intestinal tracts, and corallivores had even longer ones (Fig. 3b).

Among herbivorous fishes, adding herbivore type did not improve data fit (Table S9). In GLS, detritivores had significantly longer intestines than algivores, but this pattern was not supported in PGLS (Fig. 3c).

### Habitat type

**Fig. 4:**
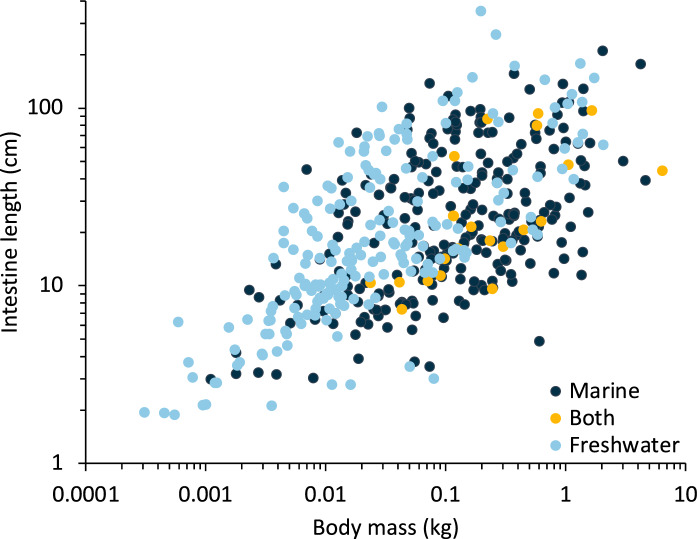
Intestine length and body mass relationship according to aquatic habitat

For the whole dataset, adding habitat information to the intestine length–body size models consistently improved data fit in PGLS (only marginally for body length), and for body mass also in GLS (Table S10). Freshwater fish had significantly longer intestines than marine fish (Fig. 4). In the much-reduced consistent dataset (n = 293), this effect was not present (Table S10).

### Full models

For the whole data set (n = 468 species), models that included diet and habitat had the best support; but distinct model improvement from diet-only models was achieved only in PGLS and using body mass as a size proxy (Table S11). Herbivores and freshwater fish had longer intestines than faunivores and marine fish. For the consistent dataset (n = 293 species), the diet-only models were generally the best-performing (Table S11), with no significant effect of habitat.

Among freshwater fish, a better data fit was always achieved when adding diet to the models, and the model using body mass had the best fit (Table S12). The same was the case among the marine fish (Table S13).

## Intestinal indices

**Fig. 5:**
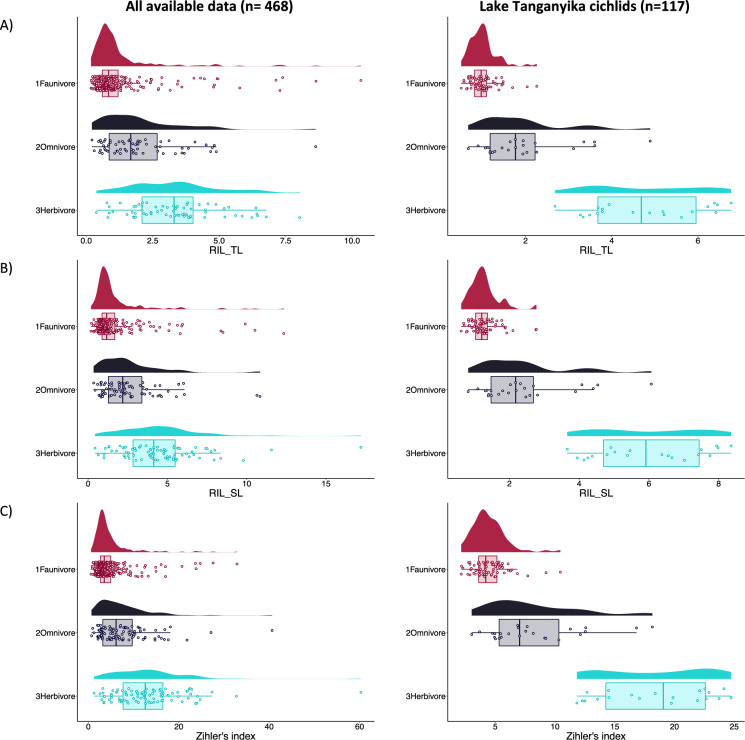
Intestinal indices for all available data and Lake Tanganyika cichlid fishes **A** Relative intestinal length to total length, $RIL\_TL= \frac{IL ({\text{cm}})}{TL ({\text{cm}})}$. **B** Relative intestinal length to standard length $RIL\_SL= \frac{IL ({\text{cm}})}{SL ({\text{cm}})}$. **C** Zihler’s index $ZI= \frac{IL ({\text{mm}})}{10.\sqrt[3]{BM ({\text{g}})}}$ (Zihler 1982). Where IL = intestinal length, TL = total length, SL = standard length, ZI = Zihler’s index, and BM = body mass. Note the outliers are: for all available data (RIL_TL) faunivores ornate butterflyfish* (*Chaetodon ornatissimus*), two spined angelfish* (*Centropyge bispinosa*), flame angelfish (*Centropyge loriculus*), bluelashed butterflyfish* (*Chaetodon bennetti*), and oval butterflyfish* (*Chaetodon lunulatus*). Omnivore mailed butterflyfish (*Chaetodon reticulatus*). Herbivore Lake Tanganyika tilapia (*Oreochromis tanganicae*). For all available data (RIL_SL) faunivores ornate butterflyfish* (*Chaetodon ornatissimus*), two spined angelfish* (*Centropyge bispinosa*), flame angelfish (*Centropyge loriculus*), bluelashed butterflyfish* (*Chaetodon bennetti*), and oval butterflyfish* (*Chaetodon lunulatus*). Omnivore pirapitinga (*Piaractus brachypomus*) Herbivore vermiculated sailfin catfish (*Pterygoplichthys disjunctivus*). And *Pseudosimochromis curvifrons* omnivore outlier among Lake Tanganyika for all indices and additionally *Lestradea perspicax* for ZI. *Corallivore species

For the datasets with all data, consistent data and only cichlids, ANOVA indicated significant differences in the intestinal indices between trophic levels (*P* always < 0.001). Post hoc analyses found significant differences between all dietary categories, with faunivores generally having the smallest intestine-length-to-body-size ratio, followed by omnivores, and herbivores with the highest ratio (Table S14). However, there is an evident overlap between the dietary categories (Fig. 5). Further analysis using only the species with consistent data but excluding Lake Tanganyika cichlids show differences only between faunivores and the other groups, but not between herbivores and omnivores (Table S14). Among the faunivores, several species stood out with exceptionally high indices, mostly corallivores (cf. legend of Fig. 5).

## Discussion

Our study confirms that, across fish, there are convergent evolutionary trajectories in body shape and intestine length with respect to the crude diet categories of fauni-, omni-, and herbivores (Fig. 1, Fig. S6). Among faunivorous fish, a more detailed categorization indicates convergent evolution of body shape and intestine length associated with diets of presumably different digestibility. Among herbivorous fish, an attempt at demonstrating a similar convergence for more detailed diet categories was not successful. To our knowledge, the observation that marine fish generally have shorter intestines than freshwater fish has so far not been reported.

## Limitations of the present study

Several limitations of the present study should be mentioned beforehand. The deliberate constraint of only accepting data that included body mass meant that a large body of literature that only extrapolates body mass from body length could not be used (e.g. Keppeler et al. 2020). In contrast to what is typically done in mammalian and avian anatomy (Duque-Correa et al. 2021, 2022), but similar to reptile anatomy tradition (Hoppe et al. 2021), body mass has often not been recorded in fish studies (Alencar et al. 2022), possibly because speedy processing of many individuals on vessels is simpler when only length data is taken as a body size proxy. However, for the investigation of the relationship between body length and mass as a shape proxy, as well as for future comparisons with other vertebrate clades, body mass is indispensable. Other general constraints typical for large datasets such as ours apply, including body condition of the specimens investigated, potentially influenced by season and habitat, age, sex, and possibly even growth stage, which could not be consistently controlled for and may add noise to the dataset.

Groups of fish whose intestinal tract comprises a ‘spiral intestine’ are under-represented in this study, including sharks and rays (elasmobranchs), lungfishes (Ceratodontiformes), bichirs (Polypteriformes), sturgeons (Acipenseriformes), gars (Lepisosteiformes) and bowfins (Amiiformes) (Argyriou et al. 2016). While spiral intestines have been described in terms of their morphology, including the number of turns (Argyriou et al. 2016), we are unaware of publications that estimate the length of their lumen. Intestine length data for species with this feature necessarily underestimates the actual intestinal length (Karachle and Stergiou 2010a). At least in sharks, the morphology of the spiral intestine itself does not contain a dietary signal (Leigh et al. 2021). The only shark included in our dataset, the small-spotted catshark (*Scyliorhinus canicula*), is at the lower range of intestine length for its body mass among faunivorous fish, and the two electric rays (*Torpedo* spp.) appear as even more distinct outliers (arrows in Fig. 2b). The only sturgeon included in our dataset (*Acipenser transmontanus*) is considered an omnivore; when compared to other omnivores, it ranks among those with a relatively shorter intestine, but not as exceptional as the rays in the faunivore dataset (Fig. S3). If more of the predominantly faunivorous sharks and rays were included with their actual internal digestive tract length in a study such as ours, an overall weaker link between intestine length and diet would be expected.

Compared to studies in other vertebrate groups (Duque-Correa et al. 2021, 2022; Hoppe et al. 2021), data on the intestinal tract of fish generally does not differentiate between individual intestinal sections. Such differentiation can be done for individual taxa, especially when supplemented by histological data (e.g. Johnson and Clements 2022). A macroscopic differentiation of sections is also possible, for example, when hindgut chambers are present (as is the case of angelfish [*Pomacanthidae*] or chubs [*Kyphosidae*]), or when sacculated versus non-sacculated intestinal sections exist (as in parrotfish [Labriformes: *Scaridae*]) (Clements and Raubenheimer 2006; Clements and Choat 2018). However, there are no easy macroscopic morphological hallmarks or nomenclature to differentiate intestinal sections across fish in general.

Potentially the most important limitation is the lack of a consistent, quantitative record of fish diet items spanning all taxa (Clements and Raubenheimer 2006).

## Body shape

Due to geometric considerations, total and standard lengths are expected to scale linearly, and body length is expected to scale to body mass 0.33 (Froese 2006). This was demonstrated in mammals (Silva 1998), lizards (Meiri 2010) and reptiles (Hoppe et al. 2021). For fish, this exponent is often included in the confidence interval of intraspecific studies (Froese 2006), and is corroborated interspecifically in the present study. The relationship between body length and mass is an—albeit crude—proxy for body shape: animals that are shorter at the same body mass, or heavier at the same body length, must necessarily be ‘stouter.’ Since herbivorous animals are expected to harbor larger guts they should have such a stouter appearance; this is a parsimonious explanation for the effect of trophic group on the mass-length relationship across fish in the present study. In mammals, differences between taxa in the length-mass relationship have been demonstrated (Silva 1998), but this has not been tested for trophic groups to our knowledge. For lizards, a tendency for herbivorous species to be shorter at the same body mass was reported (Meiri 2010), but in a sample comprising all reptile clades, this pattern was not supported when accounting for phylogeny (Hoppe et al. 2021).

The trophic level is, thus, one of the many other factors influencing the body shape of fish (reviewed in Alencar et al. 2022). Various groups have described fish with more difficult-to-digest diets as having a more voluminous body cavity (Burns 2021), deeper bodies (Keppeler et al. 2020) and/or a bulkier shape (Reis‐Júnior et al. 2023) in order to accommodate a longer or more voluminous intestine. The slimmer shape of faunivorous fish has been interpreted as an adaptation for pursuit hunting, even if some faunivores with a sit-and-wait strategy may have deeper bodies; by contrast, stouter body shapes, though less suitable for high speeds, have a higher maneuverability (Webb 1984a, b). Thus, digestive physiology and locomotory adaptations most likely jointly determine body shape.

Similar observations have been made across mammals, where herbivores have larger body cavities than faunivores (Clauss et al. 2017). The observation that this difference was not evident in the few studied synapsids, which are considered ancestral to the line of mammals, led to the speculation that the differentiation between the trophic guilds also reflected a predator–prey arms race of agility and maneuverability—in the sense that faunivores use the opportunity for a slim body shape offered by their trophic niche (De Cuyper et al. 2020). Larger body cavities in herbivores than faunivores were also observed across a large sample of extant and fossil tetrapods by Maher et al. (2022).

In the present study, differences in the length-mass relationship were additionally evident across faunivorous fish, again supporting the dual interpretation of digestive physiology (with invertebrate diets being less easily digestible than piscivorous diets) and locomotion (with invertebrates easier to prey upon than fish) (Fig. 2b). A more detailed categorization of different invertebrate prey might reveal even more functional differences, as some invertebrate prey, such as sponges, gastropods or corals, are not elusive like other invertebrates such as squid or many crustaceans.

The fact that no similar differentiation was observed among fish classified as herbivores suggests that (a) current dietary categories for herbivores do not satisfactorily reflect relevant differences in the relationships between digestive physiology and “herbivore” diets, and/or (b) foraging-related locomotion does not necessitate the same degree of differentiation when the food is represented by a group of universally immobile organisms. Nevertheless, body shape differences have also been noted among closely related herbivorous species, such as rabbitfish, which would have to be explained by more detailed locomotory and food accession characteristics (Zolkaply et al. 2021).

Concerning extremely elongated body shapes, as in the moray eel, trumpetfish, and cornetfish, the pattern of body length versus intestine length compared to other fish resembles that observed in snakes compared to other reptiles (Fig. S4). These sparse data suggest that these fish and snakes resemble each other, having shorter intestines than expected for their body length (Hoppe et al. 2021). This contrasts with elongated mammals of the mustelid family that have—for their size (body mass)—distinctively longer intestines (McGrosky et al. 2016).

Due to the variation in body length across fish of similar body mass, it is understandable that body mass provides a better proxy for body size as related to intestine length, as it is the whole mass, not a length, that needs to be fueled by the work of the digestive tract (Kramer and Bryant 1995; this study).

## Intestine length

### Allometric scaling

Scaling of intestine length with body mass at a higher exponent than the geometrically expected 0.33, i.e., positive allometry of intestine length, has previously been described in mammals, reptiles, and birds (Duque-Correa et al. 2021, 2022; Hoppe et al. 2021). In all PGLS analyses of the present study, the scaling exponents were higher than the geometric 0.33. It has been suggested that this is an effect of geometric scaling of intestinal surface area, but because of the importance of minimizing the distance between the luminal digesta and the site of absorption, intestinal diameter might scale less-than-geometrically (at negative allometry), with length necessarily compensating by positive allometry. To test this hypothesis, comparative data on intestinal diameter is required. In fish, such data have been compiled on a large scale for coral reef fishes (Ghilardi et al. 2021), allowing a preliminary test of this hypothesis with a set of 166 species. In that subset of species, the positive allometry of intestine length and the postulated negative allometry of intestine diameter was not confirmed (Table S6). More data are required (for the same number of species that also yield positive length allometry) to test this hypothesis, or alternative explanations must be sought.

### Trophic categories

The distinct effect of trophic category on intestine length corroborates numerous previous studies on smaller datasets of fish species (cf. Introduction). On the one hand, it can be argued that the repeated failure to detect finer-scale relationships between diet and intestine length (and also in the herbivorous fish of the present study) is linked to the uncertainty of species-specific diets at finer scale (Clements and Raubenheimer 2006). On the other hand, it may well be linked to our currently restricted understanding of the digestibility of diet items relevant for fish—in particular, those categorized as herbivores (Clements et al. 2009). The basic assumption underlying any interpretation of intestinal length pattern is that longer intestines relate to more difficult-to-digest diets. This appears intuitive in the general faunivore-herbivore comparison. These repeated results in the fish literature constitute an incentive to test the digestibility of fish diet components in a comparative way, to add substance to this skeletal finding of convergence.

However, two possible, non-exclusive scenarios cannot be disentangled by the morphometric comparative approach: Is the dilution of easily digestible components by basically indigestible or difficult-to-digest material the driver of increased gut length, where a longer gut enhances the encounter rates of the consumer’s digestive enzymes with the targeted, easily digestible nutrients (dilution hypothesis)? Or, alternatively, is the difficult-to-digest material itself the nutritional target, and the longer intestine is required to increase the exposure time to digestive enzymes, thus improving digestive efficiency (Horn 1989) (digestive resistance hypothesis)? Of course, both scenarios might apply concomitantly.

The dilution hypothesis scenario relates to the feeding mode. Of two species targeting similar microscopic food sources, one collecting it as epilithic material, with a limited amount of scraped inorganic matter ingested during foraging, will have less dilution with indigestible components compared to an excavating species that forages for endolithic material and hence have a higher degree of that dilution. This difference should, in theory, lead to different adaptations, including amongst others the intestine length, even though the same hypothetical nutrient source is targeted (e.g. epilithic or endolithic microscopic photoautotrophs and heterotrophs).

The within-faunivore comparison (Fig. 3b) benefits from a potentially simple categorization of food digestibility: invertebrates can be assumed to contain a higher proportion of difficult-to-digest material, e.g. in the form of a chitin exoskeleton, compared to fish, and corallivory can be assumed to result in a particularly high proportion of indigestible material in the ingested matter. The same logic applies to the interpretation of why scale eating cichlids have longer intestines than related piscivores (Wagner et al. 2009). Experimentally, for example, the dilution of easily digestible nutrients by fibre led to instine elongation in zebrafish (*Danio rerio*) (Leigh et al. 2018). In these cases, differentiating conclusively between the ‘dilution hypothesis’ and the ‘digestive resistance hypothesis’ would require an actual digestibility measurement. Recently, Herrera et al. (2022) used such an approach, feeding a carnivorous fish (*Anoplarchus purpurescens*) a carnivore or an omnivore diet (the latter containing algae). They found an increase in intestine length on the omnivore diet, yet demonstrated via stable isotope data that the algae were not digested, supporting the dilution hypothesis.

**Fig. 6:**
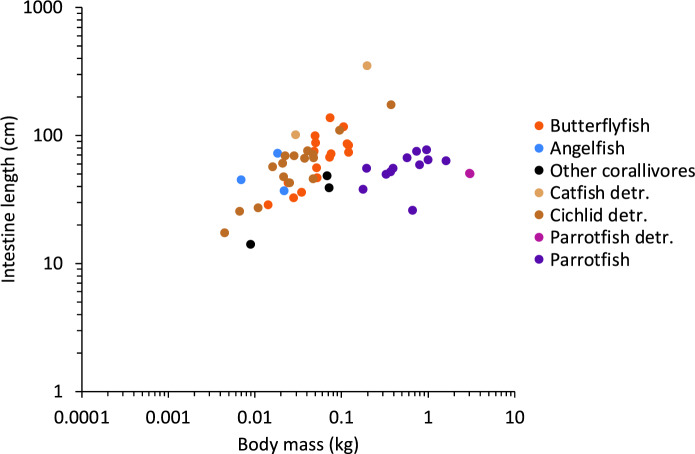
Intestine length and body mass relationship highlighting species with difficult-to-digest diets. Highlighted are the corallivore butterflyfish, parrotfish, and angelfish, as well as detritivore parrotfish, catfish, and cichlids. Other corallivores are the threeband pennantfish (*Heniochus chrysostomus)*, Johnston Island damsel (*Plectroglyphidodon johnstonianus*), and the broom filefish (*Amanses scopas*)

The case of those faunivores categorized as corallivores could, in terms of the dilution hypothesis, resemble that of those so-called ‘detritivorous’ species that ingest a high proportion of indigestible material along with the targeted food. Comparing the faunivorous corallivorous butterflyfish (*Chaetontidae*) and angelfish (*Pomacanthidae*) of the present study to “wood-eating” hypostomid catfish (which scrape biofilm off dead wood) (German 2009), or “geophagous” cichlids (which ingest invertebrate prey in sediment) (Muschick et al. 2012) supports this hypothesis (Fig. 6). Also supporting the dilution hypothesis is the finding that among butterflyfishes, planktivores (that take food out of the water column without background material) have distinctively shorter intestines than obligate corallivores (Berumen et al. 2011). The simple dilution of ingested food with (for the specific consumer) indigestible components could thus represent an important driver of intestine length evolution in fish—but not necessarily for all taxa. Parrotfish (Labriformes: Scaridae), which also consume high levels of indigestible material during their scraping or excavating foraging (Clements and Choat 2018), do not conform to the pattern of particularly long intestines (Fig. 6). Possibly, members of this group (i.e. scarinine genera and the sparisomatinine genus *Sparisoma*) evolved their sacculated intestines as an alternative adaptation to a high dilution of digesta with indigestible material (Clements and Choat 2018). Other potential adaptations for dealing with diluting indigestible compounds include pre-intestinal mechanisms that use the density-based potential to separate indigestible sediment from organic detritus (Bowen 1983; Smoot and Findlay 2010).

In addition, such scenarios may still become more complex depending on whether digestion of the nutrient source is mainly based on the consumer’s enzymes, or whether this is partly done by a symbiotic intestinal microbiome (Clements et al. 2014). The many forms of photoautotroph plant matter available to fish—seagrasses, macroalgae, filamentous algae and microalgae, cyanobacteria, dinoflagellates, diatoms, and any form of detritus from these organisms—may require distinct adaptations for optimal digestion. Some photoautotrophic food sources might be favorably digested by a symbiotic microbiome, while others may be digested efficiently by the fish’s enzymes. For this latter auto-enzymatic digestion, a thin tubular structure appears most suited to ensure short distances between the site of enzyme secretion and the digesta, and between digesta and the site of absorption. With respect to the dilution hypothesis, this corresponds to the observation of long yet narrow intestines in corallivorous coral reef fish by Elliott and Bellwood (2003). For allo-enzymatic digestion, where the enzyme-secreting microbes are within the digesta, larger intestine volumes may be more beneficial, delaying digesta retention in a compartment that allows the microbes to replicate. Again, parrotfish appear as outliers to the rule, given their sacculated intestines yet basic reliance on auto-enzymatic digestion (Clements and Choat 2018). Fish taxa in which hindgut contents, as a sign of microbial fermentation, contain higher concentrations of short-chain fatty acids (Clements et al. 2014, 2017) do not stand out in terms of intestine length among the herbivores (Fig. S5). No simple rule regarding gut length seems to be applicable.

Whether the two different digesta properties–diluting indigestible components, or components best digested by a microbiome—do occur simultaneously in a fish taxon, or whether such a combination is ecologically or physiologically unlikely, remains to be investigated. To date, no excavators that use fermentative digestion have been described, to our knowledge. Notably, a distinct contribution of a symbiotic microbiome to digestion was mainly reported for consumers of macroalgae, especially phaeophytes (Clements et al. 2014, 2017). Nevertheless, fermentative activity has also been demonstrated in fish species considered planktivorous, possibly related to the consumption of faeces of predatory fish species (Clements and Choat 1995; Choat et al. 2004).

In the present study, we did not attempt to categorize omnivores further; for coral reef fishes, Kramer and Bryant (1995) showed that intestine length did not yield a differentiating signal within omnivores and suggested that it worked best as a proxy for broad trophic niche definitions.

### Intestinal indices for trophic level

The Zihler index was initially developed to eliminate bias in intestinal indices based on body length due to different body shapes (Zihler 1982). Without actual validation statistics (testing how often a correct classification occurs based on each of the indices), we cannot decide whether this is really the case. Both the inspection of Fig. 5 as well as the ratio of the largest mean difference (herbivore-faunivore) divided by the smallest mean difference (omnivore-faunivore), being 3.0 for both, the relative intestinal length based on total length and the Zihler index for the complete dataset, do not indicate a particular superiority of the latter.

Although the use of intestinal indices has a long history in fish biology (Al-Hussaini 1949; Zihler 1982), the fact that these indices overlap for trophic groups has also been noticed from early on, and that they are most reliably used within taxonomic (e.g. Elliott and Bellwood 2003) or body size boundaries (e.g. Kramer and Bryant 1995). It is well recognized that a statistically significant difference between trophic groups with respect to an index does not necessarily translate into a powerful proxy (Clauss 2021). Therefore, a prudent use of these indices for the derivation of a trophic categorization is required, where such an index is only one of several pieces of contributing evidence (e.g. Liedke et al. 2016).

### Marine versus freshwater

The most surprising result of the present study was the observation that freshwater fish generally tend towards longer intestines compared to marine fish (Fig. 4). To our knowledge, no physiological basis for this difference has been described. If anything, we would have expected the intestine of marine fish to be involved in more osmoregulation (Grosell 2010), which would have led to the simplistic prediction of longer intestines in this group. The only study we are aware of that tests for an effect of shifting fish to salt water suggests an increase, not a decrease, in intestinal tissue (MacLeod 1978). Hence, other hypotheses are warranted to explain our observation. Perhaps a systematic difference between organic detritus from terrestrial vascular plants and mangroves on the one hand, and algae on the other hand, is involved: algal detritus is less refractory to digestion (Rice and Tenore 1981; Alongi and Christoffersen 1992). In a similar direction, a recent comparative study revealed distinct differences in the gut microbiome between freshwater and marine fish (Kim et al. 2021). However, given that habitat was only a significant factor in the most extensive dataset, and the overlap in intestinal length between freshwater and marine species was substantial (Fig. 4), this result possibly need not be over-emphasized. In the model that included both diet and habitat, intestinal length was longer for omnivores and herbivores compared to faunivores and freshwater species had longer intestinal tracts (Table S11).

## Conclusions

In terms of a dietary signal in intestinal morphometrics, fish resemble mammals showing convergence across trophic groups, even if this may well be due to different physiological selective pressures; this contrasts with the situation reported for reptiles and birds. The reasons for these differences between the vertebrate clades remain to be investigated.

For a more comprehensive analysis of the macroevolution of morphophysiological adaptations of fish to trophic niches, dietary categorizations other than the simplistic fauni-, omni- and herbivore, on which the present study largely relies, will be required. Detailed observation may be necessary of what kind of material reaches which part of the digestive tract in what proportions. Inorganic sediment may be initially ingested and expelled orally or passed on to the lower digestive tract. Depending on the feeding mode, the proportions of such indigestible material may vary. Organic material may represent the nutritional target or only the substrate on which the actually targeted epiphytic photoautotrophs or heterotrophs are located. Depending on the consumer species, ingested material may be auto-enzymatically digestible or not. For consumers without a symbiotic microbiome, the latter category may thus also represent indigestible dilutant, whereas it represents a nutritional target for consumers with such an intestinal microbiome. Transitions between these states may be gradual rather than categorical. Such fine-scaled observations have been made for various individual fish taxa. Expanding these to a generalized applicable scheme and set of parameters is an important challenge for fish evolutionary ecology.

### Supplementary Information

Below is the link to the electronic supplementary material.

Supplementary file1 (PDF 1999 kb)

## Data Availability

The data collection including all individually recorded data and the species average values, biological characteristics, the corresponding literature references, and the phylogenetic tree used are available as online supplements. The R code used in the statistical procedures has been referenced in the method section.
