# Supplementary material for: Diet and habitat as determinants of intestine length in fishes
Source: Rev Fish Biol Fish. 2024 Apr 12;34(3):1017–34. doi: 10.1007/s11160-024-09853-3 (PMC11297901; doi:10.1007/s11160-024-09853-3)
Supplement: Supplementary file 1 — Supplementary file1 (PDF 1999 kb) [file 11160_2024_9853_MOESM1_ESM.pdf]

Supplementary material

Diet and habitat as determinants of intestine length in fishes

Maria J. Duque-Correa • Kendall D. Clements • Carlo Meloro • Adrian Indermaur • Fabrizia Ronco • Anna Boila • Walter Salzburger • Marcus Clauss

Tables S1-S14
Figures S1-S6
Phylogenetic tree considerations
References (for intestine and diet data)

Table S1 Phylogenetic signals (K and λ)

	All available data			Lake Tanganyika cichlids			All data excluding Lake Tangayika cichlids		
	n	K	λ	n	K	λ	n	K	λ
Body mass	468	0.33	0.97	117	0.76	0.96	351	0.30	0.96
Total length	371	0.44	0.97	117	0.84	0.94	258	0.44	0.97
Standard length	375	0.61	0.96	117	0.87	0.95	200	0.57	0.94
Intestine length	468	0.15	0.97	117	0.84	0.88	351	0.24	0.98

analyses performed using 'phytools' with 9999 simulations per analysis and log-transformed values: all analyses significant at $P < 0.001$

Table S2 Summary statistics for models assessing fish **body size** proxies (body mass (BM), standard length (SL) or total length (TL)) alone, or additionally with a **diet** proxy or/and **aquatic habitat** (freshwater or marine) according to log= a + b log (BM) + c (diet proxy) + d (water); (significant parameters in **bold**, almost significant parameters (0.051 – 0.10) in *italics*).

			**GLS**				**PGLS**			
**Model**		**n**	**AICc**	**ΔAIC**		**parameter (95%CI)**	**lambda (95%CI)**	**AICc**	**ΔAIC**	**parameter (95%CI)**
All data										
SL	TL	371	-1207.4	-	a	**-0.11 (-0.12 to -0.09)**	0.88 (0.75 to 0.94)	-1303.5	-	**-0.06 (-0.11 to -0.01)**
					b	**1.01 (0.99 to 1.02)**				**0.99 (0.97 to 1.01)**
TL	BM	375	-755.7	0.0	a	**1.61 (1.59 to 1.63)**	0.99 (0.98 to 0.99)	-1035.0	4.4	**1.68 (1.43 to 1.93)**
					b	**0.32 (0.31 to 0.34)**				**0.32 (0.31 to 0.33)**
TL	BM + diet	375	-754.5	1.2	a	**1.62 (1.61 to 1.64)**	0.99 (0.98 to 0.99)	-1039.4	0.0	**1.68 (1.43 to 1.93)**
					b	**0.33 (0.32 to 0.34)**				**0.32 (0.31 to 0.33)**
					Omnivory	-0.01 (-0.04 to 0.01)				0.003 (-0.01 to 0.02)
					Herbivory	**-0.05 (-0.07 to -0.03)**				**-0.02 (-0.04 to -0.004)**
TL	BM + water	375	-740.4	15.3	a	**1.61 (1.59 to 1.62)**	0.99 (0.98 to 0.99)	-1033.4	6.0	**1.68 (1.43 to 1.93)**
					b	**0.33 (0.31 to 0.34)**				**0.32 (0.31 to 0.33)**
					Both	0.01 (-0.05 to 0.08)				-0.05 (-0.12 to 0.02)
					Freshwater	0.01 (-0.01 to 0.03)				-0.02 (-0.13 to 0.09)
TL	BM + diet + water	375	-740.5	15.2	a	**1.63 (1.61 to 1.64)**	0.99 (0.98 to 1.00)	-1037.6	1.8	**1.68 (1.44 to 1.93)**
					b	**0.33 (0.32 to 0.35)**				**0.32 (0.31 to 0.33)**
					Omnivory	-0.02 (-0.04 to 0.01)				0.00 (-0.01 to 0.02)
					Herbivory	**-0.05 (-0.07 to -0.03)**				**-0.02 (-0.04 to 0.004)**
					Both	0.01 (-0.04 to 0.07)				-0.05 (-0.12 to 0.02)
					Freshwater	0.02 (0.00 to 0.04)				-0.02 (-0.13 to 0.09)
SL	BM	371	-861.0	10.4	a	**1.51 (1.50 to 1.52)**	0.98 (0.97 to 0.99)	-1153.7	6.2	**1.61 (1.50 to 1.72)**
					b	**0.33 (0.32 to 0.34)**				**0.32 (0.31 to 0.33)**
SL	BM + diet	371	-860.4	11.0	a	**1.53 (1.51 to 1.54)**	0.98 (0.96 to 0.99)	-1159.9	0.0	**1.62 (1.51 to 1.72)**
					b	**0.33 (0.32 to 0.34)**				**0.32 (0.31 to 0.33)**
					Omnivory	**-0.02 (-0.04 to 0.00)**				*-0.01 (-0.02 to 0.01)*
					Herbivory	**-0.04 (-0.06 to -0.02)**				**-0.03 (-0.04 to -0.01)**
SL	BM + water	371	-864.4	7.0	a	**1.51 (1.49 to 1.52)**	0.98 (0.97 to 0.99)	-1152.5	7.4	**1.62 (1.51 to 1.74)**
					b	**0.34 (0.33 to 0.35)**				**0.32 (0.31 to 0.33)**
					Both	-0.01 (-0.05 to 0.03)				*-0.04 (-0.08 to 0.01)*
					Freshwater	**0.04 (0.02 to 0.06)**				-0.03 (-0.08 to 0.02)
SL	BM + diet + water	371	-871.4	0.0	a	**1.53 (1.51 to 1.54)**	0.98 (0.96 to 0.99)	-1158.4	1.5	**1.63 (1.52 to 1.74)**
					b	**0.35 (0.33 to 0.36)**				**0.32 (0.31 to 0.33)**
					Omnivory	**-0.03 (-0.05 to -0.01)**				-0.01 (-0.02 to 0.01)
					Herbivory	**-0.04 (-0.06 to -0.03)**				**-0.03 (-0.04 to -0.01)**
					Both	0.00 (-0.04 to 0.04)				-0.04 (-0.08 to 0.01)
					Freshwater	**0.05 (0.03 to 0.06)**				-0.03 (-0.08 to 0.02)

Faunivory used for comparison when dietary proxies used.

Table S2 ctd. Summary statistics for models assessing fish **body size** proxies (body mass (BM), standard length (SL) or total length (TL)) alone, or additionally with a **diet** proxy or/and **aquatic habitat** (freshwater or marine) according to log= a + b log (BM) + c (diet proxy) + d (water); (significant parameters in **bold**, almost significant parameters (0.051 – 0.10) in *italics*).

			GLS				PGLS			
**Model**		**n**	**AICc**	**ΔAIC**		**parameter (95%CI)**	**lambda (95%CI)**	**AICc**	**ΔAIC**	**parameter (95%CI)**
Consistent data (species for which all body size proxies are available										
SL	TL	293	-1207.4	-	a	**-0.11 (-0.12 to -0.09)**	0.88 (0.76 to 0.94)	-1303.5	-	**-0.05 (-0.11 to -0.01)**
					b	**1.01 (0.99 to 1.02)**				**0.99 (0.97 to 1.01)**
TL	BM	293	-709.1	14.6	a	**1.61 (1.59 to 1.62)**	0.97 (0.95 to 0.99)	-932.5	53.1	**1.71 (1.60 to 1.82)**
					b	**0.33 (0.32 to 0.34)**				**0.33 (0.32 to 0.34)**
TL	BM + diet	293	-708.4	15.3	a	**1.62 (1.61 to 1.64)**	0.97 (0.94 to 0.99)	-940.7	44.9	**1.71 (1.60 to 1.82)**
					b	**0.33 (0.32 to 0.34)**				**0.33 (0.32 to 0.34)**
					Omnivory	-0.01 (-0.04 to 0.01)				0.00 (-0.01 to 0.01)
					Herbivory	**-0.04 (-0.06 to -0.02)**				**-0.03 (-0.05 to -0.01)**
TL	BM + water	293	-709.3	14.4	a	**1.61 (1.60 to 1.62)**	0.97 (0.95 to 0.99)	-928.8	56.8	**1.72 (1.60 to 1.83)**
					b	**0.35 (0.33 to 0.36)**				**0.33 (0.32 to 0.34)**
					Both	0.03 (-0.07 to 0.12)				-0.01 (-0.10 to 0.07)
					Freshwater	**0.04 (0.02 to 0.06)**				-0.03 (-0.16 to 0.10)
TL	BM + diet + water	293	-714.3	9.4	a	**1.63 (1.61 to 1.65)**	0.97 (0.95 to 0.99)	-936.9	48.7	**1.72 (1.60 to 1.83)**
					b	**0.35 (0.34 to 0.36)**				**0.33 (0.32 to 0.34)**
					Omnivory	**-0.02 (-0.04 to -0.002)**				0.00 (-0.01 to 0.01)
					Herbivory	**-0.05 (-0.07 to -0.03)**				**-0.03 (-0.05 to -0.01)**
					Both	0.04 (-0.06 to 0.13)				-0.01 (-0.09 to 0.07)
					Freshwater	**0.05 (0.03 to 0.07)**				-0.03 (-0.15 to 0.10)
SL	BM	293	-694.6	29.1	a	**1.51 (1.50 to 1.53)**	0.98 (0.96 to 0.99)	-976.1	9.5	**1.63 (1.52 to 1.74)**
					b	**0.33 (0.32 to 0.35)**				**0.32 (0.32 to 0.34)**
SL	BM + diet	293	-701.3	22.4	a	**1.53 (1.52 to 1.55)**	0.97 (0.95 to 0.99)	-985.6	0.0	**1.63 (1.53 to 1.74)**
					b	**0.34 (0.33 to 0.35)**				**0.33 (0.32 to 0.34)**
					Omnivory	-0.02 (-0.04 to 0.00)				0.00 (-0.01 to 0.01)
					Herbivory	**-0.05 (-0.07 to -0.03)**				**-0.03 (-0.05 to -0.01)**
SL	BM + water	293	-706.7	17.0	a	**1.52 (1.50 to 1.53)**	0.98 (0.96 to 0.99)	-972.5	13.1	**1.64 (1.53 to 1.76)**
					b	**0.36 (0.34 to 0.37)**				**0.33 (0.32 to 0.34)**
					Both	0.01 (-0.08 to 0.11)				-0.01 (-0.09 to 0.06)
					Freshwater	**0.05 (0.03 to 0.07)**				-0.04 (-0.16 to 0.09)
SL	BM + diet + water	293	-723.7	0.0	a	**1.54 (1.52 to 1.56)**	0.98 (0.95 to 0.99)	-982.0	3.6	**1.65 (1.54 to 1.75)**
					b	**0.36 (0.35 to 0.37)**				**0.33 (0.32 to 0.34)**
					Omnivory	**-0.03 (-0.05 to -0.01)**				0.00 (-0.01 to 0.01)
					Herbivory	**-0.06 (-0.08 to -0.04)**				**-0.03 (-0.05 to -0.01)**
					Both	0.02 (-0.07 to 0.11)				-0.01 (-0.09 to 0.07)
					Freshwater	**0.06 (0.04 to 0.08)**				-0.04 (-0.15 to 0.08)

Faunivory used for comparison when dietary proxies used.

Table S2 ctd. Summary statistics for models assessing fish **body size** proxies (body mass (BM), standard length (SL) or total length (TL)) alone, or additionally with a **diet** proxy or/and **aquatic habitat** (freshwater or marine) according to log= a + b log (BM) + c (diet proxy) + d (water); (significant parameters in **bold**, almost significant parameters (0.051 – 0.10) in *italics*).

			**GLS**				**PGLS**			
**Model**		**n**	**AICc**	**ΔAIC**		**parameter (95%CI)**	**lambda (95%CI)**	**AICc**	**ΔAIC**	**parameter (95%CI)**
Only Lake Tanganyika´s cichlids data										
SL	TL	117	-583.4	-	a	**-0.08 (-0.10 to -0.06)**	0.54 (0.26 to 0.78)	-625.3	-	**-0.07 (-0.10 to -0.05)**
					b	**0.98 (0.97 to 1.00)**				**0.98 (0.96 to 1.00)**
TL	BM	117	-350.7	42.2	a	**1.62 (1.59 to 1.66)**	0.82 (0.62 to 0.94)	-453.3	0.0	**1.63 (1.59 to 1.67)**
					b	**0.33 (0.31 to 0.35)**				**0.33 (0.31 to 0.35)**
TL	BM + diet	117	-352.6	0.0	a	**1.65 (1.62 to 1.68)**	0.79 (0.58 to 0.94)	-451.3	37.8	**1.64 (1.60 to 1.68)**
					b	**0.34 (0.32 to 0.35)**				**0.33 (0.31 to 0.035)**
					Omnivory	*-0.02 (-0.04 to 0.001)*				-0.005 (-0.02 to 0.01)
SL	BM	117	-381.1	11.8	a	**1.53 (1.50 to 1.56)**	0.89 (0.70 to NA)	-489.1	0.0	**1.53 (1.49 to 1.57)**
					b	**0.33 (0.31 to 0.35)**				**0.33 (0.31 to 0.34)**
SL	BM + diet	117	-392.9	0.0	a	**1.56 (1.53 to 1.58)**	0.85 (0.63 to 0.99)	-488.5	0.6	**1.53 (1.50 to 1.57)**
					b	**0.24 (0.32 to 0.35)**				**0.33 (0.31 to 0.34)**
					Omnivory	*-0.02 (-0.03 to 0.002)*				0.002 (-0.01 to 0.02)
					Herbivory	**-0.06 (-0.08 to -0.04)**				-0.02 (-0.04 to 0.01)

Faunivory used for comparison when dietary proxies used.

Table S3 Summary statistics for models assessing **faunivore** fish **body size** proxies (body mass (BM), standard length (SL) or total length (TL)) alone, or additionally with a **diet** proxy or/and **aquatic habitat** (freshwater or marine) according to log= a + b log (BM) + c (diet proxy) + d (water); (significant parameters in **bold**, almost significant parameters (0.051 – 0.10) in *italics*).

			GLS				PGLS			
**Model**		**n**	**AICc**	**ΔAIC**		**parameter (95%CI)**	**lambda (95%CI)**	**AICc**	**ΔAIC**	**parameter (95%CI)**
All data										
SL	TL	181	-746.3	-	a	**-0.12 (-0.14 to -0.10)**	0.80 (0.56 to 0.92)	-800.0	-	**-0.09 (-0.14 to -0.04)**
					b	**1.02 (1.00 to 1.03)**				**1.01 (0.99 to 1.03)**
TL	BM	239	-452.0	33.7	a	**1.63 (1.60 to 1.65)**	0.98 (0.96 to 0.99)	-609.6	12.0	**1.68 (1.45 to 1.91)**
					b	**0.33 (0.31 to 0.34)**				**0.32 (0.30 to 0.33)**
TL	BM + diet	239	-485.7	0.0	a	**1.69 (1.66 to 1.72)**	0.98 (0.95 to 0.99)	-621.6	0.0	**1.71 (1.49 to 1.92)**
					b	**0.31 (0.30 to 0.33)**				**0.31 (0.29 to 0.32)**
					Both	**-0.05 (-0.09 to -0.02)**				**-0.03 (-0.07 to 0.00)**
					Invertivory	**-0.11 (-0.14 to -0.07)**				**-0.06 (-0.10 to -0.03)**
					Corallivory	**-0.19 (-0.25 to -0.14)**				**-0.11 (-0.17 to -0.05)**
TL	BM + water	239	-439.8	45.9	a	**1.63 (1.61 to 1.65)**	0.98 (0.96 to 0.99)	-608.2	13.4	**1.68 (1.46 to 1.91)**
					b	**0.34 (0.32 to 0.35)**				**0.32 (0.30 to 0.33)**
					Both	-0.02 (-0.10 to 0.06)				-0.06 (-0.13 to 0.01)
					Freshwater	0.02 (0.00 to 0.05)				0.02 (-0.10 to 0.14)
TL	BM + diet + water	239	-471.4	14.3	a	**1.68 (1.66 to 1.73)**	0.98 (0.95 to 0.99)	-619.8	1.8	**1.71 (1.49 to 1.92)**
					b	**0.31 (0.29 to 0.33)**				**0.31 (0.29 to 0.32)**
					Both	**-0.05 (-0.09 to -0.02)**				**-0.03 (-0.07 to 0.00)**
					Invertivory	**-0.11 (-0.15 to -0.07)**				**-0.06 (-0.09 to -0.03)**
					Corallivory	**-0.20 (-0.25 to -0.14)**				**-0.11 (-0.16 to -0.05)**
					Both	-0.04 (-0.11 to 0.03)				-0.05 (-0.12 to 0.02)
					Freshwater	-0.01 (-0.03 to 0.02)				0.01 (-0.11 to 0.13)
SL	BM	202	-455.9	44.4	a	**1.53 (1.51 to 1.55)**	0.97 (0.94 to 0.99)	-630.7	17.6	**1.62 (1.52 to 1.72)**
					b	**0.34 (0.32 to 0.35)**				**0.33 (0.32 to 0.35)**
SL	BM + diet	202	-500.3	0.0	a	**1.63 (1.60 to 1.66)**	0.96 (0.92 to 0.98)	-648.3	0.0	**1.65 (1.56 to 1.74)**
					b	**0.32 (0.31 to 0.34)**				**0.33 (0.31 to 0.34)**
					Both	**-0.11 (-0.14 to -0.07)**				**-0.06 (-0.09 to -0.03)**
					Invertivory	**-0.13 (-0.16 to -0.10)**				**-0.07 (-0.10 to -0.04)**
					Corallivory	**-0.19 (-0.24 to -0.14)**				**-0.10 (-0.15 to -0.05)**
SL	BM + water	202	-439.8	60.5	a	**1.54 (1.52 to 1.56)**	0.97 (0.94 to 0.99)	-628.3	20.0	**1.63 (1.53 to 1.73)**
					b	**0.36 (0.34 to 0.37)**				**0.33 (0.32 to 0.35)**
					Both	-0.01 (-0.08 to 0.05)				-0.06 (-0.09 to 0.02)
					Freshwater	**0.06 (0.03 to 0.08)**				-0.02 (-0.09 to 0.04)
SL	BM + diet + water	202	-489.6	10.7	a	**1.62 (1.60 to 1.65)**	0.96 (0.92 to 0.98)	-645.9	2.4	**1.66 (1.57 to 1.75)**
					b	**0.33 (0.32 to 0.35)**				**0.33 (0.31 to 0.34)**
					Both	**-0.10 (-0.14 to -0.07)**				**-0.06 (-0.09 to -0.03)**
					Invertivory	**-0.12 (-0.15 to -0.09)**				**-0.07 (-0.10 to -0.04)**
					Corallivory	**-0.17 (-0.22 to -0.12)**				**-0.10 (-0.15 to -0.05)**
					Both	-0.01 (-0.07 to 0.04)				-0.03 (-0.08 to 0.02)
					Freshwater	**0.03 (0.00 to 0.05)**				-0.02 (-0.09 to 0.04)

Faunivory used for comparison when dietary proxies used; marine used for comparison when aquatic habitat proxies are used.

Table S3 ctd Summary statistics for models assessing **faunivore** fish **body size** proxies (body mass (BM), standard length (SL) or total length (TL)) alone, or additionally with a **diet** proxy or/and **aquatic habitat** (freshwater or marine) according to log= a + b log (BM) + c (diet proxy) + d (water); (significant parameters in **bold**, almost significant parameters (0.051 – 0.10) in *italics*).

			**GLS**				**PGLS**			
**Model**		**n**	**AICc**	**ΔAIC**		**parameter (95%CI)**	**lambda (95%CI)**	**AICc**	**ΔAIC**	**parameter (95%CI)**
Consistent data (species for which all body size proxies are available										
SL	TL	180	-746.3	-	a	**0.12 (-0.14 to -0.10)**	0.80 (0.55 to 0.92)	-799.9	-	**-0.09 (-0.14 to -0.04)**
					b	**1.02 (1.00 to 1.03)**				**1.01 (0.99 to 1.03)**
TL	BM	180	-415.3	41.6	a	**1.63 (1.61 to 1.65)**	0.95 (0.90 to 0.98)	-553.6	20.0	**1.71 (1.60 to 1.82)**
					b	**0.34 (0.32 to 0.35)**				**0.33 (0.32 to 0.35)**
TL	BM + diet	180	-456.9	0.0	a	**1.71 (1.68 to 1.74)**	0.93 (0.87 to 0.97)	-567.0	6.6	**1.74 (1.63 to 1.84)**
					b	**0.32 (0.31 to 0.34)**				**0.32 (0.31 to 0.34)**
					Both	**-0.08 (-0.12 to -0.05)**				**-0.05 (-0.08 to -0.02)**
					Invertivory	**-0.11 (-0.14 to -0.08)**				**-0.06 (-0.09 to -0.03)**
					Corallivory	**-0.20 (-0.24 to -0.15)**				**-0.11 (-0.16 to -0.06)**
TL	BM + water	180	-416.6	40.3	a	**1.63 (1.61 to 1.65)**	0.95 (0.90 to 0.98)	-549.7	23.9	**1.71 (1.60 to 1.82)**
					b	**0.36 (0.34 to 0.13)**				**0.33 (0.32 to 0.35)**
					Both	-0.01 (-0.15 to 0.13)				-0.02 (-0.11 to 0.07)
					Freshwater	**0.05 (0.03 to 0.08)**				-0.01 (-0.16 to 0.14)
TL	BM + diet + water	180	-444.2	12.7	a	**1.71 (1.68 to 1.74)**	0.93 (0.87 to 0.97)	-563.1	10.5	**1.74 (1.63 to 1.84)**
					b	**0.33 (0.32 to 0.35)**				**0.32 (0.31 to 0.34)**
					Both	**-0.08 (-0.12 to -0.04)**				**-0.05 (-0.08 to -0.02)**
					Invertivory	**-0.10 (-0.14 to -0.07)**				**-0.06 (-0.09 to -0.03)**
					Corallivory	**-0.18 (-0.23 to -0.14)**				**-0.11 (0.16 to -0.06)**
					Both	0.002 (-0.12 to 0.12)				-0.02 (0.10 to 0.08)
					Freshwater	0.02 (-0.01 to 0.04)				-0.02 (-0.15 to 0.12)
SL	BM	180	-394.3	62.5	a	**1.54 (1.52 to 1.56)**	0.97 (0.94 to 0.99)	-558.7	14.9	**1.65 (1.53 to 1.77)**
					b	**0.34 (0.33 to 0.36)**				**0.34 (0.32 to 0.35)**
SL	BM + diet	180	-435.1	21.8	a	**1.64 (1.61 to 1.67)**	0.96 (0.92 to 0.98)	-573.6	0.0	**1.68 (1.57 to 1.79)**
					b	**0.33 (0.32 to 0.34)**				**0.33 (0.31 to 0.34)**
					Both	**-0.10 (-0.14 to -0.06)**				**-0.06 (-0.09 to -0.02)**
					Invertivory	**-0.13 (-0.16 to -0.10)**				**-0.07 (-0.10 to -0.04)**
					Corallivory	**-0.19 (-0.24 to -0.14)**				**-0.10 (-0.15 to -0.05)**
SL	BM + water	180	-402.5	54.4	a	**1.55 (1.52 to 1.57)**	0.97 (0.93 to 0.99)	-554.7	18.9	**1.65 (1.53 to 1.77)**
					b	**0.37 (0.35 to 0.38)**				**0.34 (0.32 to 0.35)**
					Both	-0.02 (-0.17 to 0.12)				-0.01 (-0.10 to 0.08)
					Freshwater	**0.07 (0.04 to 0.09)**				-0.004 (-0.16 to 0.15)
SL	BM + diet + water	180	-427.5	29.4	a	**1.64 (1.60 to 1.67)**	0.96 (0.92 to 0.98)	-569.6	4.0	**1.68 (1.57 to 1.79)**
					b	**0.34 (0.33 to 0.36)**				**0.33 (0.31 to 0.34)**
					Both	**-0.10 (-0.14 to -0.06)**				**-0.06 (-0.09 to -0.02)**
					Invertivory	**-0.12 (-0.15 to -0.09)**				**-0.07 (-0.10 to -0.04)**
					Corallivory	**-0.17 (-0.22 to -0.12)**				**-0.10 (-0.15 to -0.05)**
					Both	-0.01 (0.13 to 0.12)				-0.003 (-0.09 to 0.08)
					Freshwater	*0.03 (0.01 to 0.06)*				-0.01 (-0.15 to 0.14)

Faunivory used for comparison when dietary proxies used; marine used for comparison when aquatic habitat proxies are used.

Table S4 Summary statistics for models assessing **herbivore** fish **body size** proxies (body mass (BM), standard length (SL) or total length (TL)) alone, or additionally with a **diet** proxy or/and **aquatic habitat** (freshwater or marine) according to log= a + b log (BM) + c (diet proxy) + d (water); (significant parameters in **bold**, almost significant parameters (0.051 – 0.10) in *italics*).

			GLS				PGLS			
**Model**		**n**	**AICc**	**ΔAIC**		**parameter (95%CI)**	**lambda (95%CI)**	**AICc**	**ΔAIC**	**parameter (95%CI)**
All data										
SL	TL	62	-245.5	-	a	**-0.13 (-0.16 to -0.09)**	0.85 (0.55 to 0.96)	-287.5	-	**-0.06 (-0.12 to -0.01)**
					b	**1.02 (0.99 to 1.05)**				**0.97 (0.93 to 1.01)**
TL	BM	68	-207.2	0.0	a	**1.58 (1.56 to 1.60)**	0.90 (0.63 to 0.98)	-232.2	0.0	**1.59 (1.50 to 1.67)**
					b	**0.33 (0.32 to 0.35)**				**0.32 (0.30 to 0.35)**
TL	BM + diet	68	-190.1	17.1	a	**1.58 (1.56 to 1.60)**	0.92 (0.69 to 0.99)	-229.2	3.0	**1.58 (1.49 to 1.67)**
					b	**0.33 (0.32 to 0.35)**				**0.32 (0.30 to 0.35)**
					Both	0.00 (-0.02 to 0.03)				0.02 (-0.02 to 0.05)
					Detritivory	0.01 (-0.02 to 0.04)				0.01 (-0.02 to 0.03)
TL	BM + water	68	-201.2	6.0	a	**1.57 (1.56 to 1.59)**	0.86 (NA to 0.97)	-232.0	0.2	**1.57 (1.49 to 1.66)**
					b	**0.33 (0.32 to 0.35)**				**0.32 (0.30 to 0.34)**
					Both	0.10 (0.04 to 0.16)				*0.11 (0.00 to 0.22)*
					Freshwater	0.01 (-0.02 to 0.04)				0.00 (-0.09 to 0.09)
TL	BM + diet + water	68	-185.1	22.1	a	**1.58 (1.56 to 1.59)**	0.88 (NA to 0.98)	-228.4	3.8	**1.57 (1.48 to 1.66)**
					b	**0.33 (0.31 to 0.35)**				**0.32 (0.30 to 0.34)**
					Both	-0.01 (-0.04 to 0.02)				0.01 (-0.02 to 0.04)
					Detritivory	0.00 (-0.04 to 0.04)				0.01 (-0.02 to 0.04)
					Both	**0.11 (0.04 to 0.17)**				*0.10 (-0.01 to 0.22)*
					Freshwater	0.01 (-0.03 to 0.04)				0.00 (-0.10 to 0.10)
SL	BM	80	-222.2	0.0	a	**1.48 (1.46 to 1.50)**	0.85 (0.56 to 0.97)	-274.5	0.0	**1.52 (1.46 to 1.59)**
					b	**0.33 (0.31 to 0.34)**				**0.33 (0.32 to 0.35)**
SL	BM + diet	80	-205.9	16.3	a	**1.48 (1.46 to 1.50)**	0.82 (0.56 to 0.95)	-274.4	0.1	**1.53 (1.47 to 1.59)**
					b	**0.32 (0.31 to 0.34)**				**0.33 (0.31 to 0.35)**
					Both	-0.01 (-0.04 to 0.02)				*-0.03 (-0.05 to 0.0005)*
					Detritivory	-0.01 (-0.04 to 0.03)				0.00 (-0.03 to 0.03)
SL	BM + water	80	-212.4	9.8	a	**1.48 (1.46 to 1.50)**	0.84 (0.53 to 0.96)	-271.0	3.5	**1.51 (1.44 to 1.58)**
					b	**0.34 (0.32 to 0.36)**				**0.34 (0.32 to 0.35)**
					Both	0.01 (-0.03 to 0.05)				0.02 (-0.06 to 0.10)
					Freshwater	0.04 (0.01 to 0.07)				0.02 (-0.04 to 0.08)
SL	BM + diet + water	80	-201.6	20.6	a	**1.48 (1.56 to 1.59)**	0.79 (0.46 to 0.94)	-271.5	3.0	**1.51 (1.45 to 1.58)**
					b	**0.34 (0.32 to 0.35)**				**0.33 (0.31 to 0.35)**
					Both	*-0.03 (-0.05 to 0.004)*				**-0.03 (-0.06 to -0.002)**
					Detritivory	**-0.05 (-0.09 to -0.01)**				0.00 (-0.03 to 0.03)
					Both	0.02 (-0.02 to 0.07)				0.03 (-0.04 to 0.11)
					Freshwater	**0.06 (0.03 to 0.09)**				0.03 (-0.10 to 0.09)

Algivory used for comparison when dietary proxies used; marine used for comparison when dieatry proxies are used.

Table S4 ctd Summary statistics for models assessing **herbivore** fish **body size** proxies (body mass (BM), standard length (SL) or total length (TL)) alone, or additionally with a **diet** proxy or/and **aquatic habitat** (freshwater or marine) according to log= a + b log (BM) + c (diet proxy) + d (water); (significant parameters in **bold**, almost significant parameters (0.051 – 0.10) in *italics*).

			GLS				PGLS			
**Model**		**n**	**AICc**	**ΔAIC**		**parameter (95%CI)**	**lambda (95%CI)**	**AICc**	**ΔAIC**	**parameter (95%CI)**
Consistent data (species for which all body size proxies are available										
SL	TL	62	-246.5	-	a	**-0.13 (-0.16 to -0.09)**	0.85 (0.63 to 0.98)	-287.5	-	**-0.06 (-0.12 to -0.01)**
					b	**1.02 (0.99 to 1.05)**				**0.97 (0.93 to 1.01)**
TL	BM	62	-195.1	11.4	a	**1.58 (1.56 to 1.60)**	0.89 (0.63 to 0.98)	-223.8	24.0	**1.57 (1.53 to 1.62)**
					b	**0.33 (0.32 to 0.35)**				**0.33 (0.31 to 0.36)**
TL	BM + diet	62	-179.1	27.4	a	**1.58 (1.56 to 1.60)**	0.91 (0.67 to 0.98)	-221.1	26.7	**1.57 (1.52 to 1.62)**
					b	**0.34 (0.32 to 0.36)**				**0.33 (0.31 to 0.36)**
					Both	-0.005 (-0.03 to 0.02)				0.01 (-0.02 to 0.04)
					Detritivory	0.01 (0.00 to 0.00)				0.01 (-0.02 to 0.03)
TL	BM + water	62	-184.1	22.4	a	**1.58 (1.56 to 1.60)**	0.87 (0.56 to 0.97)	-221.9	25.9	**1.56 (1.51 to 1.61)**
					b	**0.34 (0.32 to 0.36)**				**0.33 (0.31 to 0.35)**
					Both	*0.08 (-0.004 to 0.16)*				0.09 (-0.04 to 0.23)
					Freshwater	0.01 (-0.01 to 0.04)				-0.01 (-0.12 to 0.11)
TL	BM + diet + water	62	-169.1	37.4	a	**1.58 (1.56 to 1.60)**	0.90 (0.59 to 0.98)	-218.9	28.9	**1.56 (1.51 to 1.61)**
					b	**0.33 (0.31 to 0.35)**				**0.33 (0.30 to 0.35)**
					Both	-0.01 (-0.05 to 0.02)				0.01 (-0.02 to 0.04)
					Detritivory	0.01 (-0.03 to 0.05)				0.01 (-0.02 to 0.04)
					Both	**0.09 (0.004 to 0.18)**				0.09 (-0.05 to 0.23)
					Freshwater	-0.01 (-0.05 to 0.04)				0.09 (-0.13 to 0.11)
SL	BM	62	-206.5	0.0	a	**1.49(1.47 to 1.50)**	0.94 (0.74 to 1.00)	-247.8	0.0	**1.47 (1.43 to 1.51)**
					b	**0.34 (0.33 to 0.36)**				**0.33 (0.31 to 0.35)**
SL	BM + diet	62	-199.8	6.7	a	**1.49 (1.47 to 1.51)**	0.93 (0.63 to 0.98)	-245.3	2.5	**1.47 (1.43 to 1.52)**
					b	**0.34 (0.33 to 0.36)**				**0.33 (0.31 to 0.35)**
					Both	**-0.03 (-0.05 to -0.01)**				-0.01 (-0.04 to 0.01)
					Detritivory	0.02 (-0.01 to 0.04)				0.01 (-0.01 to 0.03)
SL	BM + water	62	-201.3	5.2	a	**1.49 (1.47 to 1.50)**	0.93 (0.70 to 1.00)	-245.5	2.3	**1.46 (1.42 to 1.51)**
					b	**0.36 (0.34 to 0.37)**				**0.33 (0.31 to 0.35)**
					Both	0.06 (-0.01 to 0.13)				0.08 (-0.05 to 0.20)
					Freshwater	**0.04 (0.01 to 0.06)**				-0.004 (-0.11 to 0.10)
SL	BM + diet + water	62	-191.2	15.3	a	**1.49 (1.47 to 1.50)**	0.90 (0.52 to 0.99)	-243.7	4.1	**1.47 (1.43 to 1.51)**
					b	**0.34 (0.33 to 0.36)**				**0.32 (0.30 to 0.34)**
					Both	-0.04 (-0.06 to -0.01)				-0.02 (-0.04 to 0.01)
					Detritivory	0.003 (-0.03 to 0.04)				0.01 (-0.01 to 0.03)
					Both	**0.09 (0.01 to 0.16)**				0.09 (-0.03 to 0.20)
					Freshwater	0.01 (-0.02 to 0.05)				-0.02 (-0.12 to 0.09)

Algivory used for comparison when dietary proxies used; marine used for comparison when dieatry proxies are used.

Table S5 Summary statistics for allometric scaling for all **body size** proxies (body mass (BM), standard length (SL) or total length (TL)) as log(y) = a + b log (Significant parameters in **bold**).

Dependent	Model	n	GLS AICc	ΔAIC		parameter (95%CI)	PGLS lambda (95%CI)	AICc	ΔAIC	parameter (95%CI)
All species										
Total intest.	BM	468	-	-	a	**1.70 (1.65 to 1.76)**	0.97 (0.95 to 0.98)	-	-	**1.36 (0.64 to 2.07)**
					b	**0.32 (0.29 to 0.36)**				**0.37 (0.34 to 0.41)**
Total intest.	TL	375	-	-	a	**0.43 (0.26 to 0.60)**	0.98 (0.93 to 0.99)	-	-	-0.44 (-1.25 to 0.37)
					b	**0.72 (0.58 to 0.85)**				**1.05 (0.91 to 1.17)**
Total intest.	SL	371	-	-	a	**0.36 (0.22 to 0.50)**	0.97 (0.94 to 0.98)	-	-	-0.06 (-0.52 to 0.40)
					b	**0.90 (0.78 to 1.03)**				**1.06 (0.94 to 1.19)**
Consistent data (species for which all body size proxies are available)										
Total intest.	BM	293	146.5	0.0	a	**1.76 (1.70 to 1.83)**	0.94 (0.90 to 0.96)	-122.2	0.0	**1.70 (1.31 to 2.07)**
					b	**0.32 (0.27 to 0.36)**				**0.39 (0.34 to 0.44)**
Total intest.	TL	293	195.6	49.1	a	**0.42 (0.24 to 0.59)**	0.89 (0.92 to 0.94)	-82.0	40.0	-0.10 (-0.60 to 0.38)
					b	**0.79 (0.65 to 0.93)**				**1.04 (0.89 to 1.19)**
Total intest.	SL	293	198.3	51.8	a	**0.52 (0.35 to 0.68)**	0.95 (0.93 to 0.98)	-80.3	41.9	-0.03 (-0.52 to 0.45)
					b	**0.77 (0.63 to 0.91)**				**1.04 (0.89 to 1.20)**
Only Lake Tanganyika´s cichlids data										
Total intest.	BM	117	23.8	0.0	a	**2.16 (1.99 to 2.33)**	0.84 (0.68 to 0.93)	-95.5	0.0	**1.93 (1.74 to 2.12)**
					b	**0.53 (0.44 to 0.62)**				**0.44 (0.37 to 0.52)**
Total intest	TL	117	48.0	24.2	a	-0.24 (-0.54 to 0.05)	0.88 (0.75 to 0.96)	-87.9	7.6	-0.20 (-0.49 to 0.09)
					b	**1.39 (1.10 to 1.69)**				**1.29 (1.06 to 1.51)**
Total intest	SL	117	49.8	26.0	a	-0.10 (-0.38 to 0.17)	0.88 (0.75 to 0.96)	-86.6	8.9	-0.08 (-0.37 to 0.20)
					b	**1.38 (1.09 to 1.68)**				**1.30 (1.06 to 1.53)**

Table S6 Summary statistics for models assessing fish **intestine diameter and length** with body mass (BM); according to log(length) = a + b log (BM); (significant parameters in **bold**).

		GLS					PGLS			
**Model**		**n**	**AICc**	**ΔAIC**		**parameter (95%CI)**	**lambda (95%CI)**	**AICc**	**ΔAIC**	**parameter (95%CI)**
Total intest.	BM	166	97.6	290.1	a	**1.68 (1.60 to 1.76)**	0.99 (0.97 to NA)	-137.7	134.7	**1.56 (1.22 to 1.89)**
					b	**0.23 (0.16 to 0.30)**				**0.32 (0.27 to 0.37)**
Intest. Diameter	BM	166	-192.5	0.0	a	**-0.06 (-0.09 to -0.03)**	0.85 (0.67 to 0.95)	-272.4	0.0	**-0.16 (-0.32 to -0.01)**
					b	**0.34 (0.31 to 0.37)**				**0.31 (0.28 to 0.34)**

Table S7 Summary statistics for models assessing fish **total intestine length** with body size proxies (body mass (BM), standard length (SL) or total length (TL)) alone, or additionally with a **diet** proxy according to log(length) = a + b log (BS) + c (diet proxy); (significant parameters in **bold**).

			GLS				PGLS			
**Dependent**	**Model**	**n**	**AICc**	**ΔAIC**		**parameter (95%CI)**	**lambda (95%CI)**	**AICc**	**ΔAIC**	**parameter (95% CI)**
All species										
Total intest.	BM	468	318.5	157.2	a	**1.70 (1.65 to 1.76)**	0.97 (0.95 to 0.98)	-76.6	114.1	**1.36 (0.64 to 2.07)**
					b	**0.32 (0.29 to 0.36)**				**0.37 (0.34 to 0.41)**
Total intest.	BM + diet	468	161.3	0.0	a	**1.52 (1.47 to 1.58)**	0.95 (0.93 to 0.97)	-190.7	0.0	**1.30 (0.74 to 1.88)**
					b	**0.29 (0.26 to 0.33)**				**0.35 (0.32 to 0.39)**
					Omnivory	**0.18 (0.12 to 0.25)**				**0.12 (0.07 to 0.16)**
					Herbivory	**0.47 (0.43 to 0.54)**				**0.34 (0.28 to 0.40)**
Total intest.	TL	375	294.8	130.2	a	**0.43 (0.26 to 0.60)**	0.98 (0.93 to 0.99)	-67.2	93.4	-0.44 (-1.25 to 0.37)
					b	**0.72 (0.58 to 0.85)**				**1.05 (0.91 to to 1.17)**
Total intest.	TL + diet	375	164.6	0.0	a	**0.36 (0.22 to 0.50)**	0.96 (0.95 to 0.98)	-160.6	0.0	-0.40 (-1.06 to 0.25)
					b	**0.66 (0.56 to 0.78)**				**1.00 (0.89 to 1.13)**
					Omnivory	**0.21 (0.13 to 0.29)**				**0.12 (0.07 to 0.17)**
					Herbivory	**0.52 (0.44 to 0.60)**				**0.35 (0.28 to 0.42)**
Total intest.	SL	371	267.8	126.6	a	**0.36 (0.22 to 0.50)**	0.97 (0.94 to 0.98)	-78.6	105.9	-0.06 (-0.52 to 0.40)
					b	**0.90 (0.78 to 1.03)**				**1.06 (0.94 to 1.19)**
Total intest.	SL + diet	371	141.2	0.0	a	**0.28 (0.16 to 0.41)**	0.93 (0.90 to 0.96)	-184.5	0.0	-0.10 (-0.47 to 0.25)
					b	**0.83 (0.73 to 0.94)**				**1.01 (0.91 to 1.12)**
					Omnivory	**0.19 (0.12 to 0.27)**				**0.14 (0.10 to 0.19)**
					Herbivory	**0.48 (0.41 to 0.55)**				**0.36 (0.30 to 0.42)**
Consistent data (species for which all body size proxies are available)										
Total intest.	BM	293	146.5	103.3	a	**1.76 (1.70 to 1.83)**	0.94 (0.90 to 0.96)	-122.2	76.1	**1.70 (1.31 to 2.07)**
					b	**0.32 (0.27 to 0.36)**				**0.39 (0.34 to 0.44)**
Total intest.	BM + diet	293	43.2	0.0	a	**1.59 (1.53 to 1.65)**	0.89 (0.83 to 0.94)	-198.3	0.0	**1.61 (1.32 to 1.90)**
					b	**0.28 (0.24 to 0.32)**				**0.36 (0.32 to 0.41)**
					Omnivory	**0.24 (0.16 to 0.32)**				**0.15 (0.10 to 0.20)**
					Herbivory	**0.42 (0.34 to 0.49)**				**0.31 (0.25 to 0.38)**
Total intest.	TL	293	195.6	152.4	a	**0.42 (0.24 to 0.59)**	0.89 (0.92 to 0.94)	-82.0	116.3	-0.10 (-0.60 to 0.38)
					b	**0.79 (0.65 to 0.93)**				**1.04 (0.89 to 1.19)**
Total intest.	TL + diet	293	87.9	44.7	a	**0.35 (0.21 to 0.50)**	0.92 (0.87 to 0.96)	-162.0	38.0	-0.09 (-0.48 to 0.30)
					b	**0.73 (0.61 to 0.85)**				**0.98 (0.85 to 1.11)**
					Omnivory	**0.26 (0.17 to 0.34)**				**0.15 (0.09 to 0.20)**
					Herbivory	**0.47 (0.39 to 0.55)**				**0.35 (0.28 to 0.42)**
Total intest.	SL	293	198.3	155.1	a	**0.52 (0.35 to 0.68)**	0.95 (0.93 to 0.98)	-80.3	118.0	-0.03 (-0.52 to 0.45)
					b	**0.77 (0.63 to 0.91)**				**1.04 (0.89 to 1.19)**
Total intest.	SL + diet	293	88.1	44.9	a	**0.43 (0.30 to 0.57)**	0.92 (0.87 to 0.96)	-161.0	37.3	-0.01 (-0.40 to 0.36)
					b	**0.72 (0.60 to 0.83)**				**0.98 (0.85 to 1.11)**
					Omnivory	**0.25 (0.17 to 0.34)**				**0.15 (0.10 to 0.20)**
					Herbivory	**0.47 (0.40 to 0.55)**				**0.35 (0.28 to 0.42)**

Faunivory used for comparison when dietary proxies used.

Table S7 ctd Summary statistics for models assessing fish **total intestine length** with body size proxies (body mass (BM), standard length (SL) or total length (TL)) alone, or additionally with a **diet** proxy according to log(length) = a + b log (BS) + c (diet proxy); (significant parameters in **bold**); (almost significant parameters (0.051 – 0.10) in *italics*).

			GLS				PGLS			
**Dependent**	**Model**	**n**	**AICc**	**ΔAIC**		**parameter (95%CI)**	**lambda (95%CI)**	**AICc**	**ΔAIC**	**parameter (95% CI)**
Only Lake Tanganyika´s cichlids data										
Total intest.	BM	117	23.8	139.9	a	**2.17 (1.99 to 2.34)**	0.84 (0.68 to 0.93)	-95.5	164.7	**1.93 (1.75 to 2.13)**
					b	**0.54 (0.45 to 0.63)**				**0.45 (0.37 to 0.52)**
Total intest.	BM + diet	117	-116.1	0.0	a	**1.87(1.77 to 1.96)**	0.57 (0.28 to 0.82)	-164.7	0.0	**1.84 (1.72 to 1.96)**
					b	**0.46 (0.41 to 0.51)**				**0.43 (0.38 to 0.48)**
					Omnivory	**0.24 (0.18 to 0.30)**				**0.18 (0.12 to 0.24)**
					Herbivory	**0.59 (0.52 to 0.65)**				**0.46 (0.38 to 0.54)**
Total intest	TL	117	48.0	164.1	a	-0.24 (-0.54 to 0.06)	0.89 (0.75 to 0.96)	-87.9	76.8	-0.20 (-0.50 to 0.10)
					b	**1.40 (1.10 to 1.69)**				**1.29 (1.07 to 1.51)**
Total intest.	TL + diet	117	-106.2	9.9	a	**-0.30 (-0.45 to -0.16)**	0.72 (0.43 to 0.91)	-157.3	7.4	-0.23 (-0.44 to -0.03)
					b	**1.29 (1.15 to 1.43)**				**1.26 (1.10 to 1.42)**
					Omnivory	**0.26 (0.20 to 0.33)**				**0.18 (0.12 to 0.24)**
					Herbivory	**0.66 (0.59 to 0.73)**				**0.48 (0.39 to 0.57)**
Total intest	SL	117	49.8	165.9	a	-0.10 (-0.38 to 0.17)	0.88 (0.75 to 0.96)	-86.6	78.1	-0.09 (-0.37 to 0.20)
					b	**1.39 (1.09 to 1.69)**				**1.30 (1.07 to 1.53)**
Total intest.	SL + diet	117	-103.4	12.7	a	*-0.18 (-0.32 to -0.05)*	0.71 (0.42 to 0.89)	-153.7	11.0	-0.12 (-0.31 to 0.08)
					b	**1.29 (1.15 to 1.44)**				**1.26 (1.09 to 1.43)**
					Omnivory	**0.26 (0.19 to 0.32)**				**0.17 (0.11 to 0.24)**
					Herbivory	**0.67 (0.60 to 0.74)**				**0.48 (0.39 to 0.57)**

Faunivory used for comparison when dietary proxies used.

Table S8 Summary statistics for models assessing **faunivore** fish **total intestine length** with body size proxies (body mass (BM), standard length (SL) or total length (TL)) alone, or additionally with a **diet** proxy and according to log(length) = a + b log (BS) + c (diet); (significant parameters in **bold**); (almost significant parameters (0.051 – 0.10) in *italics*).

Model		n	GLS AICc	ΔAIC		parameter (95%CI)	PGLS lambda (95%CI)	AICc	ΔAIC	parameter (95%CI)
All data										
Total intest.	BM	264	67.7	83.8	a	**1.52 (1.46 to 1.58)**	0.98 (0.96 to 0.99)	-207.5	14.5	**1.28 (0.75 to 1.81)**
					b	**0.29 (0.24 to 0.33)**				**0.36 (0.33 to 0.40)**
Total intest.	BM + faunivory	264	-16.1	0.0	a	**1.46 (1.37 to 1.54)**	0.97 (0.95 to 0.99)	-222.0	0.0	**1.25 (0.75 to 1.75)**
					b	**0.32 (0.29 to 0.36)**				**0.39 (0.35 to 0.42)**
					Both	-0.03 (-0.13 to 0.07)				0.03 (-0.04 to 0.11)
					Invertebrates	**0.15 (0.06 to 0.25)**				**0.11 (0.04 to 0.19)**
					Corallivory	**0.70 (0.55 to 0.85)**				**0.29 (0.15 to 0.42)**
Total intest.	TL	239	107.4	86.8	a	**0.40 (0.23 to 0.58)**	0.98 (0.96 to 0.99)	-149.5	24.0	-0.43 (-1.05 to 0.18)
					b	**0.64 (0.50 to 0.78)**				**1.01 (0.89 to 1.13)**
Total intest.	TL + faunivory	239	20.8	0.0	a	**-0.08 (-0.19 to 0.13)**	0.98 (0.95 to 0.99)	-173.5	0.0	**-0.69 (-1.27 to -0.12)**
					b	**0.88 (0.75 to 1.01)**				**1.13 (1.00 to 1.25)**
					Both	0.01 (-0.09 to 0.12)				*0.07 (-0.01 to 0.16)*
					Invertebrates	**0.24 (0.13 to 0.34)**				**0.18 (0.09 to 0.26)**
					Corallivory	**0.85 (0.68 to 1.02)**				**0.409 (0.25 to 0.55)**
Total intest.	SL	202	69.3	55.7	a	**0.35 (0.20 to 0.51)**	0.95 (0.91 to 0.98)	-155.7	11.5	-0.09 (-0.44 to 0.25)
					b	**0.78 (0.64 to 0.92)**				**0.99 (0.87 to 1.11)**
Total intest.	SL + faunivory	202	13.6	0.0	a	-0.01 (-0.21 to 0.20)	0.94 (0.90 to 0.97)	-167.2	0.0	-0.27 (-0.61 to 0.08)
					b	**0.92 (0.79 to 1.04)**				**1.06 (0.93 to 1.19)**
					Both	*0.12 (-0.01 to 0.25)*				**0.12 (0.02 to 0.22)**
					Invertebrates	**0.22 (0.10 to 0.34)**				**0.14 (0.05 to 0.24)**
					Corallivory	**0.80 (0.62 to 0.98)**				**0.34 (0.18 to 0.50)**
Consistent data (species for which all body size proxies are available)										
Total intest.	BM	180	34.0	43.3	a	**1.61 (1.54 to 1.68)**	0.95 (0.91 to 0.98)	-172.1	8.5	**1.53 (1.21 to 1.86)**
					b	**0.30 (0.25 to 0.35)**				**0.37 (0.33 to 0.41)**
Total intest.	BM + faunivory	180	-9.3	0.0	a	**1.49 (1.38 to 1.59)**	0.94 (0.89 to 0.97)	-180.6	0.0	**1.48 (1.17 to 1.79)**
					b	**0.32 (0.28 to 0.36)**				**0.39 (0.35 to 0.43)**
					Both	0.06 (-0.07 to 0.19)				0.06 (-0.03 to 0.15)
					Invertebrates	*0.15 (0.03 to 0.26)*				**0.11 (0.04 to 0.20)**
					Corallivory	**0.65 (0.49 to 0.82)**				**0.28 (0.13 to 0.42)**
Total intest.	TL	180	70.9	80.2	a	**0.35 (0.17 to 0.54)**	0.96 (0.92 to 0.98)	-138.8	41.8	-0.21 (-0.63 to 0.21)
					b	**0.73 (0.58 to 0.88)**				**1.01 (0.88 to 1.14)**
Total intest.	TL + faunivory	180	19.2	28.5	a	-0.05 (-0.28 to 0.18)	0.95 (0.91 to 0.98)	-151.9	28.7	**-0.42 (-0.84 to -0.01)**
					b	**0.88 (0.75 to 1.02)**				**1.09 (0.95 to 1.23)**
					Both	*0.13 (-0.02 to 0.27)*				**0.11 (0.01 to 0.21)**
					Invertebrates	**0.22 (0.10 to 0.35)**				**0.16 (0.06 to 0.26)**
					Corallivory	**0.81 (0.62 to 0.99)**				**0.36 (0.20 to 0.52)**

Piscivory used for comparison when dietary proxies used.

Table S8 *ctd* Summary statistics for models assessing **faunivore** fish **total intestine length** with body size proxies (body mass (BM), standard length (SL) or total length (TL)) alone, or additionally with a **diet** proxy and according to log(length) = a + b log (BS) + c (diet); (significant parameters in **bold**); (almost significant parameters (0.051 – 0.10) in *italics*).

Model		n	GLS AICc	ΔAIC		parameter (95%CI)	PGLS lambda (95%CI)	AICc	ΔAIC	parameter (95%CI)
Consistent data (species for which all body size proxies are available)										
Total intest.	SL	180	69.4	78.7	a	**0.43 (0.27 to 0.60)**	0.96 (0.92 to 0.98)	-133.9	46.7	-0.10 (-0.52 to 0.31)
					b	**0.72 (0.57 to 0.87)**				**0.98 (0.85 to 1.11)**
Total intest.	SL + faunivory	180	20.1	29.4	a	0.04 (-0.17 to 0.26)	0.94 (0.90 to 0.98)	-145.9	34.7	-0.31 (-0.73 to 0.10)
					b	**0.87 (0.73 to 1.00)**				**1.06 (0.92 to 1.21)**
					Both	0.14 (0.00 to 0.29)				**0.12 (0.02 to 0.22)**
					Invertebrates	**0.24 (0.11 to 0.36)**				**0.16 (0.06 to 0.26)**
					Corallivory	**0.80 (0.62 to 0.99)**				**0.36 (0.19 to 0.52)**

Piscivory used for comparison when dietary proxies used.

Table S9 Summary statistics for models assessing **herbivore** fish **total intestine length** with body size proxies (body mass (BM), standard length (SL) or total length (TL)) alone, or additionally with a **diet** proxy according to log(length) = a + b log (BS) + c (diet); (significant parameters in **bold**).

**Model**		**n**	**GLS AICc**	**ΔAIC**		**parameter (95%CI)**	**PGLS lambda (95%CI)**	**AICc**	**ΔAIC**	**parameter (95%CI)**
All data										
Total intest.	BM	88	18.5	0.0	a	**1.92 (1.84 to 2.01)**	0.79 (0.54 to 0.93)	-19.2	0.0	**2.03 (1.75 to 2.31)**
					b	**0.19 (0.12 to 0.26)**				**0.33 (0.25 to 0.42)**
Total intest.	BM + herbivory	88	21.2	2.7	a	**1.90 (1.81 to 1.99)**	0.76 (0.46 to 0.92)	-15.8	3.4	**2.02 (1.75 to 2.29)**
					b	**0.23 (0.15 to 0.30)**				**0.33 (0.24 to 0.42)**
					Both	0.04 (-0.08 to 0.16)				-0.01 (-0.14 to 0.12)
					Detritivory	**0.21 (0.07 to 0.35)**				0.05 (-0.08 to 0.19)
Total intest.	TL	68	-0.1	0.0	a	**1.14 (0.86 to 1.42)**	0.55 (0.18 to 0.85)	-20.7	0.0	**0.61 (0.16 to 1.06)**
					b	**0.47 (0.25 to 0.68)**				**0.83 (0.54 to 1.12)**
Total intest.	TL + herbivory	68	5.3	5.4	a	**1.02 (0.70 to 1.35)**	0.47 (0.06 to 0.82)	-17.6	3.1	**0.69 (0.23 to 1.14)**
					b	**0.54 (0.31 to 0.77)**				**0.78 (0.50 to 1.07)**
					Both	-0.04 (-0.17 to 0.09)				-0.03 (-0.16 to 0.11)
					Detritivory	**0.14 (0.00 to 0.28)**				0.06 (-0.09 to 0.21)
Total intest.	SL	80	13.1	0.0	a	**1.09 (0.83 to 1.35)**	0.83 (0.60 to 0.95)	-22.3	0.0	**0.45 (0.04 to 0.,86)**
					b	**0.57 (0.35 to 0.79)**				**1.08 (0.82 to 1.34)**
Total intest.	SL + herbivory	80	13.3	0.2	a	**0.81 (0.50 to 1.13)**	0.79 (0.50 to 0.94)	-19.8	2.5	**0.45 (0.03 to 0.87)**
					b	**0.75 (0.51 to 0.98)**				**1.06 (0.79 to 1.33)**
					Both	0.06 (-0.06 to 0.18)				0.01 (-0.12 to 0.15)
					Detritivory	**0.25 (0.10 to 0.40)**				0.09 (-0.05 to 0.23)
Consistent data (species for which all body size proxies are available)										
Total intest.	BM	62	-0.6	2.9	a	**1.89 (1.80 to 1.98)**	0.45 (0.14 to 0.77)	-23.8	0.1	**1.96 (1.82 to 2.10)**
					b	**0.19 (0.11 to 0.28)**				**0.31 (0.20 to 0.41)**
Total intest.	BM + herbivory	62	1.5	5.0	a	**1.90 (1.81 to 1.99)**	0.32 (0.01 to 0.70)	-22.6	1.3	**1.94 (1.81 to 2.07)**
					b	**0.20 (0.11 to 0.28)**				**0.28 (0.18 to 0.37)**
					Both	-0.06 (-0.19 to 0.08)				-0.05 (-0.18 to 0.09)
					Detritivory	**0.17 (0.03 to 0.31)**				0.12 (-0.04 to 0.27)
Total intest.	TL	62	-3.5	0.0	a	**1.14 (0.86 to 1.42)**	0.44 (0.12 to 0.79)	-23.9	0.0	**0.56 (0.16 to 0.96)**
					b	**0.48 (0.26 to 0.69)**				**0.88 (0.59 to 1.17)**
Total intest.	TL + herbivory	62	-0.7	2.8	a	**1.00 (0.66 to 1.34)**	0.32 (0.01 to 0.73)	-22.5	1.4	**0.69 (0.29 to 1.09)**
					b	**0.57 (0.33 to 0.81)**				**0.79 (0.50 to 1.08)**
					Both	-0.06 (-0.19 to 0.08)				-0.05 (-0.19 to 0.09)
					Detritivory	**0.16 (0.02 to 0.30)**				0.11 (-0.05 to 0.26)
Total intest.	SL	62	-2.3	1.2	a	**1.22 (0.97 to 1.48)**	0.47 (0.13 to 0.81)	-22.5	1.4	**0.66 (0.28 to 1.04)**
					b	**0.45 (0.23 to 0.66)**				**0.87 (0.57 to 1.17)**
Total intest.	SL + herbivory	62	1.6	5.1	a	**1.11 (0.79 to 1.43)**	0.36 (0.02 to 0.76)	-20.5	3.4	**0.77 (0.38 to 1.16)**
					b	**0.53 (0.28 to 0.77)**				**0.78 (0.49 to 1.08)**
					Both	-0.05 (-0.19 to 0.09)				-0.03 (-0.17 to 0.11)
					Detritivory	**0.15 (0.01 to 0.29)**				0.11 (-0.05 to 0.26)

Algivory used for comparison when dietary proxies used.

Table S10 Summary statistics for models assessing fish **total intestine length** with body size proxies (body mass (BM), standard length (SL) or total length (TL)) alone, or additionally with **aquatic habitat** (freshwater or marine) according to log(length) = a + b log (BS) + c (water); (significant parameters in **bold**); (almost significant parameters (0.051 – 0.10) in *italics*).

			GLS				PGLS			
**Dependent**	**Model**	**n**	**AICc**	**ΔAIC**		**parameter (95%CI)**	**lambda (95%CI)**	**AICc**	**ΔAIC**	**parameter (95%CI)**
All data										
Total intest.	BM	468	318.5	2.4	a	**1.70 (1.65 to 1.76)**	0.97 (0.95 to 0.98)	-76.6	5.2	**1.36 (0.64 to 2.07)**
					b	**0.32 (0.29 to 0.36)**				**0.37 (0.34 to 0.41)**
Total intest.	BM + water	468	316.1	0.0	a	**1.69 (1.63 to 1.75)**	0.97 (0.56 to 0.98)	-81.8	0.0	**1.34 (0.62 to 2.05)**
					b	**0.36 (0.31 to 0.40)**				**0.37 (0.33 to 0.41)**
					Both	-0.07 (-0.22 to 0.06)				-0.02 (-0.16 to 0.12)
					Fresh	**0.12 (0.06 to 0.19)**				**0.19 (0.02 to 0.38)**
Total intest.	TL	375	294.8	0.0	a	**0.43 (0.26 to 0.60)**	0.98 (0.93 to 0.99)	-67.2	0.5	-0.44 (-1.25 to 0.37)
					b	**0.72 (0.58 to 0.85)**				**1.05 (0.91 to to 1.17)**
Total intest.	TL + water	375	303.1	8.3	a	**0.35 (0.14 to 0.55)**	0.97 (0.96 to 0.98)	-67.7	0.0	-0.51 (-1.31 to 0.30)
					b	**0.78 (0.62 to 0.93)**				**1.04 (0.91 to 1.18)**
					Both	-0.08 (-0.33 to 0.17)				0.06 (-0.17 to 0.29)
					Fresh	0.06 (-0.02 to 0.14)				**0.37 (0.02 to 0.71)**
Total intest.	SL	371	267.8	0.0	a	**0.36 (0.22 to 0.50)**	0.97 (0.94 to 0.98)	-78.6	0.4	-0.06 (-0.52 to 0.40)
					b	**0.90 (0.78 to 1.03)**				**1.06 (0.94 to 1.19)**
Total intest.	SL + water	371	279.4	11.6	a	**0.44 (0.26 to 0.62)**	0.96 (0.94 to 0.98)	-79.0	0.0	-0.11 (-0.58 to 0.36)
					b	**0.77 (0.71 to 0.99)**				**1.05 (0.93 to 1.18)**
					Both	-0.09 (-0.28 to 0.10)				-0.01 (-0.20 to 0.18)
					Fresh	-0.05 (-0.14 to 0.02)				0.17 (-0.05 to 0.38)
Consistent data (species for which all body size proxies are available)										
Total intest.	BM	293	146.5	0.0	a	**1.76 (1.70 to 1.83)**	0.94 (0.90 to 0.96)	-122.2	0.0	**1.70 (1.31 to 2.07)**
					b	**0.32 (0.27 to 0.36)**				**0.39 (0.34 to 0.44)**
Total intest.	BM + water	293	155.5	9.0	a	**1.77 (1.70 to 1.83)**	0.94 (0.90 to 0.96)	-120.2	2.0	**1.60 (1.19 to 2.00)**
					b	**0.33 (0.28 to 0.39)**				**0.39 (0.35 to 0.44)**
					Both	-0.05 (-0.47 to 0.38)				0.01 (-0.31 to 0.33)
					Fresh	0.03 (-0.06 to 0.12)				0.31 (-0.13 to 0.75)
Total intest.	TL	293	195.6	49.1	a	**0.42 (0.24 to 0.59)**	0.89 (0.92 to 0.94)	-82.0	40.2	-0.10 (-0.60 to 0.38)
					b	**0.79 (0.65 to 0.93)**				**1.04 (0.89 to 1.19)**
Total intest.	TL + water	293	202.3	55.8	a	**0.53 (0.31 to 0.76)**	0.96 (0.93 to 0.98)	-79.5	42.7	-0.22 (-0.74 to 0.30)
					b	**0.72 (0.56 to 0.89)**				**1.05 (0.89 to 1.20)**
					Both	-0.03 (-0.49 to 0.43)				0.02 (-0.33 to 0.37)
					Fresh	-0.07 (-0.17 to 0.02)				0.32 (-0.19 to 0.83)
Total intest.	SL	293	198.3	51.8	a	**0.52 (0.35 to 0.68)**	0.95 (0.93 to 0.98)	-80.3	41.9	-0.03 (-0.52 to 0.45)
					b	**0.77 (0.63 to 0.91)**				**1.04 (0.89 to 1.19)**
Total intest.	SL + water	293	204.2	57.7	a	**0.64 (0.44 to 0.84)**	0.96 (0.93 to 0.98)	-77.9	44.3	-0.15 (-0.67 to 0.37)
					b	**0.70 (0.53 to 0.86)**				**1.05 (0.90 to 1.20)**
					Both	-0.02 (-0.48 to 0.44)				0.02 (-0.33 to 0.38)
					Fresh	*-0.09 (-0.18 to 0.004)*				0.33 (-0.18 to 0.84)

Marine used for comparison when aquatic habitat proxies are used.

Table S11 Summary statistics for models assessing fish **total intestine length** with body size proxies (body mass (BM), standard length (SL) or total length (TL)) alone, or additionally with a **diet** proxy and **aquatic habitat** (freshwater or marine) according to log(length) = a + b log (BS) + c (diet) + d (water); (significant parameters in **bold**).

			**GLS**				**PGLS**			
**Dependent**	**Model**	**n**	**AICc**	**ΔAIC**		**parameter (95%CI)**	**lambda (95%CI)**	**AICc**	**ΔAIC**	**parameter (95%CI)**
All data										
Total intest.	BM	468	318.5	157.2	a	**1.70 (1.65 to 1.76)**	0.97 (0.95 to 0.98)	-76.6	117.1	**1.36 (0.64 to 2.07)**
					b	**0.32 (0.29 to 0.36)**				**0.37 (0.34 to 0.41)**
Total intest.	BM + diet	468	161.3	0.0	a	**1.52 (1.47 to 1.58)**	0.95 (0.93 to 0.97)	-190.7	3.0	**1.30 (0.74 to 1.88)**
					b	**0.29 (0.26 to 0.33)**				**0.35 (0.32 to 0.39)**
					Omnivory	**0.18 (0.12 to 0.25)**				**0.12 (0.07 to 0.16)**
					Herbivory	**0.47 (0.43 to 0.54)**				**0.34 (0.28 to 0.40)**
Total intest.	BM + water	468	316.1	154.8	a	**1.69 (1.63 to 1.75)**	0.97 (0.56 to 0.98)	-81.8	111.9	**1.34 (0.62 to 2.05)**
					b	**0.36 (0.31 to 0.40)**				**0.37 (0.33 to 0.41)**
					Both	-0.07 (-0.22 to 0.06)				-0.02 (-0.16 to 0.12)
					Fresh	**0.12 (0.06 to 0.19)**				**0.19 (0.02 to 0.38)**
Total intest.	BM + diet + water	468	163.0	1.7	a	**1.54 (1.49 to 1.59)**	0.95 (0.94 to 0.98)	-193.7	0.0	**1.30 (0.74 to 1.87)**
					b	**0.31 (0.28 to 0.35)**				**0.35 (0.32 to 0.39)**
					Omnivory	**0.17 (0.10 to 0.23)**				**0.11 (0.07 to 0.16)**
					Herbivory	**0.47 (0.40 to 0.54)**				**0.34 (0.28 to 0.40)**
					Both	**-0.13 (-0.26 to -0.02)**				-0.04 (-0.16 to 0.07)
					Fresh	**0.06 (0.003 to 0.11)**				**0.14 (0.001 to 0.29)**
Total intest.	TL	375	294.8	130.2	a	**0.43 (0.26 to 0.60)**	0.98 (0.93 to 0.99)	-67.2	93.7	-0.44 (-1.25 to 0.37)
					b	**0.72 (0.58 to 0.85)**				**1.05 (0.91 to to 1.17)**
Total intest.	TL + diet	375	164.6	0.0	a	**0.36 (0.22 to 0.50)**	0.96 (0.95 to 0.98)	-160.6	0.3	-0.40 (-1.06 to 0.25)
					b	**0.66 (0.56 to 0.78)**				**1.00 (0.89 to 1.13)**
					Omnivory	**0.21 (0.13 to 0.29)**				**0.12 (0.07 to 0.17)**
					Herbivory	**0.52 (0.44 to 0.60)**				**0.35 (0.28 to 0.42)**
Total intest.	TL + water	375	303.1	138.5	a	**0.35 (0.14 to 0.55)**	0.97 (0.96 to 0.98)	-67.7	93.2	-0.51 (-1.31 to 0.30)
					b	**0.78 (0.62 to 0.93)**				**1.04 (0.91 to 1.18)**
					Both	-0.08 (-0.33 to 0.17)				0.06 (-0.17 to 0.29)
					Fresh	0.06 (-0.02 to 0.14)				**0.37 (0.02 to 0.71)**
Total intest.	TL + diet + water	375	173.9	9.3	a	**0.33 (0.17 to 0.50)**	0.96 (0.94 to 0.97)	-160.9	0.0	-0.46 (-1.11 to 0.20)
					b	**0.69 (0.57 to 0.82)**				**1.01 (0.89 to 1.12)**
					Omnivory	**0.21 (0.13 to 0.29)**				**0.12 (0.07 to 0.17)**
					Herbivory	**0.52 (0.44 to 0.60)**				**0.35 (0.29 to 0.42)**
					Both	-0.10 (-0.31 to 0.11)				0.02 (-0.18 to 0.22)
					Fresh	0.02 (-0.05 to 0.09)				**0.30 (0.02 to 0.59)**

Faunivory used for comparison when dietary proxies used; marine used for comparison when aquatic habitat proxies are used.

Table S11 ***ctd*** Summary statistics for models assessing fish **total intestine length** with body size proxies (body mass (BM), standard length (SL) or total length (TL)) alone, or additionally with a **diet** and **aquatic habitat** (freshwater or marine) according to log(length) = a + b log (BS) + c (diet) + d (water); (significant parameters in **bold**).

			GLS				PGLS			
**Model**		**n**	**AICc**	**ΔAIC**		**parameter (95%CI)**	**lambda (95%CI)**	**AICc**	**ΔAIC**	**parameter (95%CI)**
All data										
Total intest.	SL	371	267.8	126.6	a	**0.36 (0.22 to 0.50)**	0.97 (0.94 to 0.98)	-78.6	105.9	-0.06 (-0.52 to 0.40)
					b	**0.90 (0.78 to 1.03)**				**1.06 (0.94 to 1.19)**
Total intest.	SL + diet	371	141.2	0.0	a	**0.28 (0.16 to 0.41)**	0.93 (0.90 to 0.96)	-184.5	0.0	-0.10 (-0.47 to 0.25)
					b	**0.83 (0.73 to 0.94)**				**1.01 (0.91 to 1.12)**
					Omnivory	**0.19 (0.12 to 0.27)**				**0.14 (0.10 to 0.19)**
					Herbivory	**0.48 (0.41 to 0.55)**				**0.36 (0.30 to 0.42)**
Total intest.	SL + water	371	279.4	138.2	a	**0.44 (0.26 to 0.62)**	0.96 (0.94 to 0.98)	-79.0	105.5	-0.11 (-0.58 to 0.36)
					b	**0.77 (0.71 to 0.99)**				**1.05 (0.93 to 1.18)**
					Both	-0.09 (-0.28 to 0.10)				-0.01 (-0.20 to 0.18)
					Fresh	-0.05 (-0.14 to 0.02)				0.17 (-0.05 to 0.38)
Total intest.	SL + diet + water	371	141.3	0.1	a	**0.40 (0.26 to 0.55)**	0.93 (0.89 to 0.96)	-184.3	0.2	-0.15 (-0.52 to 0.22)
					b	**0.77 (0.66 to 0.89)**				**1.02 (0.91 to 1.13)**
					Omnivory	**0.21 (0.14 to 0.28)**				**0.14 (0.10 to 0.19)**
					Herbivory	**0.49 (0.42 to 0.57)**				**0.36 (0.30 to 0.42)**
					Both	**-0.19 (-0.35 to -0.04)**				-0.05 (-0.20 to 0.11)
					Fresh	**-0.09 (-0.16 to -0.03)**				0.11 (-0.06 to 0.29)
Consistent data (species for which all body size proxies are available)										
Total intest.	BM	293	146.5	103.4	a	**1.76 (1.70 to 1.83)**	0.94 (0.90 to 0.96)	-122.2	76.1	**1.70 (1.31 to 2.07)**
					b	**0.32 (0.27 to 0.36)**				**0.39 (0.34 to 0.44)**
Total intest.	BM + diet	293	43.2	0.0	a	**1.59 (1.53 to 1.65)**	0.89 (0.83 to 0.94)	-198.3	0.0	**1.61 (1.32 to 1.90)**
					b	**0.28 (0.24 to 0.32)**				**0.36 (0.32 to 0.41)**
					Omnivory	**0.24 (0.16 to 0.32)**				**0.15 (0.10 to 0.20)**
					Herbivory	**0.42 (0.34 to 0.49)**				**0.31 (0.25 to 0.38)**
Total intest.	BM + water	293	155.5	112.3	a	**1.77 (1.70 to 1.83)**	0.94 (0.90 to 0.96)	-120.2	78.1	**1.60 (1.19 to 2.00)**
					b	**0.33 (0.28 to 0.39)**				**0.39 (0.35 to 0.44)**
					Both	-0.05 (-0.47 to 0.38)				0.01 (-0.31 to 0.33)
					Fresh	0.03 (-0.06 to 0.12)				0.31 (-0.13 to 0.75)
Total intest.	BM + diet + water	293	52.7	9.5	a	**1.59 (1.53 to 1.65)**	0.89 (0.83 to 0.93)	-196.2	2.1	**1.54 (1.23 to 1.85)**
					b	**0.28 (0.23 to 0.32)**				**0.37 (0.32 to 0.41)**
					Omnivory	**0.25 (0.17 to 0.33)**				**0.15 (0.09 to 0.20)**
					Herbivory	**0.42 (0.35 to 0.50)**				**0.32 (0.26 to 0.38)**
					Both	-0.11 (-0.45 to 0.24)				-0.05 (-0.33 to 0.22)
					Fresh	-0.03 (-0.10 to 0.05)				0.23 (-0.11 to 0.57)

Faunivory used for comparison when dietary proxies used; marine used for comparison when aquatic habitat proxies are used.

Table S11 ***ctd*** Summary statistics for models assessing fish **total intestine length** with body size proxies (body mass (BM), standard length (SL) or total length (TL)) alone, or additionally with a **diet** proxy and **aquatic habitat** (freshwater or marine) according to log(length) = a + b log (BS) + c (diet) + d (water); (significant parameters in **bold**).

			GLS				PGLS			
**Model**		**n**	**AICc**	**ΔAIC**		**parameter (95%CI)**	**lambda (95%CI)**	**AICc**	**ΔAIC**	**parameter (95%CI)**
Continued consistent data (species for which all body size proxies are available)										
Total intest.	TL	293	195.6	107.7	a	**0.42 (0.24 to 0.59)**	0.95 (0.92 to 0.97)	-82.0	80.0	-0.10 (-0.60 to 0.38)
					b	**0.79 (0.65 to 0.93)**				**1.04 (0.89 to 1.19)**
Total intest.	TL + diet	293	87.9	0.0	a	**0.36 (0.22 to 0.50)**	0.92 (0.87 to 0.96)	-162.0	0.0	-0.09 (-0.48 to 0.30)
					b	**0.66 (0.55 to 0.78)**				**0.98 (0.85 to 1.11)**
					Omnivory	**0.51 (0.44 to 0.60)**				**0.15 (0.09 to 0.20)**
					Herbivory	**0.20 (0.12 to 0.28)**				**0.35 (0.28 to 0.42)**
Total intest.	TL + water	293	202.3	114.4	a	**0.53 (0.31 to 0.76)**	0.95 (0.93 to 0.98)	-79.5	82.5	-0.22 (-0.74 to 0.30)
					b	**0.72 (0.56 to 0.89)**				**1.05 (0.89 to 1.20)**
					Both	-0.03 (-0.49 to 0.43)				0.02 (-0.33 to 0.37)
					Fresh	-0.07 (-0.17 to 0.02)				0.32 (-0.19 to 0.83)
Total intest.	TL + diet + water	293	89.7	1.8	a	**0.52 (0.33 to 0.70)**	0.92 (0.87 to 0.96)	-159.6	2.4	-0.17 (-0.59 to 0.24)
					b	**0.63 (0.49 to 0.76)**				**0.99 (0.86 to 1.12)**
					Omnivory	**0.27 (0.19 to 0.36)**				**0.15 (0.09 to 0.20)**
					Herbivory	**0.47 (0.30 to 0.55)**				**0.35 (0.28 to 0.42)**
					Both	-0.11 (-0.48 to 0.26)				-0.04 (-0.34 to 0.25)
					Fresh	**-0.11 (-0.18 to -0.02)**				0.24 (-0.15 to 0.63)
Total intest.	SL	293	198.3	110.2	a	**0.52 (0.35 to 0.68)**	0.95 (0.93 to 0.98)	-80.3	80.7	-0.03 (-0.52 to 0.45)
					b	**0.77 (0.63 to 0.91)**				**1.04 (0.89 to 1.19)**
Total intest.	SL + diet	293	88.1	0.0	a	**0.43 (0.30 to 0.57)**	0.92 (0.87 to 0.96)	-161.0	0.0	-0.01 (-0.40 to 0.36)
					b	**0.72 (0.60 to 0.83)**				**0.98 (0.85 to 1.11)**
					Omnivory	**0.25 (0.17 to 0.34)**				**0.15 (0.10 to 0.20)**
					Herbivory	**0.47 (0.40 to 0.55)**				**0.35 (0.28 to 0.42)**
Total intest.	SL + water	293	204.2	116.1	a	**0.64 (0.44 to 0.84)**	0.96 (0.93 to 0.98)	-77.9	83.1	-0.15 (-0.67 to 0.37)
					b	**0.70 (0.53 to 0.86)**				**1.05 (0.90 to 1.20)**
					Both	-0.02 (-0.48 to 0.44)				0.02 (-0.33 to 0.38)
					Fresh	-0.09 (-0.18 to 0.004)				0.33 (-0.18 to 0.84)
Total intest.	SL + diet + water	293	88.7	0.6	a	**0.59 (0.43 to 0.76)**	0.92 (0.87 to 0.95)	-158.7	2.3	-0.10 (-0.51 to 0.30)
					b	**0.62 (0.48 to 0.75)**				**0.99 (0.86 to 1.12)**
					Omnivory	**0.28 (0.19 to 0.36)**				**0.15 (0.09 to 0.20)**
					Herbivory	**0.48 (0.40 to 0.55)**				**0.35 (0.29 to 0.42)**
					Both	-0.10 (-0.47 to 0.27)				-0.04 (-0.34 to 0.26)
					Fresh	**-0.12 (-0.19 to -0.04)**				0.24 (-0.15 to 0.63)

Faunivory used for comparison when dietary proxies used; marine used for comparison when aquatic habitat proxies are used.

Table S12 Summary statistics for models assessing **freshwater** fish **total intestine length** with body size proxies (body mass (BM), standard length (SL) or total length (TL)) alone, or additionally with a **diet** proxy according to log(length) = a + b log (BS) + c (diet); (significant parameters in **bold**); (almost significant parameters (0.051 – 0.10) in *italics*).

Model		n	GLS AICc	ΔAIC		parameter (95%CI)	PGLS lambda (95%CI)	AICc	ΔAIC	parameter (95%CI)
All data										
Total intest.	BM	191	120.4	115.4	a	**19.2 (1.81 to 2.02)**	0.98 (0.95 to 0.99)	-43.8	77.1	**1.82 (1.28 to 2.37)**
					b	**0.42 (0.36 to 0.48)**				**0.39 (0.33 to 0.45)**
Total intest.	BM + diet	191	5.0	0.0	a	**1.66 (1.57 to 1.75)**	0.94 (0.88 to 0.97)	-120.9	0.0	**1.68 (1.32 to 2.05)**
					b	**0.37 (0.32 to 0.41)**				**0.37 (0.33 to 0.42)**
					Omnivory	**0.16 (0.09 to 0.24)**				**0.14 (0.08 to 0.20)**
					Herbivory	**0.60 (0.51 to 0.69)**				**0.44 (0.36 to 0.53)**
Total intest.	TL	127	65.6	135.6	a	0.01 (-0.23 to 0.24)	0.98 (0.87 to 0.97)	-59.3	77.8	-0.20 (-0.89 to 0.49)
					b	**1.15 (0.93 to 1.37)**				**1.24 (1.02 to 1.47)**
Total intest.	TL + diet	127	-70.0	0.0	a	-0.07 (-0.20 to 0.06)	0.90 (0.97 to NA)	-137.1	0.0	-0.25 (-0.62 to 0.13)
					b	**1.05 (0.93 to 1.17)**				**1.18 (1.03 to 1.34)**
					Omnivory	**0.26 (0.19 to 0.33)**				**0.21 (0.14 to 0.27)**
					Herbivory	**0.67 (0.59 to 0.75)**				**0.51 (0.43 to 0.60)**
Total intest.	SL	175	116.4	131.7	a	-0.04 (-0.23 to 0.16)	0.98 (0.96 to 0.99)	-57.7	90.2	0.10 (-0.53 to 0.73)
					b	**1.29 (1.09 to 1.49)**				**1.18 (0.99 to 1.36)**
Total intest.	SL + diet	175	-15.3	0.0	a	-0.09 (-0.22 to 0.05)	0.95 (0.90 to 0.98)	-147.9	0.0	-0.07 (-0.47 to 0.33)
					b	**1.15 (1.02 to 1.28)**				**1.16 (1.02 to 1.30)**
					Omnivory	**0.19 (1.02 to 1.28)**				**0.17 (0.11 to 0.23)**
					Herbivory	**0.65 (0.57 to 0.74)**				**0.49 (0.40 to 0.57)**
Consistent data (species for which all body size proxies are available)										
Total intest.	BM	119	29.5	128.0	a	**2.16 (2.01 to 2.32)**	0.98 (0.94 to 0.99)	-77.8	70.3	**2.05 (1.39 to 2.71)**
					b	**0.53 (0.45 to 0.62)**				**0.45 (0.37 to 0.52)**
Total intest.	BM + diet	119	-98.5	0.0	a	**1.83 (1.74 to 1.93)**	0.89 (0.72 to 0.96)	-148.1	0.0	**1.84 (1.52 to 2.17)**
					b	**0.44 (0.39 to 0.49)**				**0.42 (0.37 to 0.48)**
					Omnivory	**0.24 (0.17 to 0.30)**				**0.19 (0.13 to 0.25)**
					Herbivory	**0.59 (0.52 to 0.66)**				**0.47 (0.39 to 0.55)**
Total intest.	TL	119	294.8	393.3	a	**0.43 (0.26 to 0.60)**	0.99 (0.96 to 1.00)	-66.2	81.9	-0.09 (-0.89 to 0.71)
					b	**0.72 (0.59 to 0.86)**				**1.27 (1.03 to 1.50)**
Total intest.	TL + diet	119	-82.1	16.4	a	**-0.22 (-0.37 to -0.07)**	0.93 (0.81 to 0.98)	-134.6	13.5	-0.20 (-0.64 to 0.24)
					b	**1.20 (1.06 to 1.35)**				**1.21 (1.04 to 1.38)**
					Omnivory	**0.26 (0.20 to 0.33)**				**0.19 (0.13 to 0.25)**
					Herbivory	**0.67 (0.60 to 0.75)**				**0.50 (0.41 to 0.59)**
Total intest.	SL	119	59.5	158.0	a	-0.06 (-0.32 to 0.20)	0.99 (0.96 to 0.99)	-64.6	83.5	0.02 (-0.78 to 0.81)
					b	**1.36 (1.09 to 1.63)**				**1.28 (1.04 to 1.52)**
Total intest.	SL + diet	119	-80.5	18.0	a	-0.10 (-0.24 to 0.03)	0.92 (0.80 to 0.98)	-130.9	17.2	-0.08 (-0.52 to 0.36)
					b	**1.21 (1.06 to 1.35)**				**1.22 (1.04 to 1.39)**
					Omnivory	**0.26 (0.19 to 0.33)**				**0.18 (0.12 to 0.25)**
					Herbivory	**0.68 (0.60 to 0.75)**				**0.50 (0.41 to 0.59)**

Faunivory used for comparison when dietary proxies used; marine used for comparison when aquatic habitat proxies are used.

Table S13 Summary statistics for models assessing **marine** fish **total intestine length** with body size proxies (body mass (BM), standard length (SL) or total length (TL)) alone, or additionally with a **diet** proxy according to log(length) = a + b log (BS) + c (diet); (significant parameters in **bold**).

Model		n	GLS AICc	ΔAIC		parameter (95%CI)	PGLS lambda (95%CI)	AICc	ΔAIC	parameter (95%CI)
All data										
Total intest.	BM	254	184.3	42.9	a	**1.64 (1.56 to 1.71)**	0.96 (0.93 to 0.98)	-58.5	18.4	**1.24 (0.63 to 1.86)**
					b	**0.30 (0.24 to 0.36)**				**0.35 (0.30 to 0.40)**
Total intest.	BM + diet	254	141.4	0.0	a	**1.51 (1.43 to 1.58)**	0.95 (0.90 to 0.97)	-76.9	0.0	**1.23 (0.68 to 1.79)**
					b	**0.27 (0.21 to 0.32)**				**0.34 (0.29 to 0.39)**
					Omnivory	**0.19 (0.08 to 0.30)**				**0.09 (0.03 to 0.16)**
					Herbivory	**0.38 (0.28 to 0.48)**				**0.20 (0.12 to 0.29)**
Total intest.	TL	240	215.4	46.3	a	**0.62 (0.36 to 0.89)**	0.98 (0.95 to 1.00)	-59.3	12.5	-0.10 (-0.89 to 0.49)
					b	**0.56 (0.37 to 0.76)**				**1.24 (1.02 to 1.47)**
Total intest.	TL + diet	240	169.1	0.0	a	**0.62 (0.38 to 0.85)**	0.96 (0.93 to 0.98)	-46.8	0.0	-0.24 (-0.91 to 0.42)
					b	**0.49 (0.31 to 0.66)**				**0.86 (0.70 to 1.02)**
					Omnivory	**0.17 (0.04 to 0.56)**				0.05 (-0.02 to 0.12)
					Herbivory	**0.45 (0.34 to 0.56)**				**0.22 (0.13 to 0.31)**
Total intest.	SL	183	124.3	37.0	a	**0.91 (0.68 to 1.14)**	0.94 (0.88 to 1.00)	-41.0	18.4	0.15 (-0.34 to 0.64)
					b	**0.47 (0.29 to 0.66)**				**0.84 (0.67 to 1.02)**
Total intest.	SL + diet	183	87.3	0.0	a	**0.82 (0.62 to 1.03)**	0.89 (0.79 to 0.95)	-59.4	0.0	0.21 (-0.22 to 0.63)
					b	**0.44 (0.28 to 0.61)**				**0.79 (0.63 to 0.96)**
					Omnivory	**0.27 (0.14 to 0.39)**				**0.15 (0.07 to 0.23)**
					Herbivory	**0.36 (0.26 to 0.46)**				**0.22 (0.13 to 0.31)**
Consistent data (species for which all body size proxies are available)										
Total intest.	BM	172	99.5	31.0	a	**1.68 (1.61 to 1.76)**	0.90 (0.83 to 0.96)	-65.2	12.8	**1.53 (1.18 to 1.88)**
					b	**0.23 (0.16 to 0.30)**				**0.32 (0.26 to 0.38)**
Total intest.	BM + diet	172	68.5	0.0	a	**1.54 (1.46 to 1.62)**	0.86 (0.75 to 0.93)	-78.0	0.0	**1.51 (1.20 to 1.81)**
					b	**0.20 (0.13 to 0.26)**				**0.30 (0.24 to 0.36)**
					Omnivory	**0.27 (0.15 to 0.40)**				**0.13 (0.05 to 0.21)**
					Herbivory	**0.32 (0.22 to 0.42)**				**0.18 (0.09 to 0.27)**
Total intest.	TL	172	118.7	50.2	a	**0.90 (0.64 to 1.17)**	0.93 (0.86 to 0.97)	-40.3	37.7	0.16 (-0.34 to 0.65)
					b	**0.44 (0.24 to 0.64)**				**0.79 (0.60 to 0.98)**
Total intest.	TL + diet	172	83.5	15.0	a	**0.86 (0.63 to 1.10)**	0.88 (0.78 to 0.95)	-54.7	23.3	0.23 (-0.21 to 0.67)
					b	**0.38 (0.21 to 0.56)**				**0.73 (0.55 to 0.91)**
					Omnivory	**0.29 (0.16 to 0.43)**				**0.13 (0.04 to 0.22)**
					Herbivory	**0.36 (0.25 to 0.46)**				**0.21 (0.12 to 0.31)**
Total intest.	SL	172	118.4	49.9	a	**0.96 (0.73 to 1.20)**	0.93 (0.87 to 0.98)	-41.2	36.8	0.21 (0.28 to 0.70)
					b	**0.43 (0.24 to 0.62)**				**0.79 (0.60 to 0.98)**
Total intest.	SL + diet	172	81.9	13.4	a	**0.90 (0.69 to 1.10)**	0.89 (0.78 to 0.95)	-57.0	21.0	0.27 (-0.16 to 0.70)
					b	**0.39 (0.22 to 0.55)**				**0.74 (0.56 to 0.91)**
					Omnivory	**0.30 (0.16 to 0.43)**				**0.14 (0.06 to 0.23)**
					Herbivory	**0.36 (0.26 to 0.46)**				**0.21 (0.12 to 0.31)**

Faunivory used for comparison when dietary proxies used; marine used for comparison when aquatic habitat proxies are used

Table S14 Summary of results of post hoc Tukey test for comparison of intestinal indices. Note that for each index the number of analyzed species is different.

	**All available data**		**Consistent data**		**Lake Tanganyika's cichlid**		**Consistent data excluding Lake Tanganyika's cichlid fishes**	
	**Relative intestine length related to standard length n=317**		**Relative intestine length related to standard length n=293**		**Relative intestine length related to standard length n=117**		**Relative intestine length related to standard length n=176**	
**Model**	diff	*P* value	diff	*P* value	diff	*P* value	diff	*P* value
Omnivore-Faunivore	0.94 (0.32 to 1.55)	< 0.001	1.17 (0.48 to 1.87)	< 0.001	1.15 (0.64 to 1.65)	< 0.001	1.26 (0.16 to 2.35)	< 0.001
Herbivore-Faunivore	2.81 (2.22 to 3.41)	< 0.001	2.62 (1.98 to 3.26)	< 0.001	4.70 (4.15 to 5.26)	< 0.001	1.41 (0.52 to 2.30)	0.01
Herbivore-Omnivore	1.87 (1.14 to 2.60)	< 0.001	1.44 (0.61 to 2.27)	< 0.001	3.55 (2.90 to 4.21)	< 0.001	0.14 (-1.10 to 1.40)	0.95
	**Relative intestine length related to total length n=375**		**Relative intestine length related to total length n=293**		**Relative intestine length related to total length n=117**		**Relative intestine length related to total length n=176**	
	diff	*P* value	diff	*P* value	diff	*P* value	diff	*P* value
Omnivore-Faunivore	0.76 (0.29 to 1.24)	< 0.001	0.93 (0.35 to 1.50)	< 0.001	0.94 (0.53 to 1.36)	< 0.001	0.96 (0.06 to 1.86)	0.003
Herbivore-Faunivore	2.08 (1.61 to 2.55)	< 0.001	2.01 (1.49 to 2.54)	< 0.001	3.74 (3.29 to 4.20)	< 0.001	1.01 (0.28 to 1.74)	0.03
Herbivore-Omnivore	1.31 (0.72 to 1.90)	< 0.001	1.08 (0.40 to 1.76)	< 0.001	2.79 (2.26 to 3.33)	< 0.001	0.05 (-0.97 to 1.07)	0.99
	**Zihler index n=468**		**Zihler index n=293**		**Zihler index n=117**		**Zihler index n=176**	
	diff	*P* value	diff	*P* value	diff	*P* value	diff	*P* value
Omnivore-Faunivore	2.82 (1.29 to 4.35)	< 0.001	3.72 (1.84 to 5.61)	< 0.001	3.74 (2.18 to 5.29)	< 0.001	3.83 (0.93 to 6.73)	< 0.001
Herbivore-Faunivore	8.57 (7.01 to 10.14)	< 0.001	7.49 (5.75 to 9.23)	< 0.001	13.93 (12.24 to 15.63)	< 0.001	3.94 (1.59 to 6.30)	0.005
Herbivore-Omnivore	5.75 (3.87 to 7.62)	< 0.001	3.76 (1.51 to 6.01)	< 0.001	10.19 (8.18 to 12.20)	< 0.001	0.11 (-3.19 to 3.42)	0.99

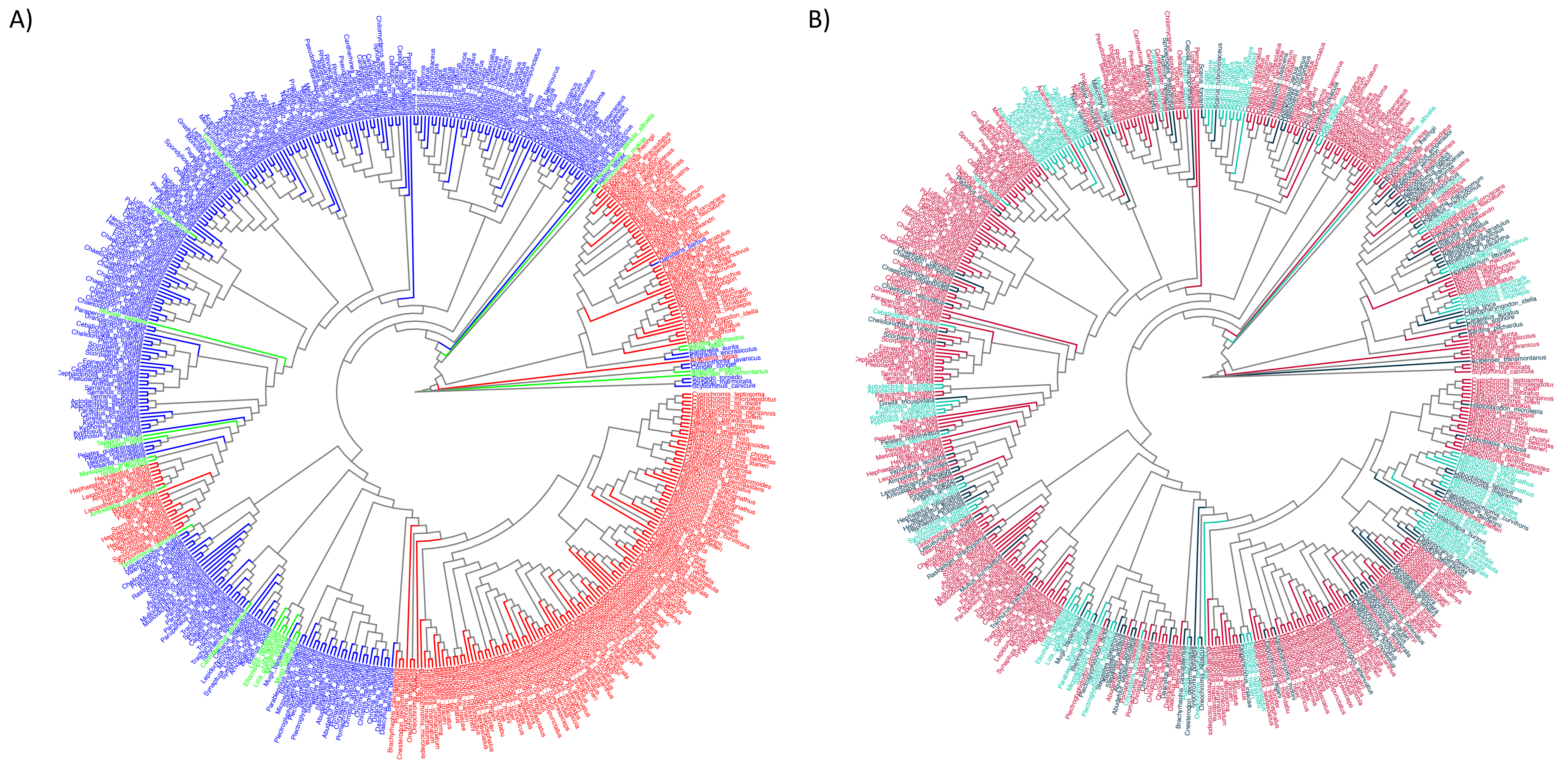

Figure S1: Trait distribution across the piscine phylogenetic tree for all available data n= 468 A) aquatic habitat (red: freshwater; blue: marine and green: both) B) diet (deep red: faunivore; navy blue: omnivore and cyan: herbivore).

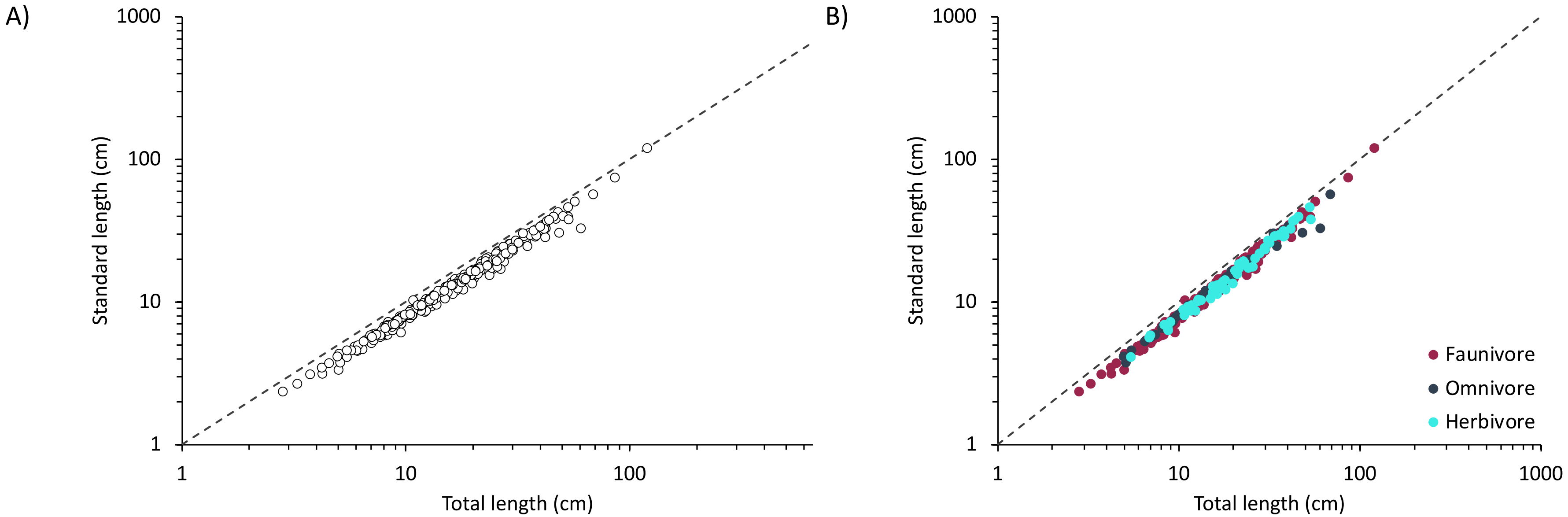

Figure S2: Standard length and total length relationship. A) for all available data, and B) according to diet.

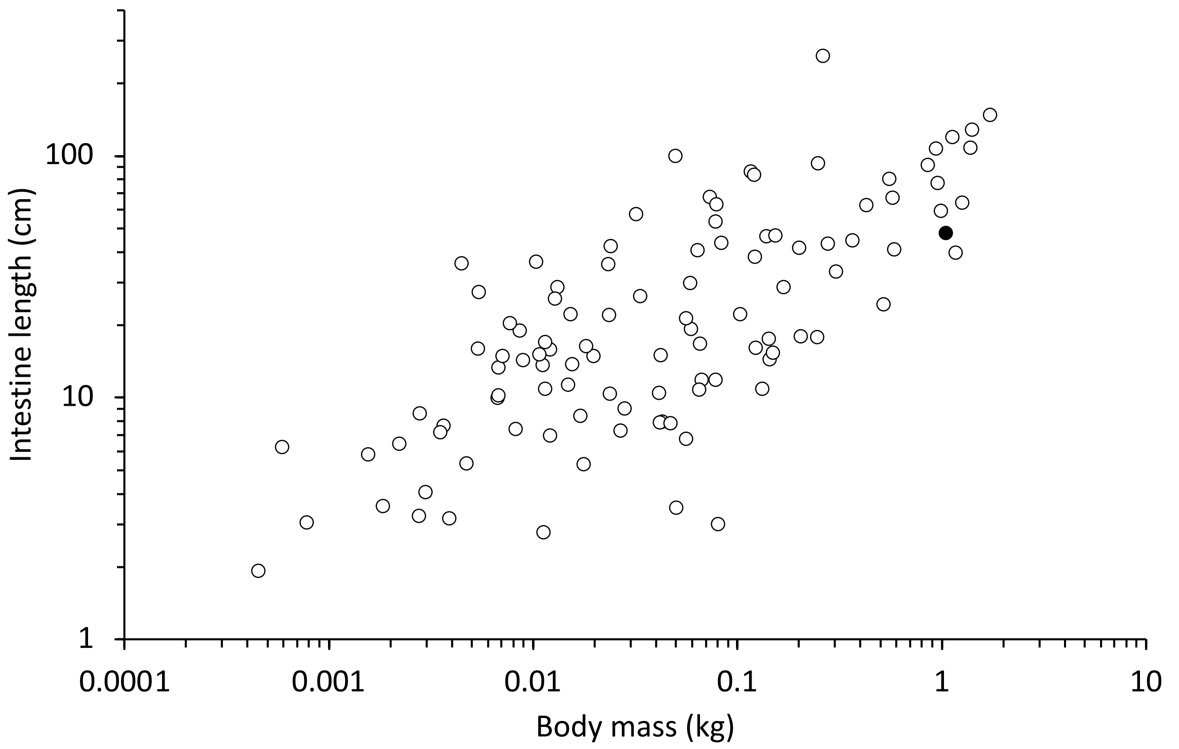

Figure S3: Intestine length and body mass relationship for omnivore fishes. The black dot represents the white sturgeon (*Acipenser transmontanus*), species with intestinal spiral valve.

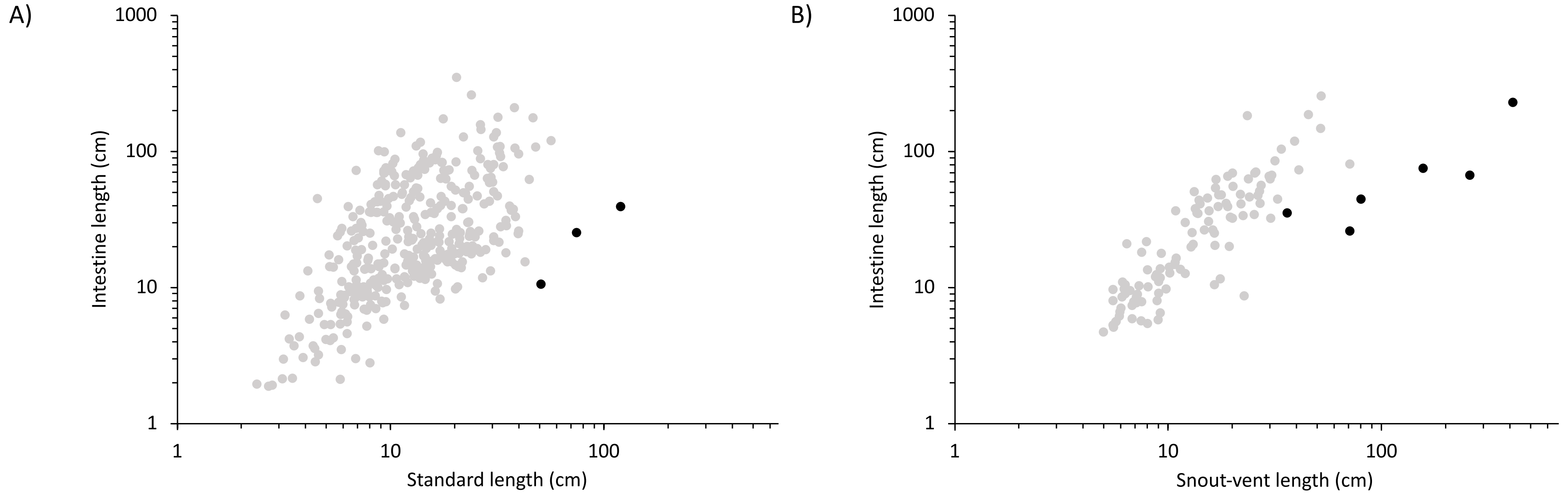

Figure S4: Intestine length and body length relationship A) for fish (standard length used as body length proxy), and B) for reptiles (snout-vent length used a body length proxy. Reptile data taken from Hoppe et al. 2021. Black dots represent elongated species. Elongate fish are the giant moray (*Gymnothorax javanicus*), Chinese trumpetfish (*Aulostomus chinensis*), and Bluespotted cornetfish (*Fistularia commersoni*). Elongated reptiles are dice snake (*Natrix tessellata*), ringed tree boa (*Corallus annulatus*), indian python (*phyton molurus*), reticulated phyton (*Malayopython reticulatus*), striped sand snake (*Psammophis sibilans*), and viperine water snake (*Natrix maura*).

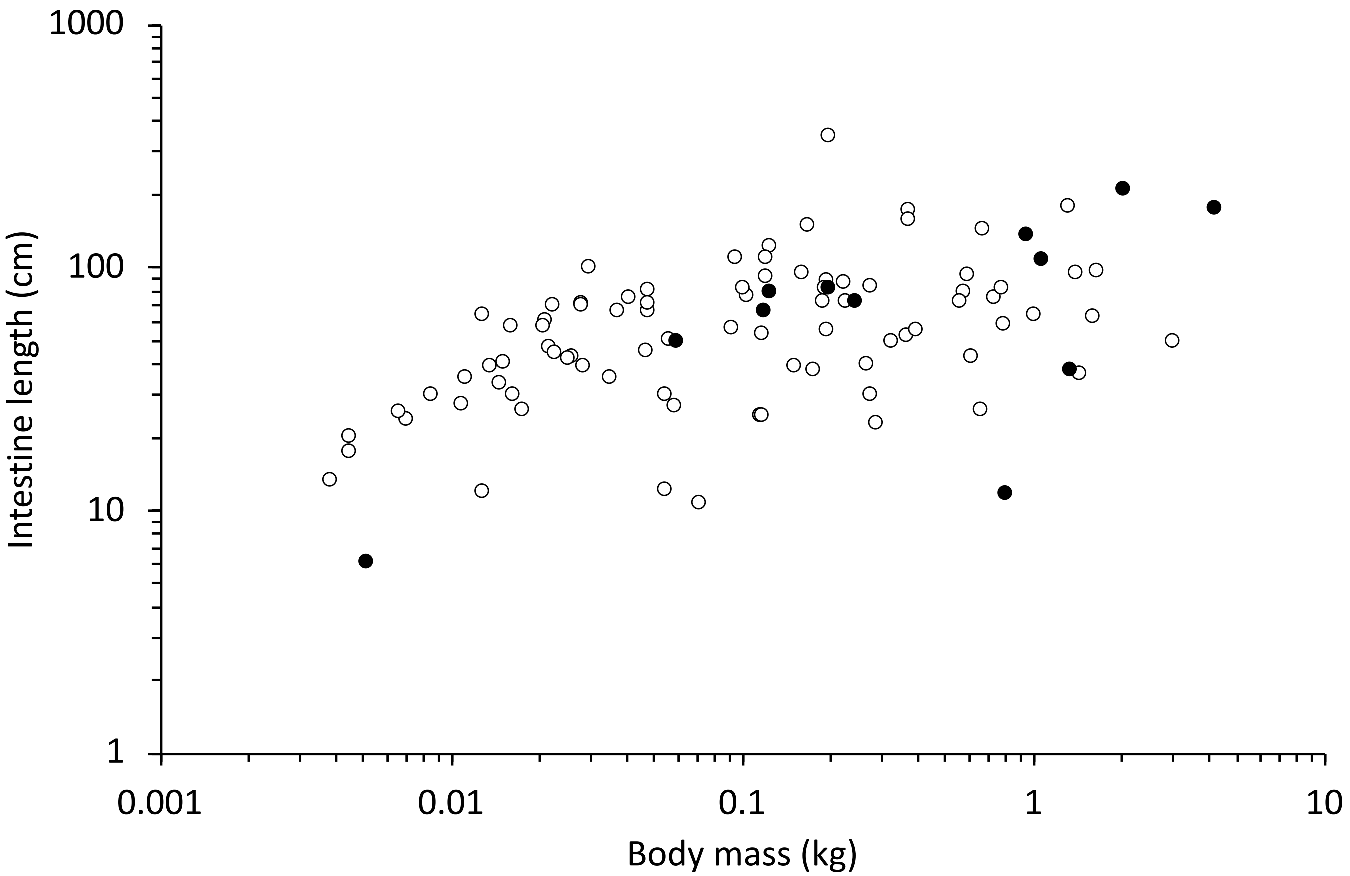

Figure S5: Intestine length and body mass relationship for herbivore fishes. Black dots represent species for which fermentation has been demonstrated (Clements et al. 2014 and 2017).

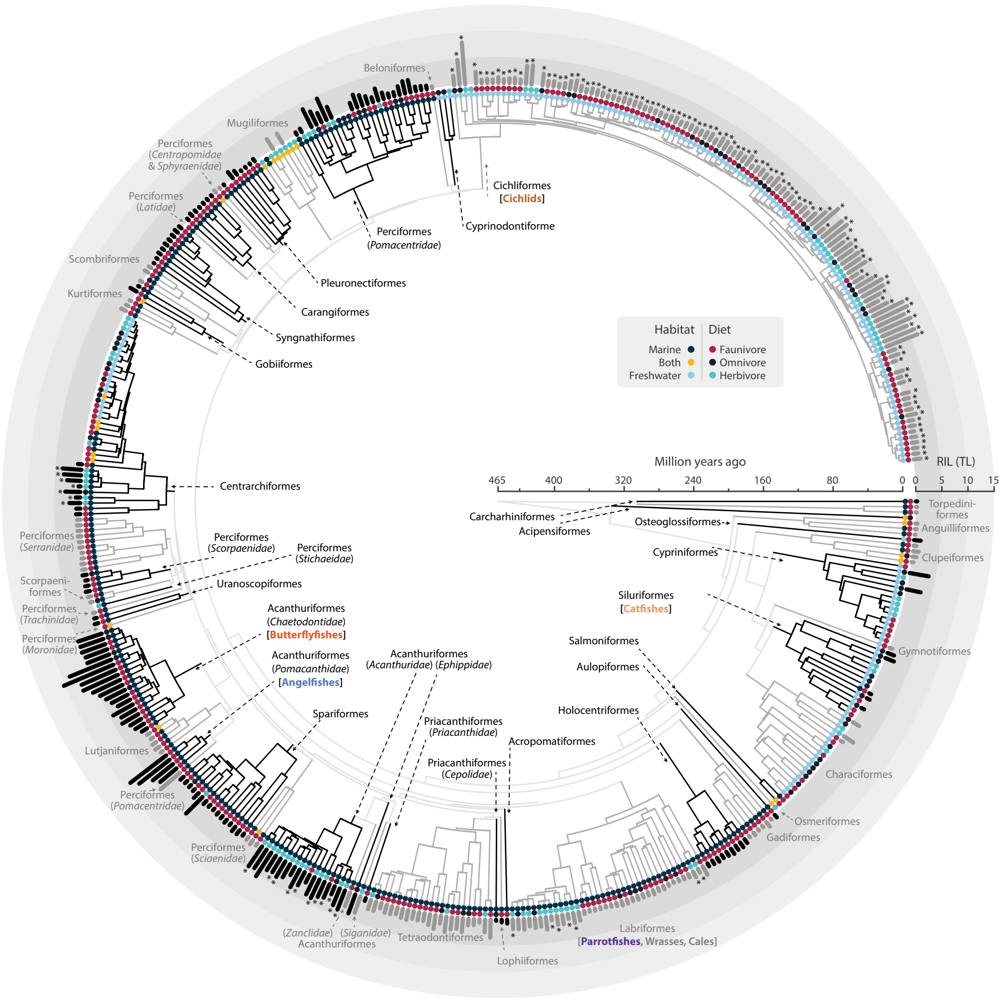

Figure S6: Phylogenetic tree with the 468 species of fishes, inner color tip represents habitat, external colored tip represents diet, and the outer bar relative intestinal length to total length $RIL\_TL = \frac{IL\ (cm)}{TL\ (cm)}$. Species for which original data is reported are marked with a *.

Phylogenetic tree considerations

Median divergence from Torpedo to Scyliorhinus is 272 MYACI: (249.5 - 343.0 MYA).
Further considerations need to be noted.

- *Altolamprologus fasciatus* is ancestral to Lamcal and Neocau. So the species was put as ancestral to Lamcal (bl =3.619026) with a tiny branch of 0.10 from Lamcal .
- *Cyatopharynx fwae* is positioned next to *Cyatopharynx furcifer* with a bl of (0.495937, according to Bonn tree). The distance from *Ophtalmotilapia* is then 1.658172.
- *Cyprichromis sp*. dwarf is the “dwarf jumbo" Cypdwj and is closely related to *Cyprichromis coloratus* with a divergence time of 0.724409 resulting in a further branching time of 3.173421 from the other Cyprichomis.
- *Xenotylapis singularis* was added as sister to *Xenotilapia ochrogenys*, in the tree containing most species it is closer to *Xenotylapia melanogenys*. The latter taxon is the Cichlid tree is named as *Enantiopus melanogenys* and it is basal to both *X. singularis* and *X. ochrogenys* whose time of divergence is set to 0.987114 following the cichlid timeline that is a bit shorter than expected.
- *Eretmodus marksmithi* has been positioned next to Eretmodus_cyanostictus following a divergence time of 2.198621 from cichlid tree. This aligns well with the rest of the tree.
- *Greenwoodochromis staneri* was positioned as ancestral to *Greenwoodochromis christyi* and *Gr_bellcrossi* whose time of divergence appears much shorter. Time of divergence was set to 1.517304 following cichlid tree and, altering the divergence time of the whole *Greenwoodochromis* group to the the *Cyphotilapia* group.
- *Ophthalmotilapia paranasuta* is set near *Ophthalmotilapia nasuta* with time of divergence of 0.8604.
- *Simochromis margaretae* was positioned next to *Simochromis pleurospilus* with a divergence time of 1.808132 following the cichlid tree. Resulting in an elongated branch, keeping consistency with the rest of the tree.
- *Rineloricaria uracantha* was positioned as sister to *Loricariichthys anus* following the phylogeny of Roxo et al. (2019) and Londono-Burbano & Reis (2021). The branch length was arbitrary set to 25.627011 after equal splitting the longer divergence time of Loricariichthys anus from the other taxa set at 51.25 Ma.

Intestine length data references

Aguiar, L. S., de Oliveira, M. I. B., de Matos, L. V., Gomes, A. L. S., da Costa, J. I., & da Silva, G. S. (2018). Distribution of the acanthocephalan *Neoechinorhynchus buttnerae* and semiquantitative analysis of histopathological damage in the intestine of tambaqui (*Colossoma macropomum*). *Parasitology Research*, 117, 1689-1698.

Alioua, Z., Amira, S., Semiane, N., & Zerouali-Khodja, F. (2020). Morphohistology and histopathology of the digestive organs of the deep-water fish greater forkbeard, *Phycis blennoides*. *Fisheries & Aquatic Life*, 28, 99-112.

Alvarado, J. (2009). *Descripción anatómica e histológica del sistema digestivo de juveniles de paiche (Arapaina gigas Cuvier, 1829) criados en jaulas y sometidos a dos tipos de alimentación, en Tingo María*. Universidad Nacional Agraria de la Selva, Tingo María, Perú.

Angelescu, V., & Gneri, F. (1949). Adaptaciones del aparato digestivo al régimen alimeticio en algunos peces del Río Uruguay y del Río de La Plata. *Revista del Instituto Nacional de Investigación de las Ciencias Naturales*, 1, 161-275.

Barcellos Marques, J. F., Branco, E., & Pontes, D. (2014). Aspectos morfométricos do tubo digestório de *Roeboides xenodon* e *Orthospinus franciscensis*. *Biotemas*, 27, 39-147.

Bebić, M., Kević, N., Restović, I., Šantić, M., & Bočina, I. (2020). Histological and histochemical studies of digestive system in the megrim, *Lepidorhombus whiffiagonis* (Teleostei: Scophthalmidae). *Iranian Journal of Ichthyology*, 7, 125-135.

Becker, A. G., Gonçalces, J. F., Garcia, L. O., Behr, E. R., Graça, D. L., Kurtz, F., M., Martins, T., Baldisserotto, B. (2010). Morphometric parameters comparison of the digestive tract of four teleosts with different feeding habits. *Ciência Rural*, 40, 862-866.

Beltrao, H. D., Yamamoto, K. C., & Magalhaes, E. R. S. (2017). Biologia reprodutiva e hábitos alimentares do rodóstomo (*Hemigrammus bleheri*) um peixe ornamental da Bacia do Médio Rio Negro, Estado do Amazonas, Brasil. *Boletim do Instituto de Pesca*, 43, 65-77.

Buddington, R. K., Chen, J. W., & Diamond, J. (1987). Genetic and phenotypic adaptation of intestinal nutrient transport to diet in fish. *The Journal of Physiology*, 393, 261-281.

Canan, B., do Nascimento, W. S., da Silva, N. B., & Chellappa, S. (2012). Morphohistology of the digestive tract of the damsel fish *Stegastes fuscus* (Osteichthyes: Pomacentridae). *Scientific World Journal*, 2012, 787316.

Cleveland, A., & Montgomery, W. (2003). Gut characteristics and assimilation efficiencies in two species of herbivorous damselfishes (Pomacentridae: *Stegastes dorsopunicans* and *S. planifrons*). *Marine Biology*, 142, 35-44.

Cunha, F. E. A., & Rodrigues, R. C. A. (2016). Morfologia do tubo digestivo da pescada Amarela *Cynoscion acoupa* (Lacepède, 1801) (Perciformes: Sciaenidae) no litoral Piauiense, Brasil. *Biota Amazônia*, 6, 32-37.

da Costa, D. L. (2017). *Diferenças morfofisiológicas do trato gastrointestinal de tainhas Mugil liza provenientes de criaçao e do ambiente*. Universidade Federal do Rio Grande, Rio Grande.

Dankwa, H. R., Blay, J., & Yankson, K. (2005). Food and feeding habits of grey mullets (Pisces: Mugilidae) in two estuaries in Ghana. *West African Journal of Applied Ecology*, 8, 0-0.

Das, S., Nandi, S., Majumder, S., & Saikia, S. K. (2013). New characterization of feeding habits of *Puntius sophore* (Hamilton, 1822) through morphometry. *Journal of FisheriesSciences.com*, 7, 225-231.

de Jesus, F. L. (2020). *Análises morfológicas, morfométricas e histoquímicas do tubo digestivo de Brycon amazonicus*. Universidade Federal de Uberlândia, Uberlândia, Minas Gerais.

de Melo Germano, R., Stabille, S. R., de Britto Mari, R., Pereira, J. N. B., Faglioni, J. R. S., & de Miranda Neto, M. H. (2014). Morphological characteristics of the *Pterodoras granulosus* digestive tube (Valenciennes, 1821) (Osteichthyes, Doradidae). *Acta Zoologica*, 95, 166-175.

de Oliveira, M. I. B., de Matos, L. V., da Silva, L. A., Chagas, E. C., da Silva, G. S., & Gomes, A. L. S. (2019). The digestive tube of *Piaractus brachypomus*: gross morphology, histology/histochemistry of the mucosal layer and the effects of parasitism by *Neoechinorhynchus sp*. *Journal of Fish Biology*, 94, 648-659.

do Nascimento, W. S., da Silva, N. B., Yamamoto, M. E., & Chellappa, S. (2013). Anatomy and histology of the digestive tract of a rare annual fish *Hypsolebias antenori* (Rivulidae) from Brazil. *Animal Biology Journal*, 4, 73-84.

Fagundes, K. R., Rotundo, M. M., & Mari, R. B. (2016). Morphological and histochemical characterization of the digestive tract of the puffer fish *Sphoeroides testudineus* (Linnaeus 1758) (Tetraodontiformes: Tetraodontidae). *Anais da Academia Brasileira de Ciências*, 88, 1615-1624.

German, D. P. (2009). Inside the guts of wood-eating catfishes: can they digest wood? *Journal of Comparative Physiology B*, 179, 1011-1023.

German, D. P., Nagle, B. C., Villeda, J. M., Ruiz, A. M., Thomson, A. W., Contreras Balderas, S., & Evans, D. H. (2010). Evolution of herbivory in a carnivorous clade of minnows (teleostei: cyprinidae): effects on gut size and digestive physiology. *Physiological and Biochemical Zoology*, 83, 1-18.

Ghilardi, M., Schiettekatte, N. M. D., Casey, J. M., Brandl, S. J., Degregori, S., Merciere, A., Morat, F., Letourneur, Y., Bejarano, S., Parravicini, V. (2021). Phylogeny, body morphology, and trophic level shape intestinal traits in coral reef fishes. *Ecology and Evolution*, 11, 13218-13231.

Guedes, W. F., Silva, R. M., Moreira, R. M., Pessoa, L. M. B., & de Souza Castro, E. R. R. (2021). Anatomia do tubo digestório da espécie *Acestrorhynchus lacustris* (Ostaryohisi, Characiformes) Lütken, 1875 do rio de Ondas, oesta da Bahia, Brasil. *Veterinária e Zootecnia*, 28, 1-15.

Hidalgo, M. C., Urea, E., & Sanz, A. (1999). Comparative study of digestive enzymes in fish with different nutritional habits. Proteolytic and amylase activities. *Aquaculture*, 170, 267-283.

Jaramillo-Londoño, Á. M. (2009). *Estudio de la biología trófica de cinco especies de peces bentónicos de la costa de Cullera. Relaciones con la acumulación de metales pesados* (Doctoral thesis). Universitat Politécnica de Valencia, Valencia, Spain.

Kalhoro, H., Tong, S., Wang, L., Hua, Y., Volatiana, J. A., & Shao, Q. (2017). Gross anatomical and histomorphological features of the *Acanthopagrus schlegelii* digestive tract (Bleeker 1854) Perciformes, Sparidae. *Acta Zoologica*, 100, 24-35.

Kalhoro, H., Tong, S., Wang, L., Hua, Y., Volatiana, J. A., & Shao, Q. (2018). Morphological study of the gastrointestinal tract of *Larimichthys crocea* (Acanthopterygii: Perciformes). *Zoologia*, 35, 1-9.

Karachle, P. K., & Stergiou, K. I. (2010). Gut length for several marine fish: relationships with body length and trophic implications. *Marine Biodiversity Records*, 3, 1-10.

Kramer, D. L., & Bryant, M. J. (1995). Intestine length in the fishes of a tropical stream: 1. Ontogenetic allometry. *Environmental Biology of Fishes*, 42, 115-127.

Kužir, S., Gjurčević, E., Nejedli, S., Baždarić, B., & Kozarić, Z. (2012). Morphological and histochemical study of intestine in wild and reared European eel (*Anguilla anguilla L.*). *Fish Physiology and Biochemistry*, 38, 625-633.

Machín, E. (2012). *Estudio de la relación entre el nicho trófico, el nicho isotópico y los atributos digestivos en un ensamble de peces*. Universidad de la República, Montevideo, Uruguay.

Makino, L. C. (2010). *Estrutura, ultraestrutura e histoquímica do aparelho digestório Prochilodus lineatus. Análise da diversidade da microbiota intestinal de Prochilodus lineatus e Pterygoplichthys anisitsi*. Universidade Estadual Paulista, Sao Paulo, Brasil.

Mello, G. C. G., Santos, M. L., Arantes, F. P., Pessali, T. C., Brito, M. F. G., & Santos, J. E. (2017). Morphological characterisation of the digestive tract of the catfish *Lophiosilurus alexandri* Steindachner, 1876 (Siluriformes, Pseudopimelodidae). *Acta Zoologica*, 100, 14-23.

Mora Valdés, L. A. (2017). *Alimentación y morfología del sistema alimentario del la damisela gigante Microsphatodon dorsalis* (*Gill, 1862) en sur golfo de California*. Instituto Politécnico Nacional. La paz, Bolivia.

Olaya-Nieto, C., Soto-Fernández, P., & Barrera-Chica, J. (2009). Hábitos alimentarios de la mayupa (*Sternopygus macrurus* Bloch & Schneider, 1801) en el río Sinú, Colombia. *Revista MVZ Córdoba*, 14, 1787-1795.

Özel, O. T., Coskun, I., Cakmak, E., & Cimagil, R. (2019). Histomorphological study on the pyloric caeca and intestine of Black Sea Trout (*Salmo labrax* Pallas, 1814). *Turkish Journal of Agriculture - Food Science and Technology*, 7, 2159-2164.

Ozretić, J., Kević, N., Restović, I., & Bočina, I. (2021). Histological structure and histochemical composition of the digestive tract of salema porgy, *Sarpa salpa* (Linnaeus, 1758) (Teleostei: Sparidae). *Acta Adriatica*, 62, 149-158.

Pereira, T. S. B., Arco, A. D., Hoffmann, P., & Marques, V. B. (2019). Anatomical characterization of the digestive system of the pufferfish (*Chilomycterus spinosus spinosus*). *Acta Scientiarum. Biological Sciences*, 41, e44645 (44641-44646).

Purushothaman, K., Lau, D., Saju, J. M., Musthaq Sk, S., Lunny, D. P., Vij, S., & Orban, L. (2016). Morpho-histological characterisation of the alimentary canal of an important food fish, Asian seabass (*Lates calcarifer*). *PeerJ*, 4, e2377.

Rodrigues, A. P. O., & Cargnin-Ferreira, E. (2017). Morphology and histology of the pirarucu (*Arapaima gigas*) digestive tract. *International Journal of Morphology*, 35, 950-957.

Rodrigues, A. P. O., Pauletti, P., Kindlein, L., Cyrino, J. E. P., Delgado, E. F., & Machado-Neto, R. (2009). Intestinal morphology and histology of the striped catfish *Pseudoplatystoma fasciatum* (Linnaeus, 1766) fed dry diets. *Aquaculture Nutrition*, 15, 559-563.

Rosa, W. S. d., Agostinho, S. C., & Schwarz, K. K. (2020). Morfologia do sistema digestivo de peixes presentes durante o putono no Mercado Municipal de Paranaguá – Pr. Brazilian *Journal of Development*, 6, 84121-84137.

Santos, M. L., Arantes, F. P., Santiago, K. B., & Dos Santos, J. E. (2015). Morphological characteristics of the digestive tract of *Schizodon knerii* (Steindachner, 1875), (Characiformes: Anostomidae): An anatomical, histological and histochemical study. *Anais da Academia Brasileira de Ciências*, 87, 867-878.

Santos, M. L. d., Arantes, F. P., Pessali, T. C., & Santos, J. E. d. (2015). Morphological, histological and histochemical analysis of the digestive tract of *Trachelyopterus striatulus* (Siluriformes: Auchenipteridae). *Zoologia*, 32, 296-305.

Seixas Filho, J. T. D., Brás, J. D. M., Gomide, A. T. D. M., Oliveira, M. G. D. A., Donzele, J. L., & Menin, E. (2000). Anatomia funcional e morfometria dos intestinos e dos cecos pilóricos do Teleostei (Pisces) de água doce *Brycon orbignyanus* (Valenciennes, 1849*). Revista Brasileira de Zoologia*, 29, 313-324.

Seixas Filho, J. T. D., Brás, J. D. M., Gomide, A. T. D. M., Oliveira, M. G. D. A., Donzele, J. L., & Menin, E. (2001). Anatomia funcional e morfometria do intestino no Teleostei (Pisces) de água doce surubim (*Pseudoplatystoma coruscans* - Agassiz, 1829). *Revista Brasileira de Zootecnia*, 30, 1670-1680.

Senarat, S., Kettratad, J., Jiraungoorskul, W., & Kangwanrangsan, N. (2015). Structural classifications in the digestive tract of short mackerel, *Rastrelliger brachysoma* (Bleeker, 1851) from Upper Gulf of Thailand. *Songklanakarin Journal of Science & Technology*, 37, 561-567.

Verdile, N., Pasquariello, R., Scolari, M., Scire, G., Brevini, T. A. L., & Gandolfi, F. (2020). A detailed study of rainbow trout (*Onchorhynchus mykiss*) intestine revealed that digestive and absorptive functions are not linearly distributed along its length. *Animals*, 10, 745 (1-19).

Wagner, C. E., McIntyre, P. B., Buels, K. S., Gilbert, D. M., & Michel, E. (2009). Diet predicts intestine length in Lake Tanganyika's cichlid fishes. *Functional Ecology*, 23, 1122-1131.

Diet references

Agostinho, C. S., Marques, E. E., Oliveira, R. J. d., & Braz, P. S. (2009). Feeding ecology of *Pterodoras granulosus* (Siluriformes, Doradidae) in the Lajeado Reservoir, Tocantins, Brazil. Iheringia. *Série Zoologia*, 99, 301-306.

Alioua, Z., Amira, S., Semiane, N., & Zerouali-Khodja, F. (2020). Morphohistology and histopathology of the digestive organs of the deep-water fish greater forkbeard, *Phycis blennoides*. *Fisheries & Aquatic Life*, 28, 99-112.

Amaral, V., & Cabral, H. N. (2004). Ecology of the whiskered sole in the Sado Estuary, Portugal. *Journal of Fish Biology*, 64, 460-474.

Angelescu, V., & Gneri, F. (1949). Adaptaciones del aparato digestivo al régimen alimeticio en algunos peces del Río Uruguay y del Río de La Plata. *Revista del Instituto Nacional de Investigación de las Ciencias Naturales*, 1, 161-275.

Arias, J. A. (2006). Estado actual del conocimiento sobre el yamú, *Brycon amazonicus*. *Revista Colombiana de Ciencias Pecuarias*, 19, 125-133.

Barcellos Marques, J. F., Branco, E., & Pontes, D. (2014). Aspectos morfométricos do tubo digestório de *Roeboides xenodon* e *Orthospinus franciscensis*. *Biotemas*, 27, 139-147.

Bebić, M., Kević, N., Restović, I., Šantić, M., & Bočina, I. (2020). Histological and histochemical studies of digestive system in the megrim, *Lepidorhombus whiffiagonis* (Teleostei: Scophthalmidae). *Iranian Journal of Ichthyology*, 7, 125-135.

Becker, A. G., Gonçalces, J. F., Garcia, L. O., Behr, E. R., Graça, D. L., Kurtz, F., M., Martins, T., Baldisserotto, B. (2010). Morphometric parameters comparison of the digestive tract of four teleosts with different feeding habits. *Ciência Rural*, 40, 862-866.

Bessey, C., & Heithaus, M. R. (2015). Ecological niche of an abundant teleost *Pelates octolineatus* in a subtropical seagrass ecosystem. *Marine Ecology Progress Series*, 541, 195-204.

Blaber, S. J. M., & Cyrus, D. P. (1983). The biology of Carangidae (Teleostei) in Natal estuaries. *Journal of Fish Biology*, 22, 173-188.

Buck, S., & Sazima, I. (1995). An assemblage of mailed catfishes (Loricariidae) in southeastern Brazil: distribution, activity, and feeding. *Ichthyological Exploration of Freshwaters*, 6, 325-332.

Buddington, R. K., Chen, J. W., & Diamond, J. (1987). Genetic and phenotypic adaptation of intestinal nutrient transport to diet in fish. *The Journal of Physiology*, 393, 261-281.

Buddington, R. K., & Doroshov, S. (1986). Structural and functional relations of the white sturgeon alimentary canal (*Acipenser transmontanus*). *Journal of Morphology*, 190, 201-213.

Canan, B., do Nascimento, W. S., da Silva, N. B., & Chellappa, S. (2012). Morphohistology of the digestive tract of the damsel fish *Stegastes fuscus* (Osteichthyes: Pomacentridae). *Scientific World Journal*, 2012, 787316.

Capapé, C., Crouzet, S., Clément, C., Vergne, Y., & Guñelorget, O. (2007). Diet of the marbled electric ray *Torpedo marmorata* (Chondrichthyes: Torpedinidae) off the Languedocian coast (Southern France, Northern Mediterranean). *Annales, Series Historia Naturalis*, 17-22.

Carrassón, M., Grau, A., Dopazo, L. R., & Crespo, S. (2006). A histological, histochemical and ultrastructural study of the digestive tract of *Dentex dentex* (Pisces, Sparidae). *Histology and Histopatology*, 21, 579-593.

Castro, J. J., & Santana-del-Pino. (1995). Feeding preferences of *Scomber japonicus* in the Canary Islands area. *Scientia Marina*, 59, 325-333.

Chi-Espínola, A. A., & Vega-Cendejas, M. E. (2013). Hábitos alimenticios de *Sphoeroides testudineus* (Perciformes: Tetraodontidae) en el sistema lagunar de Ría Lagartos, Yucatán, México. *Revista de Biología Tropical*, 61, 849-858.

Choat, J., Clements, K. D., & Robbins, W. D. (2002). The trophic status of herbivorous fishes on coral reefs. *Marine Biology*, 140, 613-623.

Cooper, W. J., Carter, C. B., Conith, A. J., Rice, A. N., & Westneat, M. W. (2017). The evolution of jaw protrusion mechanics is tightly coupled to bentho-pelagic divergence in damselfishes (Pomacentridae). *Journal of Experimental Biology*, 220, 652-666.

Correa, S. B., Mérona, B. d., & Armbruster, J. W. (2015). Diet shift of Red Belly Pacu *Piaractus brachypomus* (Cuvier, 1818)(Characiformes: Serrasalmidae), a Neotropical fish, in the Sepik-Ramu River Basin, Papua New Guinea. *Neotropical Ichthyology*, 12, 827-833.

Costa, D. L. d. (2017). *Diferenças morfofisiológicas do trato gastrointestinal de tainhas Mugil liza provenientes de criação e do ambiente*. Universidade Federal do Rio Grande.

Dankwa, H., Blay Jr, J., & Yankson, K. (2005). Food and feeding habits of grey mullets (Pisces: Mugilidae) in two estuaries in Ghana. *West African Journal of Applied Ecology*, 8, 0-0.

Das, S., Nandi, S., Majumder, S., & Saikia, S. K. (2013). New characterization of feeding habits of *Puntius sophore* (Hamilton, 1822) through morphometry. *Journal of FisheriesSciences.com*, 7, 225-231.
Davis, A. M., Pearson, R. G., Pusey, B. J., Perna, C., Morgan, D. L., & Burrows, D. (2011). Trophic ecology of northern Australia's terapontids: ontogenetic dietary shifts and feeding classification. *Journal of Fish Biology*, 78, 265-286.
Davis, A. M., Unmack, P. J., Pusey, B. J., Pearson, R. G., & Morgan, D. L. (2013). Ontogenetic development of intestinal length and relationships to diet in an Australasian fish family (Terapontidae). *BMC Evolutionary Biology*, 13, 1-16.
de Jesus, F. L. (2020). *Análises morfológicas, morfométricas e histoquímicas do tubo digestivo de Brycon amazonicus*. Universidade Federal de Uberlândia, Uberlândia, Minas Gerais.
de Melo Germano, R., Stabille, S. R., de Britto Mari, R., Pereira, J. N. B., Faglioni, J. R. S., & de Miranda Neto, M. H. (2014). Morphological characteristics of the *Pterodoras granulosus* digestive tube (Valenciennes, 1821) (Osteichthyes, Doradidae). *Acta Zoologica*, 95, 166-175.
De Troch, M., Mees, J., & Wakwabi, E. (1998). Diets of abundant fishes from beach seine catches in seagrass beds of a tropical bay (Gazi Bay, Kenya). *Belgian Journal of Zoology*, 128, 135-154.
Dorman, J. A. (1988). Diet of the garfish, *Belone belone* (L.), from Courtmacsherry Bay, Ireland. *Journal of Fish Biology*, 33, 339-346.
dos Anjos, H. D. B., Yamamoto, K. C., & Magalhães, E. R. S. (2017). Biologia reprodutiva e hábitos alimentares do rodóstomo (*Hemigrammus bleheri*) um peixe ornamental da Bacia do Médio Rio Negro, Estado do Amazonas, Brasil. *Boletim do Instituto de Pesca*, 43, 65Ã 77.
Dromard, C. R., Bouchon-Navaro, Y., Cordonnier, S., Fontaine, M.-F., Verlaque, M., Harmelin-Vivien, M., & Bouchon, C. (2013). Resource use of two damselfishes, *Stegastes planifrons* and *Stegastes adustus*, on Guadeloupean reefs (Lesser Antilles): Inference from stomach content and stable isotope analysis. *Journal of Experimental Marine Biology and Ecology*, 440, 116-125.
Ekpo, I. E., Essien-Ibok, M. A., & Nkwoji, J. N. (2014). Food and feeding habits and condition factor of fish species in Qua Iboe River estuary, Akwa Ibom State, southeastern Nigeria. *International Journal of Fisheries and Aquatic Studies*, 2, 38-46.
Escalante, A. H. (1987). Dieta comparativa de *Cheirodon* I. *interruptus* (Osteichthyes Characidae) en ambientes lénticos y lóticos de la Provincia de Buenos Aires. *Revista del Museo de La Plata*, 14, 35-45.
Fanelli, E., Badalamenti, F., D'Anna, G., Pipitone, C., Riginella, E., & Azzurro, E. (2011). Food partitioning and diet temporal variation in two coexisting sparids, *Pagellus erythrinus* and *Pagellus acarne*. *Journal of Fish Biology*, 78, 869-900.
Garrido, S., Ben-Hamadou, R., Oliveira, P. B., Cunha, M. E., Chícharo, M. A., & van der Lingen, C. D. (2008). Diet and feeding intensity of sardine *Sardina pilchardus*: correlation with satellite-derived chlorophyll data. *Marine Ecology Progress Series*, 354, 245-256.
German, D. P. (2009). Inside the guts of wood-eating catfishes: can they digest wood? *Journal of Comparative Physiology B*, 179, 1011-1023.
German, D. P., Nagle, B. C., Villeda, J. M., Ruiz, A. M., Thomson, A. W., Contreras Balderas, S., & Evans, D. H. (2010). Evolution of herbivory in a carnivorous clade of minnows (teleostei: cyprinidae): effects on gut size and digestive physiology. *Physiological and Biochemical Zoology*, 83, 1-18.
Gomes, L. d. C., Golombieski, J. I., Gomes, A. R. C., & Baldisserotto, B. (2000). Biologia do jundiá *Rhamdia quelen* (TELEOSTEI, PIMELODIDAE). *Ciência Rural*, 30, 179-185.

Guedes, A. P. P., Araújo, F. G., Pessanha, A. L., & Milagre, R. R. (2015). Partitioning of the feeding niche along spatial, seasonal and size dimensions by the fish community in a tropical Bay in Southeastern Brazil. *Marine Ecology*, 36, 38-56.

Guedes, A. P. P., Araújo, F. G., Pessanha, A. L. M., & Milagre, R. R. (2015). Partitioning of the feeding niche along spatial, seasonal and size dimensions by the fish community in a tropical Bay in Southeastern Brazil. *Marine Ecology*, 36, 38-56.

Guzmán-Beltran, L., Santana, D., Verdugo, H., Gómez-Ramirez, E., & Hurtado Giraldo, H. (2013). Descripción anatómica e histológica del tracto digestivo del Nicuro *Pimelodus blochii* (Valenciennes, 1840). *Orinoquia*, 17, 102-110.

Hajisamae, S., Soe, K. K., Pradit, S., Chaiyvareesajja, J., & Fazrul, H. (2022). Feeding habits and microplastic ingestion of short mackerel, *Rastrelliger brachysoma*, in a tropical estuarine environment. *Environmental Biology of Fishes*, 105, 289-302.

Harding, J. M., & Mann, R. (2001). Diet and habitat use by bluefish, *Pomatomus saltatrix*, in a Chesapeake Bay estuary. *Environmental Biology of Fishes*, 60, 401-409.

Harmelin-Vivien, M., & Bouchon, C. (1976). Feeding behavior of some carnivorous fishes (Serranidae and Scorpaenidae) from Tulear (Madagascar). *Marine Biology*, 37, 329-340.

Hiatt, R. W., & Strasburg, D. W. (1960). Ecological relationships of the fish fauna on coral reefs of the Marshall Islands. *Ecological Monographs*, 30, 65-127.

Hidalgo, M. C., Urea, E., & Sanz, A. (1999). Comparative study of digestive enzymes in fish with different nutritional habits. Proteolytic and amylase activities. *Aquaculture*, 170, 267-283.

Ho, C.-T., Fu, Y.-C., Sun, C.-L., Kao, S.-J., & Jan, R.-Q. (2009). Plasticity of feeding habits of two Plectroglyphidodon damselfishes on coral reefs in southern Taiwan: evidence from stomach content and stable isotope analyses. *Zoological Studies*, 48, 649-656.

Jacobi, C. M., Villamarin, F., Campos-Silva, J. V., Jardine, T., & Magnusson, W. E. (2020). Feeding of Arapaima sp.: integrating stomach contents and local ecological knowledge. *Journal of Fish Biology*, 9, 265-272.

Jaramillo-Londoño, Á. M. (2009). E*studio de la biología trófica de cinco especies de peces bentónicos de la costa de Cullera. Relaciones con la acumulación de metales pesados* (Doctoral thesis). Universitat Politécnica de Valencia, Valencia, Spain.

Jones, R. S. (1968). Ecological relationships in Hawaiian and Johnston Island Acanthuridae (surgeonfishes). *Micronesica*, 4, 309-361.

Kalhoro, H., Tong, S., Wang, L., Hua, Y., Volatiana, J. A., & Shao, Q. (2018). Morphological study of the gastrointestinal tract of *Larimichthys crocea* (Acanthopterygii: Perciformes). *Zoologia*, 35, 1-9.

Karachle, P. K. (2017). Diet composition and overlap for 43 dishes in the North Aegean Sea, Greece. *Acta Adriatica*, 58, 125-136.

Karachle, P. K., & Stergiou, K. I. (2010). Gut length for several marine fish: relationships with body length and trophic implications. *Marine Biodiversity Records*, 3, 1-10.

Kirkagaç, M. U. (2003). The gut contents of grass carp, *Ctenopharyngodon idella*, during nursing in an earthen pond. *The Israeli Journal of Aquaculture*, 55, 139-143.

Kokubun, É. E., Bonato, K. O., Burress, E. D., & Fialho, C. B. (2018). Diet and body shape among populations of *Bryconamericus iheringii* (Otophysi: Characidae) across the Campos Sulinos ecosystem. *Neotropical Ichthyology*, 16, e170167.

Konings, A. (2019). Tanganyika Cichlids in their natural habitat, 4th edition: Cichlid Press.
Kramer, D. L., & Bryant, M. J. (1995). Intestine length in the fishes of a tropical stream: 2. Relationships to diet - the long and short of a convoluted issue. *Environmental Biology of Fishes*, 42, 129-141.
Kužir, S., Gjurčević, E., Nejedli, S., Baždarić, B., & Kozarić, Z. (2012). Morphological and histochemical study of intestine in wild and reared European eel (*Anguilla anguilla* L.). *Fish Physiology and Biochemestry*, 38, 625-633.
Lamboj, A. (2004). The cichlid fishes of western Africa. Birgit Schmettkamp Verlag. ISBN-10: 392881933X
Lefebvre, S. E. (2014). *Is diet correlated with feeding morphology in neotropical suckermouth armoured catfishes (Siluriformes: Loricariidae)?* University of Toronto,
Lira, A. S., Frédou, F. L., Viana, A. P., Eduardo, L. N., & Frédou, T. (2017). Feeding ecology of *Centropomus undecimalis* (Bloch, 1792) and Centropomus parallelus (Poey, 1860) in two tropical estuaries in Northeastern Brazil. *Pan-American Journal of Aquatic Sciences*, 12, 123-135.
Lyle, J. (1983). Food and feeding habits of the lesser spotted dogfish, *Scyliorhinus canicula* (L.), in Isle of Man waters. *Journal of Fish Biology*, 23, 725-737.
Machín, E. (2012). *Estudio de la relación entre el nicho trófico, el nicho isotópico y los atributos digestivos en un ensamble de peces*. (M.Sc dissertation in Biology). Universidad de la Republica, Montevideo, Uruguay.
McClure, M. M., McIntyre, P. B., & McCune, A. R. (2006). Notes on the natural diet and habitat of eight danionin fishes, including the zebrafish *Danio rerio*. *Journal of Fish Biology*, 69, 553-570.
Mello, G. C. G., Santos, M. L., Arantes, F. P., Pessali, T. C., Brito, M. F. G., & Santos, J. E. (2017). Morphological characterisation of the digestive tract of the catfish *Lophiosilurus alexandri* Steindachner, 1876 (Siluriformes, Pseudopimelodidae). *Acta Zoologica*, 100, 14-23.
Montgomery, W. L. (1980a). Comparative feeding ecology of two herbivorous damselfishes (Pomacentridae: Teleostei) from the Gulf of California, Mexico. *Journal of Experimental Marine Biology and Ecology*, 47, 9-24.
Montgomery, W. L. (1980b). The impact of non-selective grazing by the giant blue damselfish, *Microspathodon dorsalis*, on algal communities in the Gulf of California, Mexico. *Bulletin of Marine Science*, 30, 290-303.
Mora Valdés, L. A. (2017). *Alimentación y morfología del sistema alimentario del la damisela gigante Microsphatodon dorsalis (Gill, 1862) en sur golfo de California*. Instituto Politécnico Nacional La paz, Bolivia.
Morato, T., Solá, E., Grós, M. P., & Menezes, G. (2001). Feeding habits of two congener species of seabreams, *Pagellus bogaraveo* and *Pagellus acarne*, off the Azores (Northeastern Atlantic) during spring of 1996 and 1997. *Bulletin of Marine Science*, 69, 1073-1087.
Morte, M. S., Redón, M. J., & Sanz-Brau, A. (2002). Feeding habits of *Trisopterus minutus capelanus* (Gadidae) off the eastern coast of Spain (western Mediterranean). *Marine Ecology*, 22, 215-229.
Muschick, M., Indermaur, A., & Salzburger, W. (2012). Convergent evolution within an adaptive radiation of cichlid fishes. *Current Biology*, 22, 2362-2368.

Nakagawa, H., Umino, T., Sekimoto, T., Ambas, I., Montgomery, W. L., & Nakano, T. (2002). Characterization of the digestive tract of wild ayu. *Fisheries Science*, 68, 341-346.

Neto, J., Reis, G., Vasconcelos, V., Guimarães, I., & Santos, E. (2028). Morfologia comparativa do trato digestório de tilápias do Nilo (*Oreochromis niloticus*) cultivadas em sistema semi-intensivo vs. da pesca artesanal. *Jornal Interdisciplinar de Biociencias*, 3, 19-24.

Novakowski, G. C., Hahn, N. S., & Fugi, R. (2008). Diet seasonality and food overlap of the fish assemblage in a pantanal pond. *Neotropical Ichthyology*, 6, 567-576.

Olaya-Nieto, C., Soto-Fernández, P., & Barrera-Chica, J. (2009). Hábitos alimentarios de la mayupa (Sternopygus macrurus Bloch & Schneider, 1801) en el río Sinú, Colombia. *Revista MVZ Córdoba*, 14, 1787-1795.

Oliveira, A. C. B., Martinelli, L. A., Moreira, M. Z., Soares, M. G. M., & Cyrino, J. E. P. (2006). Seasonality of energy sources of *Colossoma macropomum* in a floodplain lake in the Amazon–lake Camaleão, Amazonas, Brazil. *Fisheries Management and Ecology*, 13, 135-142.

Oliveira, C. D., Lessa, R., Almeida, Z., & Santana, F. M. (2020). Biology and fishery of Acoupa Weakfish *Cynoscion acoupa* (Lacepède, 1801): a review. *Neotropical Biology and Conservation*, 15, 333-349.

Ozretić, J., Kević, N., Restović, I., & Bočina, I. (2021). Histological structure and histochemical composition of the digestive tract of salema porgy, *Sarpa salpa* (Linnaeus, 1758) (Teleostei: Sparidae). *Acta Adriatica*, 62, 149-158.

Pallaoro, A., Santic, M., & Jardas, I. (2003). Feeding habits of the saddled bream, *Oblada melanura* (Sparidae), in the Adriatic sea. *Cybium*, 27, 261-268.

Parravicini, V., Casey, J. M., Schiettekatte, N. M. D., Brandl, S. J., Pozas-Schacre, C., Carlot, J., Edgar, G.J., Graham, N.A.J., Harmelin-Vivien, M., Kulbicki, M., Stuart-Smith, R. D. (2020). Delineating reef fish trophic guilds with global gut content data synthesis and phylogeny. *PLoS Biology*, 18, e3000702.

Payne, A. I. (1976). The relative abundance and feeding habits of the grey mullet species occurring in an estuary in Sierra Leone, West Africa. *Marine Biology*, 35, 277-286.

Pereira, G. S., de Pereira, M. E., & Weibezahn, F. (1982). Contribución al conocimiento de la ecología alimentaria de algunos peces del lago de Valencia (Venezuela). *Memorias de la Sociedad de Ciencias Naturales*, 17, 41-56.

Pessoa, E. K. R., Silva, N. B., Chellappa, N. T., Souza, A. A., & Chellappa, S. (2013). Morfologia comparativa do trato digestório dos peixes *Hoplias malabaricus* e *Hypostomus pusarum* do Açude Marechal Dutra, Rio Grande do Norte, Brasil. *Biota Amazônia*, 3, 48-57.

Pita, C., Gamito, S., & Erzini, K. (2002). Feeding habits of the gilthead seabream (*Sparus aurata*) from the Ria Formosa (southern Portugal) as compared to the black seabream (Spondyliosoma cantharus) and the annular seabream (*Diplodus annularis*). *Journal of Applied Ichthyology*, 18, 81-86.

Poll, M. (1956) Poisson cichlidae. Résultat Sci Explor Hydrobiol du Lac Tanganika 3:1–619

Plounevez, S., & Champalbert, G. (2000). Diet, feeding behaviour and trophic activity of the anchovy (*Engraulis encrasicolus* L.) in the Gulf of Lions (Mediterranean Sea). *Oceanologia Acta*, 23, 175-192.

Purushothaman, K., Lau, D., Saju, J. M., Musthaq Sk, S., Lunny, D. P., Vij, S., & Orban, L. (2016). Morpho-histological characterisation of the alimentary canal of an important food fish, Asian seabass (*Lates calcarifer*). *PeerJ*, e2377.

Quintans, F., Scasso, F., Loureiro, M., & Yafe, A. (2009). Diet of *Cnesterodon decemmaculatus* (Poeciliidae) and *Jenynsia multidentata* (Anablepidae) in a hypertrophic shallow lake of Uruguay. *Iheringia. Série Zoologia*, 99, 99-105.

Randall, J. E. (2013). Review of the Indo-Pacific labrid fish genus Hemigymnus. *Journal of the Ocean Science Foundation*, 6, 2-18.

Randall, J. E., & Brock, V. E. (1960). Observations on the ecology of epinepheline and lutjanid fishes of the Society Islands, with emphasis on food habits. *Transactions of the American Fisheries Society*, 89, 9-16.

Resen, A. K. (2016). Occurence, food nature, age and growth of orbiculate batfish *Palatax orbicularis* (Ephippidae) in Iraqui territorial marine waters. *Basrah Journal of Veterinary Research*, 15, 65-72.

Rizkalla, S. I., & Philips, A. I. (2008). Feeding habits of the Atlantic stargazer fish *Uranoscopus scaber* Linnaeus, 1758 (Family: Uranoscopidae) in Egyptian Mediterranean waters. *Egyptian Journal of Aquatic Biology and Fisheries*, 12, 1-11.

Robertson, D. R., Polunin, N. V. C., & Leighton, K. (1979). The behavioral ecology of three Indian Ocean surgeonfishes (*Acanthurus lineatus*, *A. Leucosternon* and *Zebrasoma scopas*): their feeding strategies, and social and mating systems. *Environmental Biology of Fishes*, 4, 125-170.

Rodrigues, A. P. O., & Cargnin-Ferreira, E. (2017). Morphology and histology of the pirarucu (*Arapaima gigas*) digestive tract. *International Journal of Morphology*, 35, 950-957.

Rodrigues, A. P. O., Pauletti, P., Kindlein, L., Cyrino, J. E. P., Delgado, E. F., & Machado-Neto, R. (2009). Intestinal morphology and histology of the striped catfish *Pseudoplatystoma fasciatum* (Linnaeus, 1766) fed dry diets. *Aquaculture Nutrition*, 15, 559-563.

Rojas, J. E., Soca, L. A., & García, G. I. (2005). Contenido del tracto digestivo de cuatro especies de peces autóctonos y sus implicaciones como biorreguladores de larvas de mosquitos en Venezuela, 2004. *Revista Cubana de Medicina Tropical*, 57, 0-0.

Ronco, F., Matschiner, M., Böhne, A., Boila, A., Büscher, H. H., El Taher, A., Indermaur, A., Malinsky, M., Ricci, V., Kahmen, A., Jentoft, S., Salzburger, W. (2021). Drivers and dynamics of a massive adaptive radiation in cichlid fishes. *Nature*, 589, 76-81.

Šantić, M., Rađa, B., & Pallaoro, A. (2012). Feeding habits of small-spotted catshark (*Scyliorhinus canicula* Linnaeus, 1758) from the eastern central Adriatic Sea. *Marine Biology Research*, 8, 1003-1011.

Santos, M. L., Arantes, F. P., Santiago, K. B., & Dos Santos, J. E. (2015). Morphological characteristics of the digestive tract of *Schizodon knerii* (Steindachner, 1875), (Characiformes: Anostomidae): An anatomical, histological and histochemical study. *Anais da Academia Brasileira de Ciências*, 87, 867-878.

Santos, M. L. d., Arantes, F. P., Pessali, T. C., & Santos, J. E. d. (2015). Morphological, histological and histochemical analysis of the digestive tract of *Trachelyopterus striatulus* (Siluriformes: Auchenipteridae). *Zoologia*, 32, 296-305.

Seixas Filho, J. T. D., Brás, J. D. M., Gomide, A. T. D. M., Oliveira, M. G. D. A., Donzele, J. L., & Menin, E. (2001). Anatomia funcional e morfometria do intestino no Teleostei (Pisces) de água doce surubim (*Pseudoplatystoma coruscans*-Agassiz, 1829). *Revista Brasileira de Zootecnia*, 30, 1670-1680.

Silva, A. T., & Goitein, R. (2009). Diet and feeding activity of *Acestrorhynchus lacustris* (Lütken, 1875) (Characiformes, Acestrorhynchidae) in the water reservoir at Ribeirão Claro, SP. *Brazilian Journal of Biology*, 69, 757-762.

Spence, R., Fatema, M. K., Ellis, S., Ahmed, Z. F., & Smith, C. (2007). Diet, growth and recruitment of wild zebrafish in Bangladesh. *Journal of Fish Biology*, 71, 304-309.

Tavares, M. T. M., & Di Beneditto, A. P. M. (2017). Feeding habits and behaviour of *Bagre bagre* and *Genidens barbus*, two ariid catfishes (Pisces: Siluriformes) from southeastern Brazil. *Journal of Threatened Taxa*, 9, 10771-10775.

Theis, A., Ronco, F., Indermaur, A., Salzburger, W., & Egger, B. (2014). Adaptive divergence between lake and stream populations of an East African cichlid fish. *Molecular Ecology*, 23, 5304-5322.

Tonella, L. H., Dias, R. M., Vitorino Junior, O. B., Fugi, R., & Agostinho, A. A. (2019). Conservation status and bio-ecology of *Brycon orbignyanus* (Characiformes: Bryconidae), an endemic fish species from the Paraná River basin (Brazil) threatened with extinction. *Neotropical Ichthyology*, 17, e190030.

Turra, A., Santos, F. B., Bessa, E., Fernandez, W. S., Bernadochi, L. C., & Deanadai, M. R. (2012). Population biology and diet of the southern kingcroaker *Menticirrhus americanus* (Linnaeus, 1758)(Perciformes: Sciaenidae) in Caraguatatuba Bay, southeastern Brazil. *Brazilian Journal of Oceanography*, 60, 343-352.

Wagner, C. E., McIntyre, P. B., Buels, K. S., Gilbert, D. M., & Michel, E. (2009). Diet predicts intestine length in Lake Tanganyika's cichlid fishes. *Functional Ecology*, 23, 1122-1131.

Walter, J. F., & Austin, H. M. (2003). Diet composition of large striped bass (*Morone saxatilis*) in Cheasapeake Bay. *Fishery Bulletin*, 101, 414.

Winemiller, K. O. (1987). Feeding and reproductive biology of the currito, *Hoplosternum littorale*, in the Venezuelan llanos with comments on the possible function of the enlarged male pectoral spines. *Environmental Biology of Fishes*, 20, 219-227.
